# The Reliability of Biomechanical Mensuration Methods of the Sagittal Cervical Spine in Radiography Used in Clinical Practice: A Systematic Review of the Literature

**DOI:** 10.3390/jcm15166146

**Published:** 2026-08-07

**Authors:** Douglas F. Lightstone, Joseph W. Betz, Jason W. Haas, Paul A. Oakley, Joseph R. Ferrantelli, Ibrahim M. Moustafa, Deed E. Harrison

**Affiliations:** 1 Institute for Spinal Health and Performance, Cumming, GA 30041, USA; douglas.lightstone@gmail.com; 2 Private Practice, Boise, ID 83702, USA; drjoebetz@gmail.com; 3 Chiropractic Biophysics NonProfit, Eagle, ID 83616, USA; drjason@idealspine.com; 4 Kinesiology and Health Science, York University, Toronto, ON M3J 1P3, Canada; docoakley.icc@gmail.com; 5 PostureCo, Trinity, FL 34655, USA; joe.ferrantelli@postureco.com; 6 Neuromusculoskeletal Rehabilitation Research Group, RIMHS—Research Institute of Medical and Health Sciences, University of Sharjah, Sharjah 27272, United Arab Emirates; iabuamr@sharjah.ac.ae; 7 Department of Physiotherapy, College of Health Sciences, University of Sharjah, Sharjah 27272, United Arab Emirates

**Keywords:** cervical spine, reliability, X-ray, radiography, sagittal vertical axis, spine mensuration

## Abstract

**Background:** The biomedical literature assessing the reliability of mensuration of sagittal cervical spine alignment in radiographs has not been systematically evaluated. This review aims to systematically identify and assess reliability studies on biomechanical assessments of the sagittal cervical spine used in clinical practice. **Methods:** The study design was registered with PROSPERO (CRD42023402990). Funding was obtained from Chiropractic BioPhysics (CBP) Non-Profit (Eagle, ID, USA). Inclusion criteria involved studies in English with: human subjects, radiography of the sagittal cervical spine, and reliability analysis of biomechanical mensuration of the sagittal cervical spine. Exclusion criteria involved studies with: geometric modeling, animals, cadavers, phantom mannequins, and non-radiographic studies. PubMed, CINAHL, AltHealthWatch, and Web of Science databases were searched from inception through to 24 January 2023. The quality appraisal tool for studies of diagnostic reliability (QAREL) assessed bias risk. **Results:** We followed the synthesis without meta-analysis (SWiM) according to systematic review guidelines. Scrutiny of the inclusion criteria yielded 51 articles. The results were limited due to the heterogeneity of various mensuration methods (Cobb, posterior tangent, translation measures, etc.) and the incorporation of both manual and digital measured assessments. Other sources of heterogeneity included the quality and type of images used, whether digital or plain film radiographs were used, and the differences in statistical analysis and reporting (ICCs or Pearson correlation coefficients, Cohen’s kappa agreement, or Bland–Altman plots). Still, the preponderance of evidence found good-to-excellent reliability. **Conclusions:** This SROL discovered good-to-excellent intra-examiner reliability for the following biomechanical mensuration methods: intersegmental rotation angles, the thoracic inlet angle, the T1 slope, the C2–C7 posterior tangent method for total cervical lordosis, and the Cobb C2–C7 method for total cervical lordosis. Likewise, using these criteria, we identified good-to-excellent inter-examiner reliability for the following biomechanical mensuration methods: intersegmental rotation angles, the thoracic inlet angle, the T1 slope, translation using the sagittal vertical axis of C2–C7, the C2–C7 posterior tangent method for total cervical lordosis, and the Cobb C2–C7 method for total cervical lordosis.

## 1. Introduction

Cervical spine disorders, such as neck pain (NP), are major contributors to the Global Burden of Disease (GBD) and are the fourth largest contributor to disability worldwide [1]. GBD investigations have found that over 200 million people suffer from cervical spine disorders, with a higher prevalence in females and up to 70% of the population suffering in their lifetime. Most NP, for example, has been reported as acute, but more than 50% of the population who suffer from NP have chronic episodes and frequently report dysfunction due to pain [1]. Patients suffering from chronic NP have reduced productivity and quality of life (QOL) while experiencing increased dysfunction and years lived with disability (YLD), with reports demonstrating 244 for every 100,000 [2].

Cervical spine disorders, including NP, have been studied at great length with regard to mechanisms of injuries, genetic contributions, morphological abnormalities, psychosocial models, and biomechanics, with limited conclusions and consensus on their causes [3]. A recent emergence of biopsychosocial models of cervical spine disorders has been extensively investigated [4,5,6], but oftentimes researchers may neglect or minimize structural and biomechanical contributions [5]. Recent investigations have identified that psychosocial models alone can lack clear explanatory correlations, in as much that fear avoidance and pain catastrophizing models have been found to have poor relationships to chronicity [6]. Understanding the comprehensive aspects of cervical spine disorders should include the role of altered sagittal cervical spine alignment in which interest is growing to investigate its relevance across healthcare disciplines [7,8,9]. Sagittal spinal alignment has been studied since the introduction of X-ray radiography [10]. Studies have found that normal, healthy sagittal cervical biomechanical measurements are desirable and important for improved clinical outcomes [11,12,13,14,15]. Investigations have identified that alterations in sagittal alignment have a significant impact on patient QOL, well-being, pain, and disability, and these findings are consistent across recent systematic reviews with meta-analysis [7,9,13,14].

Reliable methods for biomechanical mensuration of the cervical spine are essential components for assessing cervical spines in patients with altered sagittal alignment [11,12,13,14,15,16,17,18,19,20,21]. The analysis of sagittal cervical configuration and lordosis are imperative in spinal surgery, with the current techniques making use of vertebral segmental and global rotations and translations, sagittal balance, cranio-cervical measurements, and skull-to-thorax radiographic parameters [7,13,22]. Recent studies have shown that unhealthy sagittal cervical balance is a prognostic indicator for improvement post-surgery [7,12,23]. Sagittal cervical spinal mensuration and assessment are also necessary for conservative spinal rehabilitation interventions, such as those which require spinal therapeutic exercises and lordotic restorative traction forces [15,18].

There are various methods of biomechanical analysis of cervical spinal alignment using radiography. That said, no systematic reviews of the literature (SROLs) were identified specifically assessing the reliability of these methods using radiography. Given the significant impact of cervical spine disorders on GBD and YLD [1,2] and the impact that altered sagittal alignment has on patient outcomes [7,9,13,14], evaluating the reliability of cervical spine radiographic biomechanical analysis would help determine the best mensuration methods. The aim of this SROL is to document the extent of scientific literature reporting on intra- and inter-examiner reliability of biomechanical assessments of the sagittal cervical spine in radiography used in clinical practice. The secondary goals of this SROL are to:

- Assess the quantitative and qualitative intra- and inter-examiner reliability of biomechanical mensuration methods for the sagittal cervical spine in radiography.
- Appraise the bias risk and quality of reliability studies.
- Compare the reliability of various biomechanical mensuration methods.
- Document the strengths and limitations of reliability studies from the current scientific literature, and provide recommendations for future research on this topic.

The study hypothesizes that the available literature detailing intra- and inter-examiner reliability of biomechanical assessments of the sagittal cervical spine in radiography used in clinical practice provides a sufficiently low bias risk, with moderate-to-high-quality studies that establish good or better reliability.

## 2. Materials and Methods

**Figure 1:**
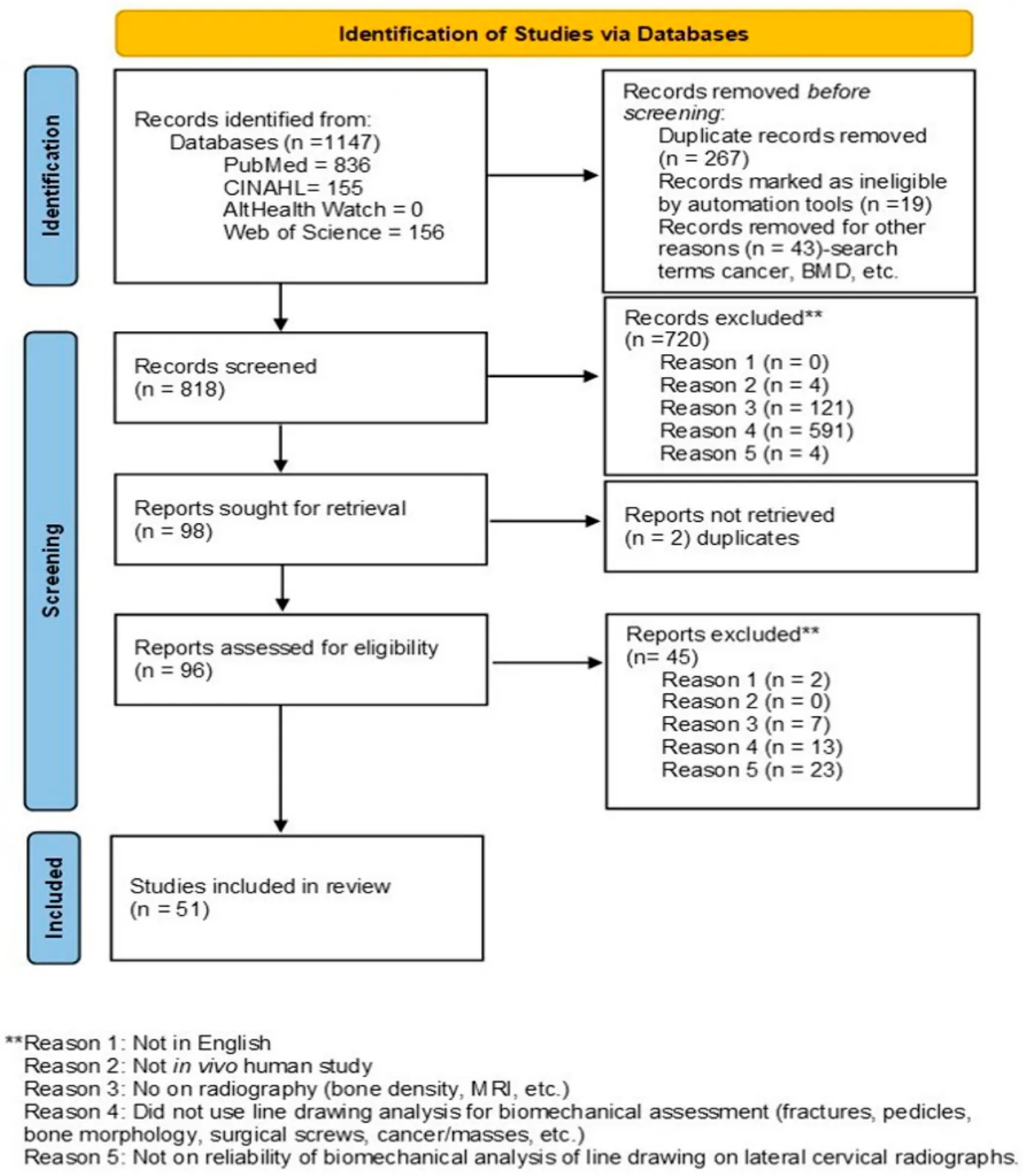
Preferred reporting items for systematic review and meta-analyses (PRISMA) flow diagram [24].

Prior to the article search, the design of this SROL was registered with PROSPERO (CRD42023402990) on 10 March 2023, and can be found at https://www.crd.york.ac.uk/prospero/ (accessed on 31 January 2026) in accordance with the standards of systematic review reporting. The PRISMA 2020 flow design and statement were initially created specifically for the effects of health interventions and encouraged multiple other SROL application to also follow this design The use of PRISMA confirms the timeline of this review, the interpretation and presentation by its reviewers, and the results that were found. This investigation, interpretation, presentation, and report were all in accordance with PRISMA checklists [24] (Supplementary Material Table S1). Figure 1 outlines the preferred reporting items for systematic review and meta-analyses (PRISMA) flow diagram for selection, inclusion, and exclusion of articles used in this SROL.

### 2.1. Systematic Review Search Strategy

PRISMA checklists were used to outline the search and interpretation process for this SROL. This strategy utilized patient/population, intervention, comparison, and outcomes (PICO) as an alternative approach. The PICO alternative design was followed in accordance with the search strategy from the peer review of electronic search strategies (PRESS) checklist [25]. A Master of Library Science and a librarian searched each of the following databases: PubMed, AltHealthWatch, CINAHL, and Web of Science. Searches were conducted from the initial date of database inception through to 24 January 2023.

The search terms for PubMed were: “Cervical vertebrae/diagnostic imaging” (Medical Subject Headings (MeSH) Terms) OR “Cervical” AND “Spinal diseases/diagnostic imagining” (MeSH Terms) AND “reproducibility of results” (MeSH Terms) OR “reference values” (MeSH Terms). “Cervical vertebrae/diagnostic imaging” OR “Cervical” AND “spinal diseases/diagnostic imagining” (MeSH Terms) AND (chiropractic MeSH terms) or “manipulation, chiropractic”.

The search terms for CINAHL were: MH “Spinal diseases/RA” AND (Cervical) OR (MH “Cervical vertebrae/RA” AND (MH “reproducibility of results” OR (MH “Reference Values”. MH “Chiropractic” OR (MH Spinal diseases/RA” AND (Cervical) OR (MH “Cervical vertebrae/RA”.

The search terms for AltHealthWatch were: (DE “Cervical vertebrae”) AND (DE “RADIOGRAPHY) OR DE “MEDICAL Radiography” and DE “REPRODUCIBLE research” OR DE “REFERENCE values”. DE “CHIROPRACTIC” OR (DE CHIROPRACTIC treatment for infant diseases” OR (DE CHIROPRACTIC treatment for juvenile diseases” OR (DE Spinal RADIOGRAPHY in chiropractic” OR (DE “SPINAL adjustment” AND (DE CERVICAL vertebrae) AND (DE “RADIOGRAPHY”) OR DE (MEDICAL radiography).

The search terms for Web of Science were: (TS = Cervical) AND (TS = Reference values) OR (TS = reliability of results) AND (TS = X-ray) OR (TS = radiography), (TS = Cervical) AND (TS = chiropractic). 

**Figure 2:**
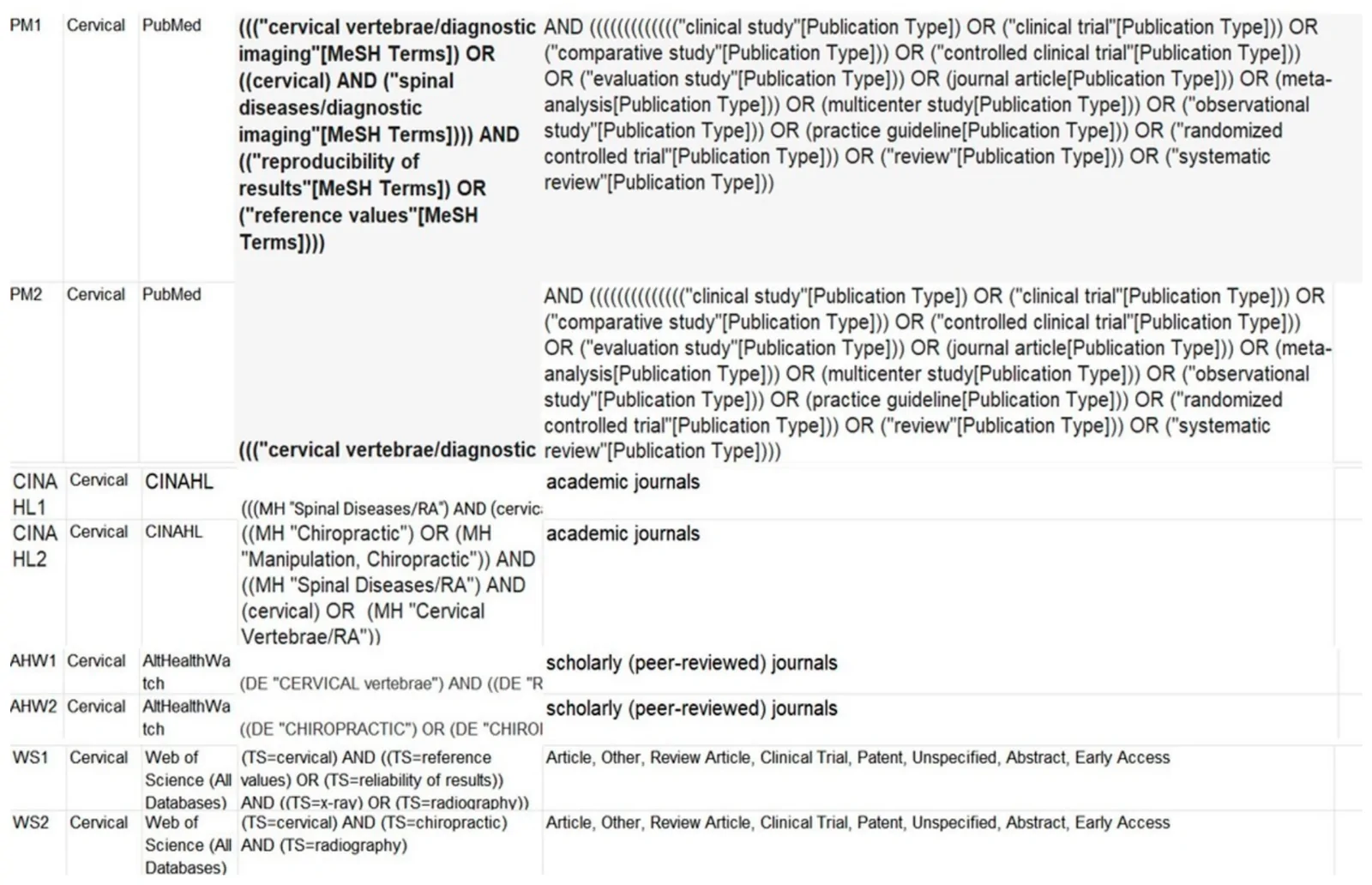
Expanded SROL search and MeSH terms performed by a Master of Library Science.

The search MeSH terms utilized are expanded upon in Figure 2.

### 2.2. SROL Inclusion and Exclusion Criteria

The inclusion criteria for the search strategy for this SROL required that publications studied the reliability of a biomechanical mensuration method of the sagittal cervical spine using radiography. Each study must have used radiography of the sagittal cervical spine on either asymptomatic participants or participants with defined cervical spine conditions, and have also included the use of biomechanical mensuration of sagittal cervical spine. The exclusion criteria for this SROL were as follows:

- Criterion 1: The study was not in English. We note that a search was performed for the translations of manuscripts that were not in English and were not excluded if an English translation was found.
- Criterion 2: The study was not an in vivo human study.
- Criterion 3: The study was not about radiography.
- Criterion 4: The study was not about line drawing or mensuration for biomechanical analysis.
- Criterion 5: The study was not on the reliability of biomechanical analysis of line drawing on lateral cervical radiographs.

**Figure 3:**
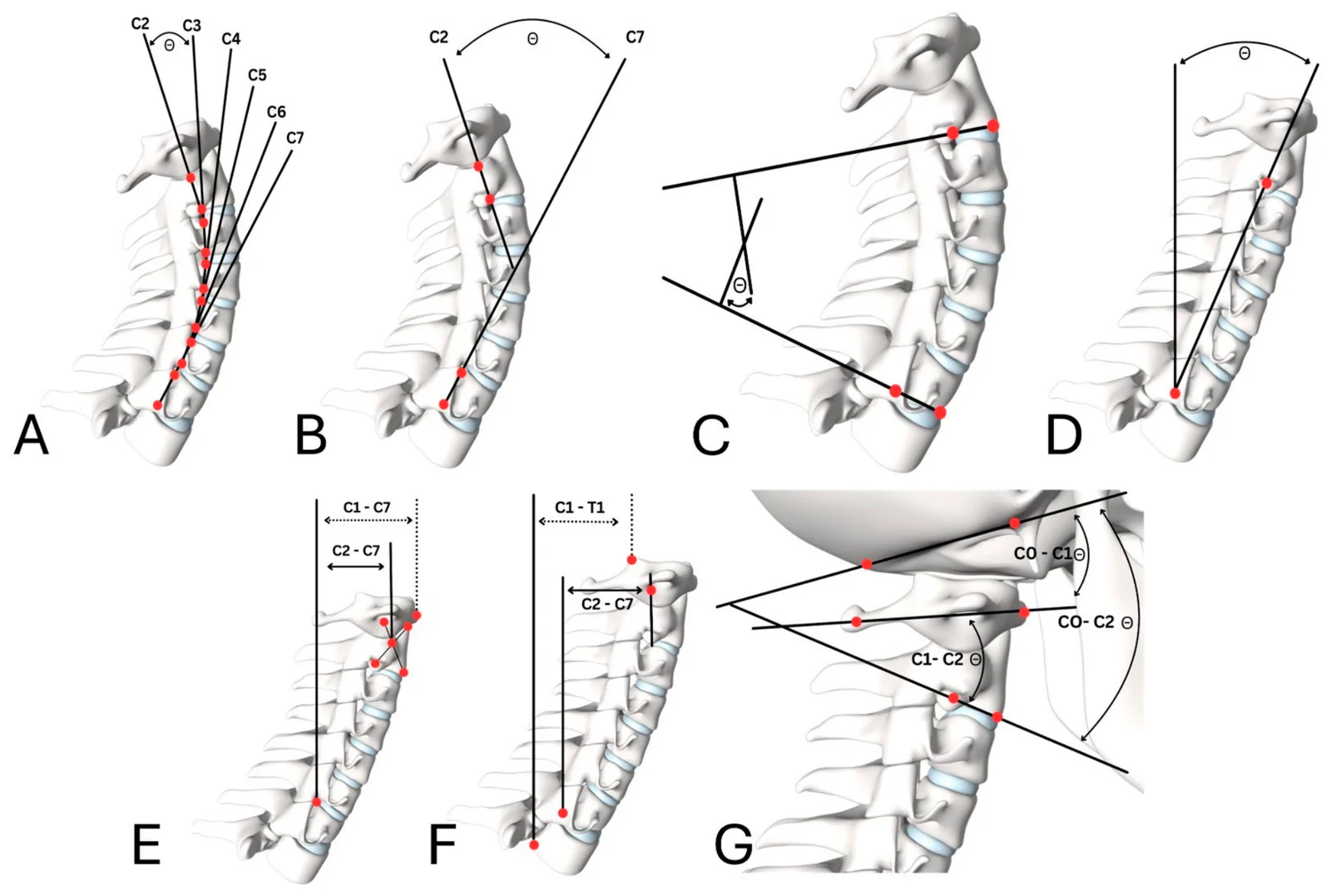
Examples of biomechanical mensuration methods of the sagittal cervical spine using radiography in clinical practice. For a detailed reference list of each measurement method and its reliability, see Supplementary Tables S1–S3. (**A**) The Harrison posterior tangent method, with absolute rotation angle from C2 to C7 vertebrae (ARA C2–C7) and intersegmental relative rotational angles (RRAs) from C2 to C7 (**B**) posterior body tangent lines method for measuring C2–C7 curvature; (**C**) sagittal cervical Cobb angle of C2–C7; (**D**) sagittal cervical K-line tilt (**E**) sagittal vertical axis from C2 to C7 (SVA C2–C7), with the horizontal offset of the centroid of C2 relative to a vertical line drawn superiorly from the posterior superior corner of C7; (**F**) SVA C1–T1, using the horizontal offset of the posterior superior corner of C1 lateral mass relative to a vertical line from the posterior inferior body corner of T1, and SVA C2–C7 from the posterior inferior body corner of C7 relative to the posterior superior corner of C2; and (**G**) C0–C2 Cobb angle and C1–C2 Cobb angle.

All non-radiographic studies and studies that did not feature radiographic biomechanical mensuration parameters of the lateral cervical spine were excluded from the final manuscript reviewal process. Since there are a multitude of methods for measuring cervical vertebral displacement, all measurement methods were included. Figure 3 depicts some of the more commonly used methods.

### 2.3. Selection of Studies for Systematic Review

The initial search strategy screened the abstracts and titles following the inclusion and exclusion criteria outlined above. The use of automation tools allowed for further screening of studies to determine if there were duplicates or if there was a difference found between the original database and the results. Authors D.F.L. and J.W.B. screened all abstracts and studies based on the inclusion and exclusion criteria, and discrepancies or disagreements were settled by a third reviewer (J.W.H.), resulting in consensus. The articles that met the inclusion criteria with no exclusion criteria were used in the formulation of the results of this SROL.

### 2.4. Review Study Characteristics and Data Collection

**Figure 4:**
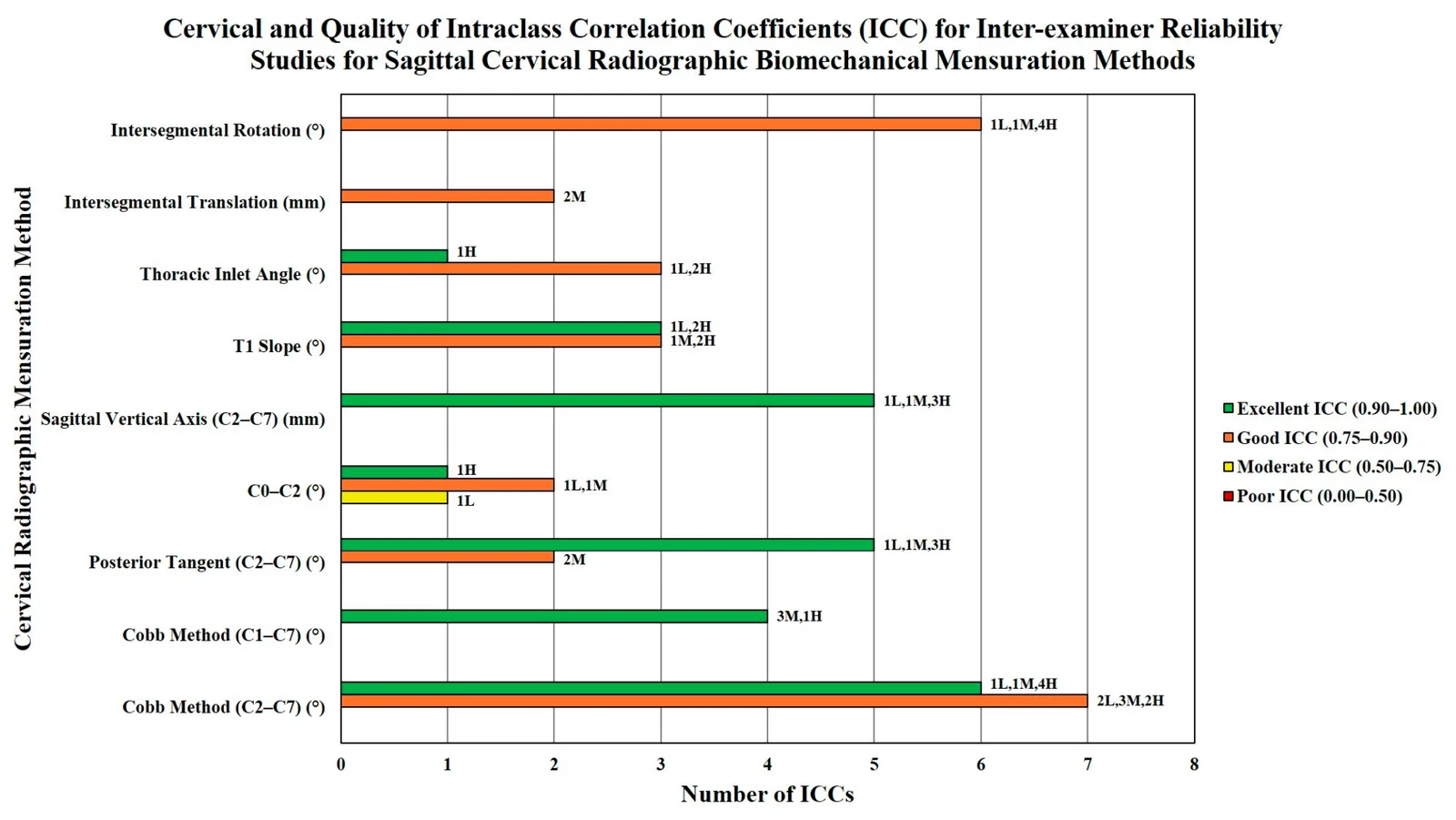
Quality of ICC of inter-examiner reliability studies on sagittal cervical radiographic biomechanical methods. This cluster bar chart represents the quality of reliability, quality of studies, and number of studies found for inter-examiner reliability of sagittal cervical radiographic biomechanical mensuration methods. Each mensuration method (*y*-axis) may have up to four bars showing the number of ICCs (*x*-axis) for each cervical radiographic mensuration method (*y*-axis), representing the quality of ICCs (excellent, good, moderate, or poor; see legend for color). Data labels on the outer-right end of bars represent the numbers of low (L)-, moderate (M)-, and high (H)-quality studies (as determined using the QAREL instrument) that comprise the total number of studies for a given ICC quality (excellent, good, moderate, or poor). For example, the posterior tangent method (C2–C7) shows five excellent inter-examiner ICCs (comprising one moderate-quality study, one low-quality study, and three high-quality studies). The unit of measurement (° or mm) for each cervical radiographic biomechanical mensuration method is indicated in parentheses next to the mensuration method.

**Figure 5:**
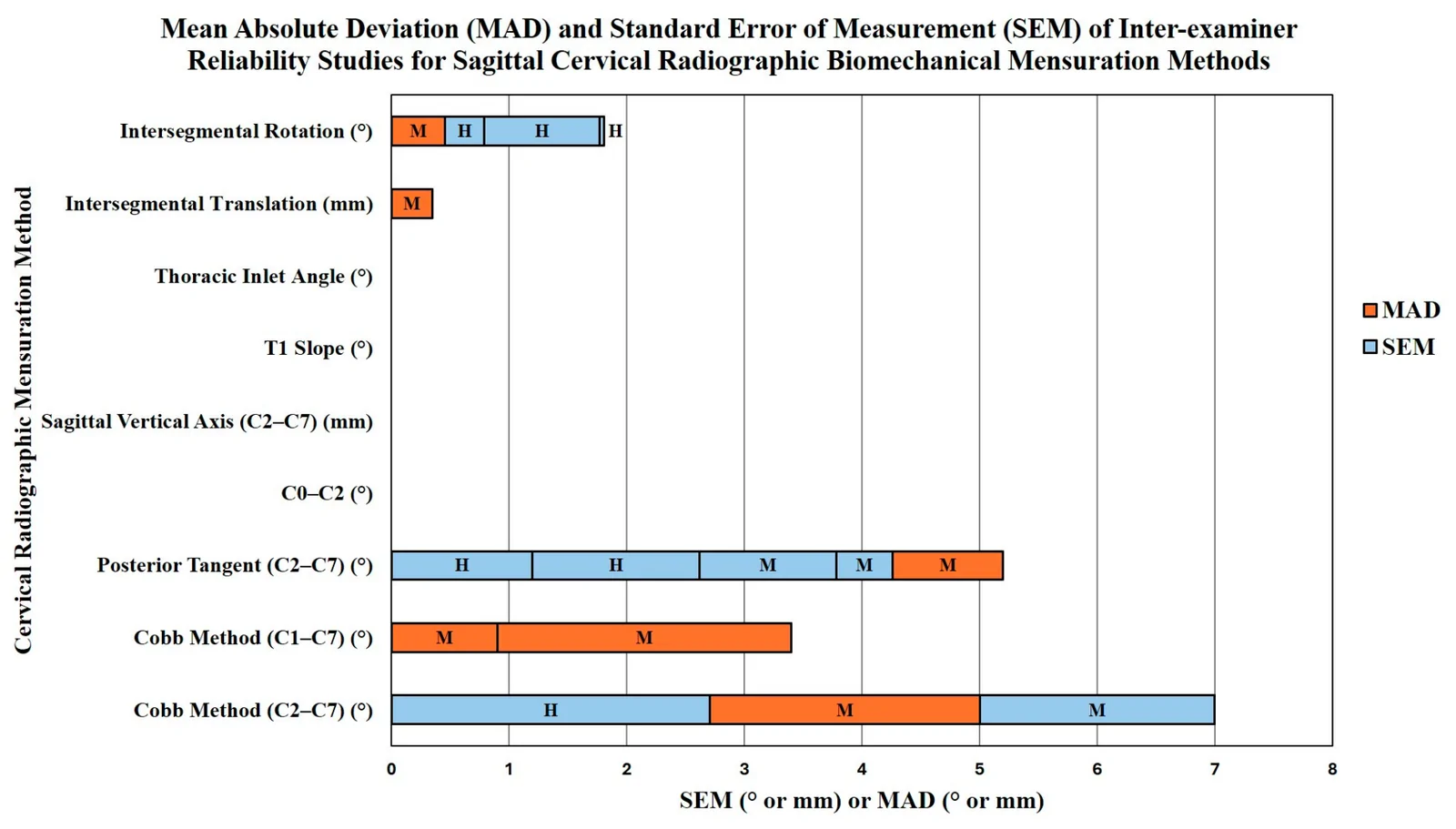
MAD and SEM of inter-examiner reliability studies for sagittal cervical radiographic biomechanical mensuration methods. This is a stacked bar chart representing the mean absolute deviation (MAD) and standard error of measurement (SEM) found in inter-examiner reliability studies for sagittal cervical radiographic biomechanical mensuration methods. The stacked bars show the number of MAD and SEM (*x*-axis; see legend for color) for each cervical radiographic mensuration method (*y*-axis), and the letter in each bar represents the quality of the study, as determined by the QAREL instrument (M = moderate quality, and H = high quality). The unit of measurement (° or mm) for each cervical radiographic biomechanical mensuration method is indicated in parentheses next to the mensuration method.

In the final screening, authors D.F.L. and J.W.B. screened the full-text articles, retrieved by the Master of Library Science, based on the inclusion and exclusion criteria, and discrepancies or disagreements were settled by a third reviewer (J.W.H.), resulting in consensus. Authors J.R.F. and D.F.L. created spreadsheets and files for interpretation in Microsoft Excel (Table S2). Information was then extracted from the studies, including the authors and year of study, sample number and demographic information, number of examiners, repeat analysis details, types of imaging, methods of analysis, intra- and/or inter-examiner reliability analyses, and SEMs and/or MADs. No assumptions were made regarding missing or unclear information. Missing data was recorded as ‘N’ for no in data tables (Table 1). The studies were then assessed for risk of bias, study quality, intra- and inter-examiner reliability statistical analyses, and SEMs and MADs (Figure 4 and Figure 5, Table S3 and S4).

**Figure 6:**
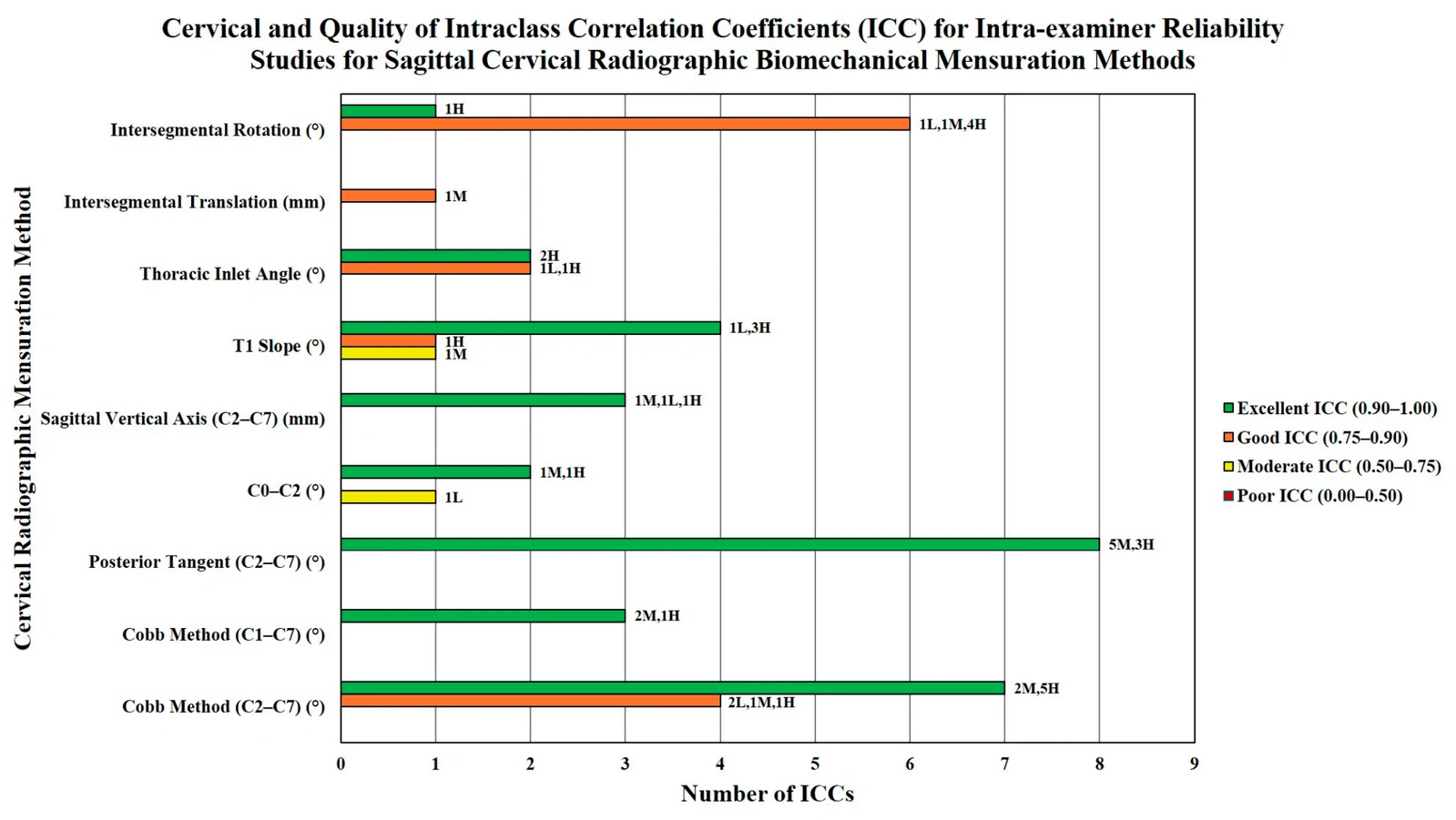
Quality of ICCs of intra-examiner reliability studies for sagittal cervical radiographic biomechanical methods. This cluster bar chart represents the quality of reliability, quality of studies, and number of studies found for intra-examiner reliability of sagittal cervical radiographic biomechanical mensuration methods. Each mensuration method (*y*-axis) may have up to four bars showing the number of ICCs (*x*-axis) for each cervical radiographic mensuration method (*y*-axis), representing the quality of ICCs (excellent, good, moderate, or poor; see legend for color). Data labels at the outer-right end of the bars represent the number of low (L)-, moderate (M)-, and high (H)-quality studies (as determined using the QAREL instrument) that comprise the total number of studies for a given ICC quality (excellent, good, moderate, or poor). For example, the posterior tangent method (C2–C7) shows eight excellent intra-examiner ICCs (comprising five moderate-quality studies and three high-quality studies). The unit of measurement (° or mm) for each cervical radiographic biomechanical mensuration method is indicated in parentheses next to the mensuration method.

**Figure 7:**
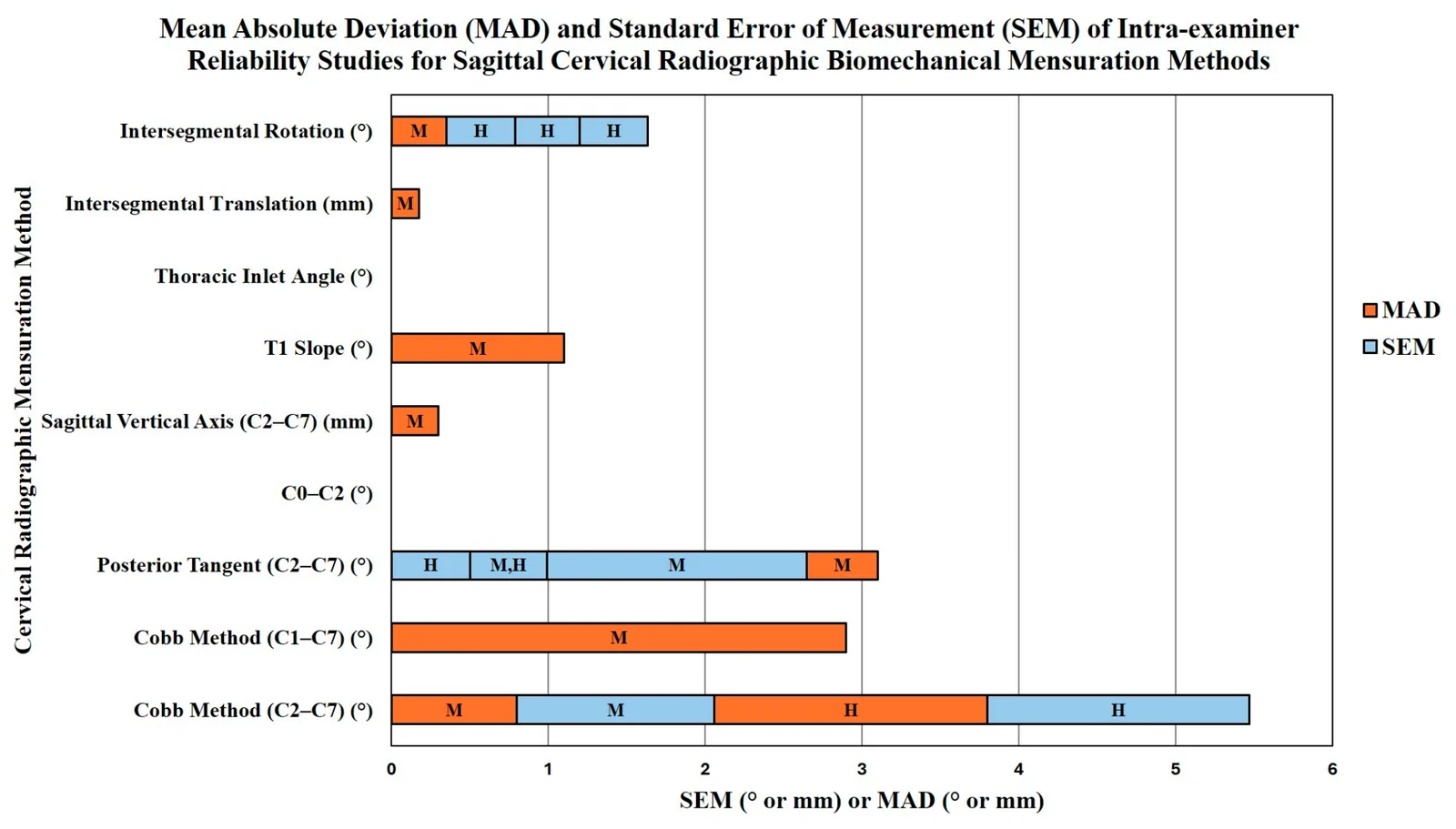
MAD and SEM of intra-examiner reliability studies for sagittal cervical radiographic biomechanical mensuration methods. This is a stacked bar chart representing the mean absolute deviation (MAD) and standard error of measurement (SEM) found in inter-examiner reliability studies for sagittal cervical radiographic biomechanical mensuration methods. The stacked bars show the number of MAD and SEM (*x*-axis; see legend for color) for each cervical radiographic mensuration method (*y*-axis), and the letter in each bar represents the quality of the study, as determined by the QAREL instrument (M = moderate quality, and H = high quality). The unit of measurement (° or mm) for each cervical radiographic biomechanical mensuration method is indicated in parentheses next to the mensuration method.

All statistical analyses for intra- and/or inter-examiner reliability, as well as the results of any statistical analyses, were recorded (Figure 4, Figure 5, Figure 6 and Figure 7, Tables S2 and S3). Statistical analytical methods for reliability assessments included intra- and inter-rater ICCs, Pearson correlation coefficients, Cohen’s kappa agreement, and Bland–Altman plots, all of which can have different reliability assessments and interpretations [77].

### 2.5. Evaluation for Study Quality Assessment and Review Study Bias Assessment

The QAREL instrument used with reliability studies was confirmed and approved for this study by the authors. The assessment, described by Alfuth [78] and Konieczka et al. [79], was used to provide an assessment of risk of bias and study quality [80]. Following the assessment by two initial reviewers (J.R.F. and D.F.L.), for any bias assessment of the studies, a third reviewer (J.W.H.) confirmed the bias risk and re-assessed confirmation with the inclusion criteria. Utilization of this multi-step process for the review of articles originally acquired allowed for significantly more critical appraisal of the data collected, and offered a much greater clinical application of the findings than selective, snap, or limited reviews.

### 2.6. Conglomerated Study Synthesis and Data Analysis

The three-reviewer process, incorporating two initial assessments of data followed by a third referee review, was used to reduce bias in data collection and improve systematic review quality and credibility. The three-reviewer process resolved any discrepancies, including with missing data or missed duplication errors. It has been shown from prior studies that the use of multiple reviewers increases the quality and accuracy of data collection and synthesis in a SROL [81]. Prior studies have recommended the use of SWiM guidelines for SROL sub-group analysis to improve the confidence and certainty of conclusions and findings [82]. Herein, for a specific line drawing measurement method to be rated ‘good-to-excellent’ for reliability, a minimum of 2 low-risk/high-quality studies that find good-to-excellent examiner reliability are needed.

## 3. Results

### 3.1. Systematic Review Selection Process and Search Terms

The discovery process yielded a total of 1147 potential articles. A deduplication tool in EndNote [83] was used for duplicate articles removal. Ineligible articles using automation were removed. Anomalous, pathological, and other conditions resulted in article removal. This resulted in 818 articles after filtering titles and abstracts for the following medical subject heading (MeSH) terms:

### 3.2. Further Screening of Search Results and Inclusion and Exclusion Criteria

The databases searched were PubMed (n = 836), the Cumulative Index to Nursing and Allied Health Literature (CINAHL) (n = 155), AltHealthWatch (n = 0), and Web of Science (N = 156). Manual and digital deduplication assessment removed 267 articles. A total of 19 articles that were found to use automation tools removed. Other records were removed if they were found to contain assessments of pathologies, anomalies, cancers, malignancies, bone mineral densities, and non-pertinent conditions (n = 43). Following this assessment for record removal prior to screening, 720 articles were removed, leaving 818 studies. The exclusion criteria for the remaining articles were as follows:

▪ Criterion 1: The study was not in English (n = 3). We note that a search was made for the translations of manuscripts that were not in English and were not excluded if an English translation was found (n = 0).
▪ Criterion 2: The study was not an in vivo human study (n = 4).
▪ Criterion 3: The study was not about radiography (n = 121).
▪ Criterion 4: The study was not about line drawing or mensuration for biomechanical analysis (n = 591).
▪ Criterion 5: The study was not on the reliability of biomechanical analysis of line drawing using lateral cervical radiographs (n = 4).

The 98 articles remaining following this search were sought for retrieval, screening, and data analysis by two independent reviewers, with a third referee reviewer available to resolve discrepancies and disagreements. This retrieval found two duplicates, and these studies were removed from further analysis. This brought the total articles which met all screening criteria for the SROL and of those remaining 96 were found by the reviewers to completely meet the study criteria and 45 were excluded. The final exclusions found were: criterion 1 (n = 2), criterion 2 (n = 0), criterion 3 (n = 7), criterion 4 (n = 13), criterion 5 (n = 22). Figure 1 demonstrates the preferred reporting items for systematic review and meta-analyses (PRISMA) flow diagram for selection, inclusion and exclusion used in this SROL. The independent reviewers D.F.L. and J.W.B. then reviewed the remaining 96 articles according to the inclusion and exclusion criteria. Discrepancies or disagreements were resolved by the third reviewer J.W.H. This additional full-text review found agreement on the exclusion of 45 additional studies according to criterion 1 (n = 2), criterion 2 (n = 0), criterion 3 (n = 7), criterion 4 (n = 13), and criterion 5 (n = 22). The final total number of articles meeting all criteria was 51.

### 3.3. Final 51 Article Characteristics

Table S2 reports the study characteristics and findings of the 51 studies meeting all inclusion and exclusion criteria. The details in the study group recorded the number of investigators, techniques used for repeat analysis, methods of mensuration, and parameters evaluated, as well as the statistical results for all studies. The studies were analyzed independently by reviewers J.W.B. and D.F.L., and any discrepancies or disagreements were resolved by reviewer J.W.H.

### 3.4. QAREL Instrument Analysis for Bias and Quality Assessment

The 11-item quality appraisal tool for studies of diagnostic reliability (QAREL) instrument was used to assess the studies for their bias risk and quality (Table 1). We used a previously reported scoring system by Alfuth [25] and Konieczka [79]. This table breaks the final 51 studies of the SROL down by their answers to the QAREL instrument to assess quality, bias, and reliability [26,27,28,29,30,31,32,33,34,35,36,37,38,39,40,41,42,43,44,45,46,47,48,49,50,51,52,53,54,55,56,57,58,59,60,61,62,63,64,65,66,67,68,69,70,71,72,73,74,75,76]. Each reliability study’s lead author and date of publication are listed on the left with the reference number, followed by the answers to the 11 QAREL questions, the sum of the affirmative answers, the risk of bias of the study, the quality of the study, and the type of reliability assessed within the study.

**Figure 8:**
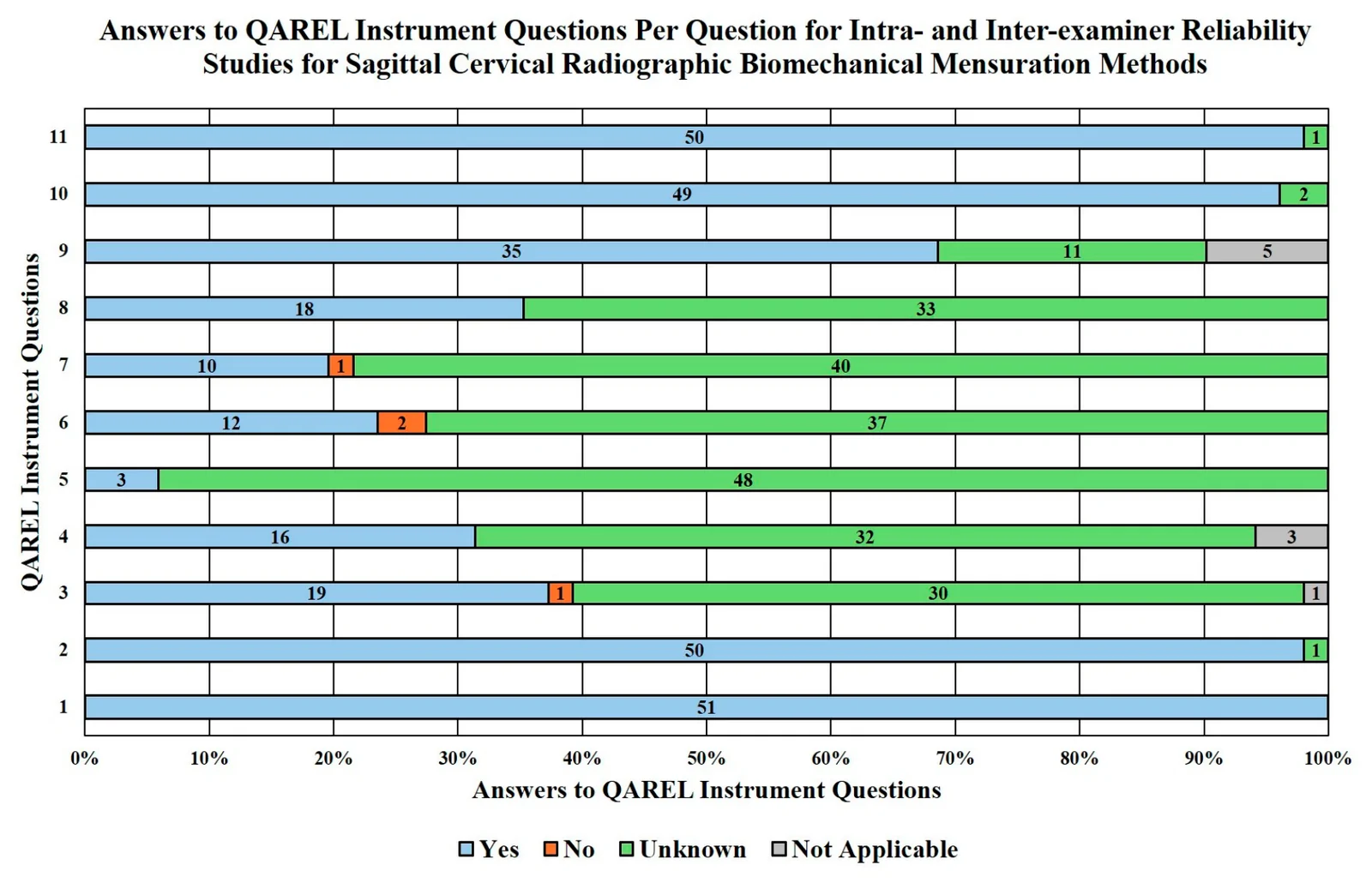
Answers to QAREL instrument questions per question for intra- and inter-examiner reliability studies for sagittal cervical radiographic biomechanical mensuration methods. This is a stacked bar chart representing the answers to QAREL instrument questions per question for intra- and inter-examiner reliability studies for sagittal cervical radiographic biomechanical mensuration methods. The stacked bars represent the percentage of studies (and show the actual number on the bar) that provided the corresponding answer (*x*-axis; see legend for color) per QAREL instrument questions (*y*-axis).

The answers to the QAREL instrument were also assessed (Figure 8, Table 2). The QAREL instrument most answered questions in the studies included: 1. Was the sample of subjects representative? (51/51 = 100.0% yes); 2. Was the sample of raters representative? (50/51 = 98.0% yes); 10. Was the test applied correctly and interpreted correctly? (49/51 = 96.1% yes); and 11. Were appropriate statistical measures of agreement used? (50/51 = 98.0% yes). The QAREL instrument least answered questions in the studies included: 4. Were raters blinded to their own prior findings? (16/51 = 31.4% yes); 5. Were raters blinded to the accepted reference standard? (3/51 = 5.9% yes); 6. Were the raters blinded to the clinical information not part of the test? (12/51 = 23.5% yes); and 7. Were raters blinded to additional non-clinical cues? (10/51 = 19.6% yes). Three out of 11 questions (questions 5–7) on the QAREL instrument had less than a 30% yes response. It is not known whether this is a systemic problem with reliability studies in general or if this is just something reports generally ignore. Since the QAREL instrument uses the total score as the standard for rating studies, it is not known how weighting differences in these three questions influence the specific overall study rankings.

**Figure 9:**
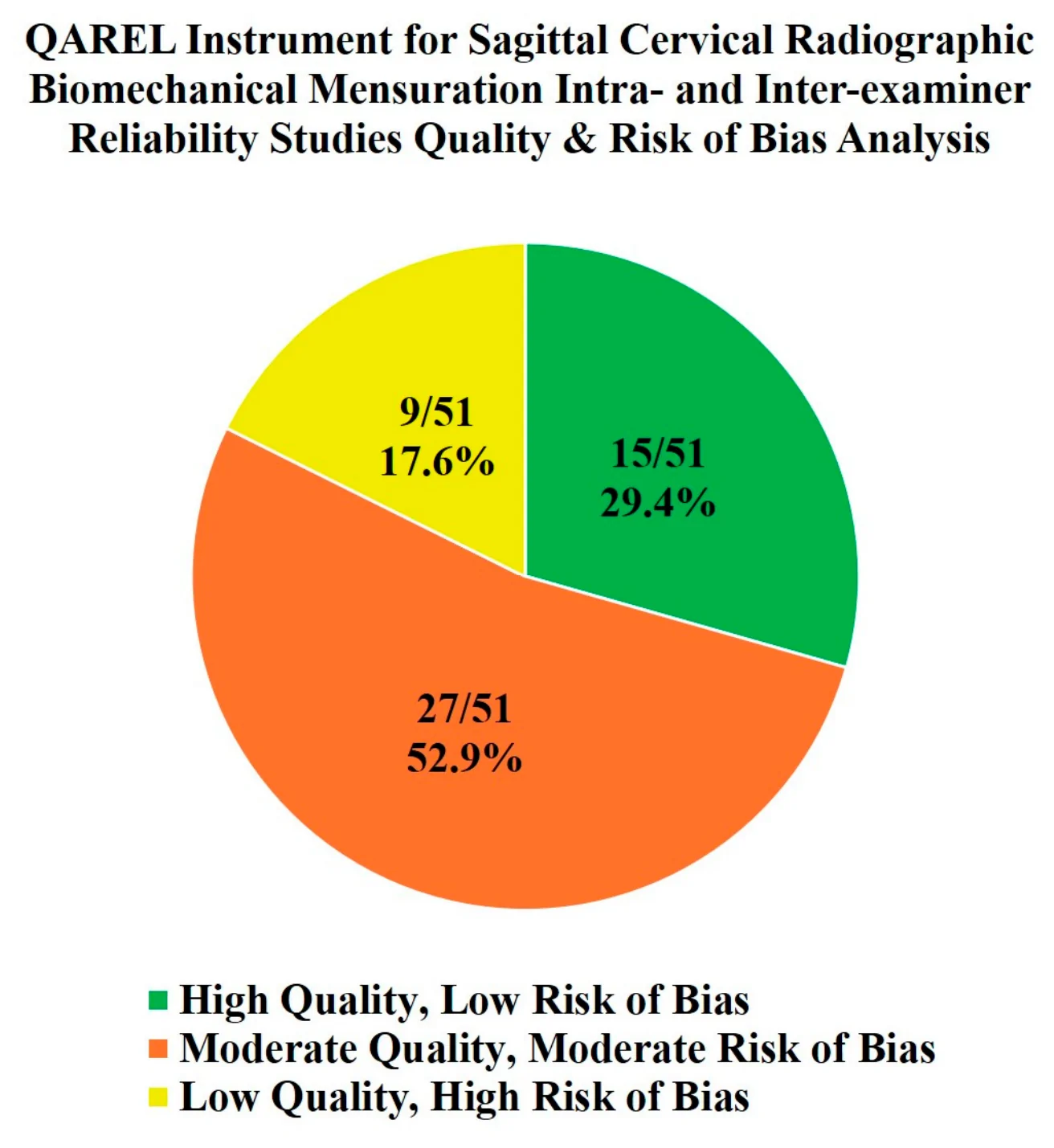
Sagittal cervical radiographic biomechanical mensuration intra- and inter-examiner reliability studies’ quality and risk-of-bias analysis using the QAREL instrument. No rounding was applied which accounts for the 0.1% discrepancy.

When assessing intra- and inter-examiner reliability (n = 51) of the articles included in the SROL, it was found that, following assessment, 15 (29.4%) articles had a low risk of bias (high quality), 27 (52.9%) articles were found to have a moderate risk of bias (moderate quality), and nine (17.6%) studies were found to have a high risk of bias (low quality) (Figure 9).

**Figure 10:**
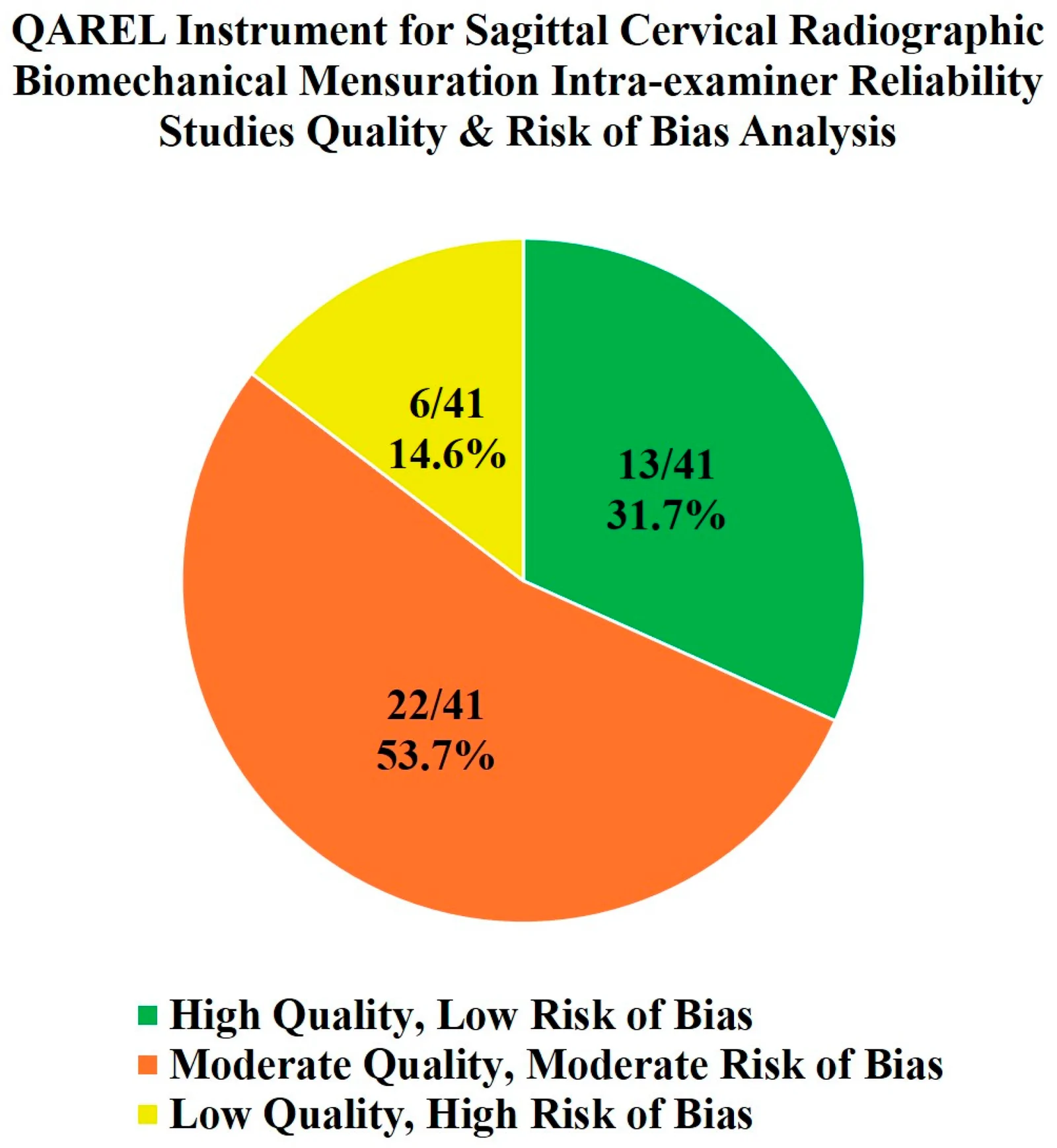
Sagittal cervical radiographic biomechanical mensuration intra-examiner reliability studies’ quality and risk-of-bias analysis using the QAREL instrument.

When assessing intra-examiner reliability (n = 41) of the articles included in the SROL, it was found that, following assessment, 13 (31.7%) articles had a low risk of bias (high quality), 22 (53.7%) articles were found to have a moderate risk of bias (moderate quality), and six (14.6%) studies were found to have a high risk of bias (low quality) (Figure 10).

**Figure 11:**
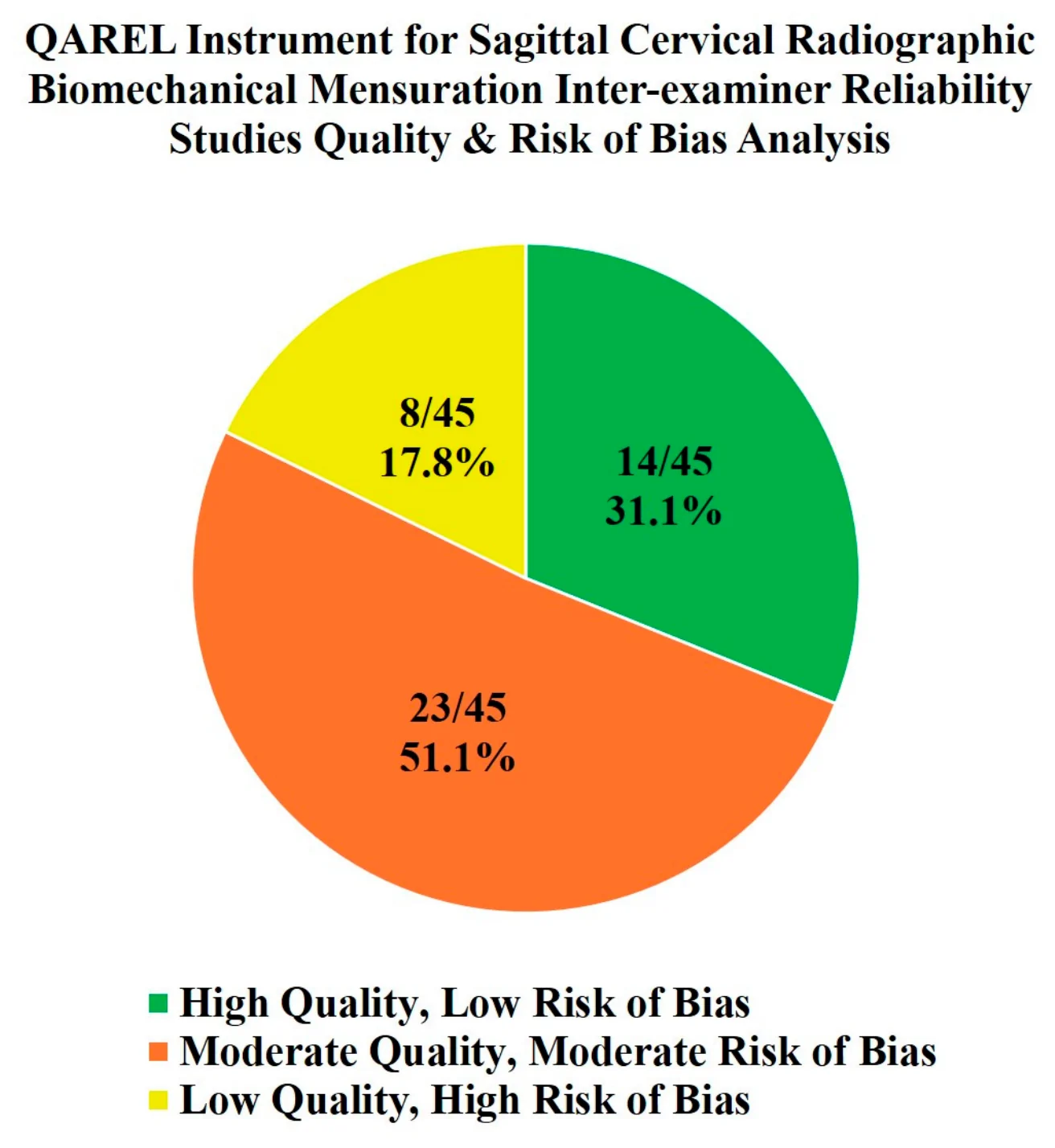
Sagittal cervical radiographic biomechanical mensuration inter-examiner reliability studies’ quality and risk-of-bias analysis using the QAREL instrument.

When assessing inter-examiner reliability (n = 45) of the articles included in the SROL, it was found that, following assessment, 14 (31.1%) articles had a low risk of bias (high quality), 23 (51.1%) articles were found to have a moderate risk of bias (moderate quality), and eight (17.8%) studies were found to have a high risk of bias (low quality) (Figure 11).

Inter-examiner reliability studies using the Cobb C2–C7 method reported seven good and six excellent intraclass correlation coefficients (ICCs). Inter-examiner reliability studies using the Cobb C1–C7 method reported four excellent ICCs. Inter-examiner reliability studies using the posterior tangent (C2–C7) method reported two good and five excellent ICCs. Inter-examiner reliability studies using C0–C2 reported one moderate, two good, and one excellent ICCs. Inter-examiner reliability studies using the sagittal vertical axis (C2–C7) reported five excellent ICCs. Inter-examiner reliability studies using the T1 slope reported three good and three excellent ICCs. Inter-examiner reliability studies using the thoracic inlet reported three good and one excellent ICCs. Inter-examiner reliability studies using intersegmental translation reported two good ICCs. Inter-examiner reliability studies using intersegmental rotation reported six good ICCs (Figure 4, Table S4). According to Figure 4, the following line methodology was found to be rated ‘good-to-excellent’ for **inter**-examiner reliability:

(a) Intersegmental rotation angle had four high-quality studies with good reliability.
(b) The thoracic inlet angle had three high-quality studies, with two showing good reliability and one as excellent.
(c) The T1 slope had four high-quality studies, with two showing good reliability and two showing excellent reliability.
(d) The sagittal vertical axis had three high-quality studies with excellent reliability.
(e) The posterior tangent C2–C7 had three high-quality studies with excellent reliability.
(f) The Cobb C2–C7 method had six high-quality studies, with two showing good reliability and four showing excellent reliability.
(g) While the methods of measurement for intersegmental translation, C0–2, and Cobb C1–C7 did not reach the high rated standard, they still would be rated as moderate quality with moderate reliability.

Intra-examiner reliability studies using the Cobb C2–C7 method reported four good and seven excellent intraclass correlation coefficients (ICCs). Intra-examiner reliability studies using the Cobb C1–C7 method reported three excellent ICCs. Intra-examiner reliability studies using the posterior tangent (C2–C7) method reported eight excellent ICCs. Intra-examiner reliability studies using C0–C2 reported one moderate and two excellent ICCs. Intra-examiner reliability studies using the sagittal vertical axis (C2–C7) reported three excellent ICCs. Intra-examiner reliability studies using the T1 slope reported one moderate, one good, and four excellent ICCs. Intra-examiner reliability studies using the thoracic inlet reported two good and two excellent ICCs. Intra-examiner reliability studies using intersegmental translation reported one good ICC. Intra-examiner reliability studies using intersegmental rotation reported six good and one excellent ICCs (Figure 6, Table S3). According to Figure 6, the following methodology was found to be rated ‘good-to-excellent’ for intra-examiner reliability:

(a) Intersegmental rotation angle had five high-quality studies, with four showing good reliability and one as excellent.
(b) The thoracic inlet angle had three high-quality studies with one showing good reliability and two showing excellent reliability.
(c) The T1 slope had three high-quality studies with excellent reliability.
(d) The posterior tangent C2–C7 method had three high-quality studies with excellent reliability.
(e) The Cobb C2–C7 method had six high-quality studies with one showing good reliability and five showing excellent reliability.
(f) While the methods of measurement for intersegmental translation, sagittal vertical axis (C2–C7), C0–2, and Cobb C1–C7 did not reach the high rated standard, they still would be rated as moderate quality with moderate reliability.

### 3.5. Intra- and Inter-Examiner Reliability with SEM and MAD

Assessment of intra-examiner reliability studies for standard error of measurement (SEM) and mean absolute distance (MAD) found the studies of moderate-to-high quality reported ranges of SEM in angular measurements from 0.5 to 5.47° and MAD in angular measurements from 0.35 to 3.8°, with translation measurements ranging from 0.175 to 0.3 mm (Figure 7, Table S3).

The biomechanical mensuration method that showed the lowest SEM of angular measurements was the posterior tangent (C2–C7) method with 0.50°. There were no SEMs reported for translational measurements. The biomechanical mensuration method that showed the highest SEM of angular measurements was the Cobb method (C2–C7) with 5.47°. The biomechanical mensuration method that showed the lowest MAD of angular measurements was intersegmental rotation with 0.35°. The biomechanical mensuration method that showed the lowest MAD of translation measurements was intersegmental translation with 0.175 mm.

The biomechanical mensuration method that showed the highest SEM of angular measurements was the Cobb method (C2–C7) with 3.8°. There were no SEMs reported for translational measurements. The biomechanical mensuration method that showed the highest MAD of angular measurements was the Cobb method (C2–C7) with 3.8°. The biomechanical mensuration method that showed the highest MAD of translation measurements was the sagittal vertical axis (C2–C7) with 0.3 mm.

The biomechanical mensuration method that showed the greatest precision of SEMs across all studies in angular measurements was intersegmental rotation, with SEMs ranging from 0.79 to 1.635°. The Cobb method (C2–C7) was the only angular measurement method with more than one study with MADs reported, which ranged from 0.8 to 3.8°. All translation measurement methods with MADs only had one study. The biomechanical mensuration method that showed the least precision of SEMs across all studies in angular measurements was the Cobb method (C2–C7), with SEMs ranging from 2.06 to 5.47°.

Assessment of inter-examiner reliability studies for the mean absolute distance (MAD) and standard error of measurement (SEM) found that the studies of moderate-to high-quality reported ranges of SEM in angular measurements from 0.79 to 7.0° and MAD in angular measurements from 0.455 to 5.2°, with translation measurements of 0.345 mm (Figure 5, Table S4).

The biomechanical mensuration method that showed the lowest SEM of angular measurements was intersegmental rotation with 0.79°. There were no SEMs reported for translational measurements. The biomechanical mensuration method that showed the highest SEM of angular measurements was the Cobb method (C2–C7) with 7.0°. The biomechanical mensuration method that showed the lowest MAD of angular measurements was intersegmental rotation with 0.35°. Intersegmental translation was the only study that reported a MAD of translation measurements with 0.345 mm.

The biomechanical mensuration method that showed the highest SEM of angular measurements was the Cobb method (C2–C7) with 7.0°. There were no SEMs reported for translational measurements. The biomechanical mensuration method that showed the highest MAD of angular measurements was the Cobb method (C2–C7) with 5.2°.

The biomechanical mensuration method that showed the greatest precision of SEMs across all studies in angular measurements was intersegmental rotation, with SEMs ranging from 0.79 to 1.805°. The Cobb method (C1–C7) was the only angular measurement method with more than one study with MADs reported, which ranged from 0.9 to 3.4°. The biomechanical mensuration method that showed the least precision of SEMs across all studies in angular measurements was the Cobb method (C2–C7), with SEMs ranging from 2.71 to 7.0°.

## 4. Discussion

The study’s hypothesis was that the available literature reporting on the intra- and inter-examiner reliability of biomechanical assessment of the sagittal cervical spine in radiography used in clinical practice would provide a sufficiently low bias risk, with high-quality studies establishing good or better reliability. In general, the results of this SROL confirm this hypothesis, and the null hypothesis is rejected. Overall, six sagittal cervical radiographic measurement methods were found to be rated ‘good-to-excellent’ for inter-examiner reliability, and five methods were found to be rated ‘good-to-excellent’ for intra-examiner reliability; meanwhile, the remaining methods were found to have ‘moderate’ examiner reliability. The advantages of this SROL design included following established frameworks, such as PRISMA, PROSPERO, and SWiM recommendations. Further, our use of the three-reviewer process, incorporating two initial assessments of data followed by the third referee review, was used to reduce bias in data collection and improve systematic review quality and credibility. It has been shown from prior studies that the use of multiple reviewers increases the quality and accuracy of data collection and synthesis in a SROL [81]. Prior studies have recommended the use of SWiM guidelines for SROL sub-group analysis to improve the confidence and certainty of conclusions and findings [82]. The timeliness of a comprehensive and well-designed SROL on the reliability of sagittal cervical measurement techniques is noted, given that prior rapid reviews, narrative reviews, and selective review studies have shown conflicting and (oftentimes erroneous) conclusions regarding the reliability of radiographic mensuration [84,85,86,87,88].

The current SROL used an end search date (most recent publication date) of 24 January 2023; thus, publications from this date to the present were not included in our analysis. However, a cursory search of the recent literature has demonstrated similar findings to our SROL findings. Specifically, recent investigations have utilized computer-assisted/machine learning programs for measurement construction, and have compared these to trained examiners while confirming excellent reliability of specific cervical spine mensuration methods [89,90]. The development of artificial intelligence (AI), particularly convolutional neural networks (CNNs), to automate the measurement of sagittal cervical spine alignment from lateral radiographs is an area that is rapidly developing [89,90]. For instance, Hosseini et al. [89] assessed intra-examiner reliability and concurrent validity of computer vision convolutional neural network (CNN) algorithms for sagittal cervical alignment measurements against expert human evaluation. The CNN showed perfect repeat reliability (ICC = 1.0, root mean square error (RMSE) = 0, R^2^ = 1), while the expert observer demonstrated excellent reliability across all variables. CNN–human construct validity was also excellent, with all 18 presenting ICCs ≥ 0.8, strong-to-excellent R^2^ values for 16 variables, and small RMSEs (0.37–2.89), supporting the CNN’s reliability and validity for sagittal cervical spine mensuration. Vogt et al. [90] evaluated pre- and postoperative lateral cervical radiographs from 129 anterior cervical surgery patients using two surgeons and an artificial intelligence (AI) algorithm based on four deep CNNs. Two trained surgeons measured C2–C7 lordosis, C1–C7 and C2–C7 sagittal vertical axes, C7 slope, and T1 slope twice, then compared the results with AI measurements. Intra-rater (0.92–1.0) and inter-rater (0.91–1.0) ICCs showed excellent human agreement, and the AI algorithm also achieved excellent ICCs preoperatively (0.80–1.0) and postoperatively (0.86–0.99). AI–human mean errors were smallest for C1–C7 SVA and largest for C2–C7 lordosis, with automated measurement successful in 99% and 98% of pre- and postoperative images, respectively, except for the T1 slope.

### 4.1. Conglomeration and Heterogeneity of Reliability Measure Findings

The explanation of heterogeneity of findings is important in this SROL. Current studies are adapting the use of machine learning and digital analysis for confirmation of reliability of cervical spine mensuration. Studies in this SROL incorporated both manual and digital measured assessments. Thus, there can be significant differences in the reported ICCs between manually assessed spine parameters and computer-aided mensuration methods. Although some previous studies have shown that manual methods provide lower reliability, there is excellent reliability with computer-aided mensuration, including studies with R^2^ = 1 (perfect correlation) models for machine learning programs [90]. Other sources of heterogeneity can often be explained by the quality and type of image used, whether digital or plain film radiographs. The source of the image can alter mensuration, including X-ray radiographs versus EOS low-dose images or biplanar stereo-radiography. The heterogeneity of findings can also be explained by differences in the statistical analysis and reporting. In the included studies, some used ICCs or Pearson correlation coefficients while others used Cohen’s kappa agreement or Bland–Altman plots, all of which can have different reliability assessment and interpretation [77].

### 4.2. Systematic Review Significance and Clinical Application

There is no available consensus in the scientific literature for the number of high-quality investigations to be able to claim that a specific radiographic measurement method is high quality with good-to-excellent examiner reliability [91,92,93]. Therefore, in the current systematic review, for a specific line drawing measurement method to be rated ‘good-to-excellent’ for both inter- and intra-examiner reliability, a minimum of two low-risk/high-quality studies that found good-to-excellent examiner reliability were needed. This approach is directly modeled on published peer-reviewed systematic reviews in closely analogous domains. Mylonas et al. [91] used an identical structure in their systematic review of non-radiographic forward head posture measurement reliability, and concluded that the strongest evidence supported photogrammetry with good-to-excellent reliability. While Mylonas et al. defined ‘strong’ as three high-quality studies, our use of ‘at least two or more’ is a conservative variant within the same best-evidence synthesis tradition (van Tulder et al. [92] designed frameworks where two consistent high-quality studies routinely support moderate-to-strong evidence for measurement properties). Furthermore, in a 2008 SROL on sagittal plane curves and health outcomes, the authors used the reference number of two or more high-quality studies to receive a strong evidence rating [93]. Thus, since no universal ‘gold standard’ minimum number of studies exists, the field relies on this structured, transparent qualitative synthesis when heterogeneity precludes meta-analysis. Our threshold is therefore conservative, places conclusions at the highest evidence level, and is fully consistent with accepted methodology in postural and musculoskeletal measurement systematic reviews.

Using these criteria, this SROL discovered good-to-excellent intra-examiner reliability for the following biomechanical mensuration methods: intersegmental rotation angles, the thoracic inlet angle, the T1 slope, the C2–C7 posterior tangent method for total cervical lordosis, and the Cobb C2–C7 method for total cervical lordosis (Figure 6). Likewise, using these criteria, we identified good-to-excellent inter-examiner reliability for the following biomechanical mensuration methods: intersegmental rotation angles, the thoracic inlet angle, the T1 slope, translation using the sagittal vertical axis of C–C7, the C2–C7 posterior tangent method for total cervical lordosis, and the Cobb C2–C7 method for total cervical lordosis (Figure 4). The remaining methods would be rated as moderate quality with moderate examiner reliability (Figure 4 and Figure 6). Each specific radiographic measurement method is individually rated for its quality and statistical findings in different investigations in Tables S2 and S3. Specifically, on pages 136–138 of Table S3, we present the sub-group analysis of intra-examiner reliability studies, and on pages 139–142 Table S4, we present the sub-group analysis of inter-examiner reliability studies.

It seems this SROL is the first in the body of knowledge to provide this complete, comprehensive analysis. The use of the preferred reporting items for systematic reviews and meta-analyses (PRISMA) checklist for this SROL improves its quality, reduces erroneous conclusions, and improves confidence in its assessment [86]. The use of sagittal cervical radiography is widespread for clinicians and physicians who treat cervical spine disorders. Sagittal cervical biomechanical measurements are used by entry-level and trauma clinicians [94], primary care providers [95], surgeons [96,97,98], physical therapists [99,100], chiropractors [100], device manufacturers [101], modeling and machine learning scientists [89,90,96,100], third-party payors [101,102], governmental bodies [103], and educational institutions [102].

Due to the significant contribution of neck pain to GBD and YLD, the reliability of diagnostic assessments is essential. Unhealthy, abnormal sagittal cervical spine biomechanics and measurements can cause and are associated with altered health and quality of life, pain, and dysfunction and disability. Recent studies on abnormal forward head posture (FHP), loss of cervical lordosis, and abnormal spine alignment have shown association with numerous disorders and abnormal health outcomes. Abnormal FHP has been associated with an altered resting brain state [104], reduced overall human performance and mental activity [104,105], neck pain [106], altered gait and postural control [107], decreased cerebral blood perfusion and hemodynamic control [108,109], postural dislocation [110], temporomandibular joint disorders [111,112], and respiratory function [112].

Physicians providing interventions to improve sagittal cervical alignment and posture rely on cervical spine radiography for the triage, diagnosis, management (treatment and referrals), prognosis, and progress evaluation of cervical spine disorders [97]. The application of these techniques requires lateral cervical radiographic mensuration to determine specific treatment strategies and monitor progress [113,114,115,116,117]. Studies have indicated the importance of cervical spine and thoracic inlet morphology to assess the relative ideal sagittal cervical spinal alignment and posture for a given patient, which can vary [118]. The morphological assessment of the cervical spine requires reliable radiographic mensuration of sagittal cervical spine parameters [118,119]. Recent advancements in artificial intelligence (AI) and machine learning technology have also improved the speed and reliability of biomechanical mensuration of the cervical spine [89,90]. It is expected that future advances in this technology will continue to assist decision-making and improve clinical certainty in the triage, diagnosis, management (treatment and referrals), prognosis, and progress evaluation of cervical spine disorders [120,121,122].

### 4.3. Previous Non-Systematic Literature Reviews

Imaging studies have been used as a high standard in the diagnosis and treatment of cervical spine disorders [123,124]. This current SROL improves the understanding of the reliability of cervical spine biomechanical mensuration and contrasts this with prior studies, finding that X-ray line drawing has no clear reliability [84,85,86,87,88]. The use of sagittal cervical radiography to produce diagnostic images can be encouraged, as the results of the biomechanical mensuration will improve patient diagnosis, treatment options, and outcomes [124]. Despite this clinical necessity for the use of spinal radiography, there have been some recent studies challenging the reliability, validity, and use of radiography for neck pain and other spinal musculoskeletal conditions [7,84,85,86,87,88,125]. For example, regarding radiographic measurements of cervical lordosis (Figure 3) that have been reported, there is often conflicting validity among the evidence reported in the scientific literature [125,126], and one could argue that some of these discrepancies may be due to a lack of reliability with these measurements.

In their 2018 systematic review and meta-analysis, Guo et al. [125] reported that there was no difference in radiographically measured cervical lordotic values between patients with chronic neck pain compared to asymptomatic controls. Problematically, Guo et al. [125] cited and included Harrison et al.’s 1996 [127] investigation as a case–control when in fact this manuscript did not report such data, and they [125] did not identify Harrison et al.’s 2004 [128] investigation as a case–control to be included in their [115] analysis instead. This error may have led to an erroneous conclusion [115] in that radiographic cervical lordosis is not a significant predictor of neck pain. For example, the 2024 systematic review and meta-analysis by Kim et al. [126] incorporated the 2004 Harrison et al. [128] case–control investigation in their analysis, and concluded that patients with neck pain have a statistically and clinically significant reduced cervical lordosis on X-ray. Similarly, the recent (2026) meta-analysis by Ferraz et al. [129] found that sagittal cervical alignment has prognostic value. Within twenty included clinical publications, a C2–C7 sagittal translation > 40 mm and a T1 slope minus a cervical lordosis mismatch greater than 15–20° consistently predicted disability levels across three validated questionnaires (NDI, EQ-5D, and SRS-22). Thus, while there exists evidence to the contrary [125], there is strong evidence supporting the use of sagittal spinal radiography to analyze, diagnose, and treat patients with cervical spine disorders [126,128,129,130,131,132]. The results of our current SROL establish the reliability of the various X-ray measurements for sagittal cervical spine displacements, and provides the foundation for continued investigations into the validity of cervical deformity evaluations from a displacement perspective.

### 4.4. Strength, Limitations, and Future Research Recommendations

This SROL followed the PRISMA study design and checklists for reporting of systematic reviews and meta-analyses. This SROL was registered with the international prospective register of systematic reviews (PROSPERO) prior to any article search. This SROL performed an extensive literature search from numerous health and biotechnology article databases. The thorough and multi-layered search strategy yielded 51 articles that met the inclusion and exclusion criteria. This large number of studies is rare for most SROLs. An additional strength of the SROL was the use of the synthesis without meta-analysis (SWiM) guidelines for the presentation of sub-group assessments for intra- and inter-examiner reliability. Additionally, the use of two separate reviewers and a third referee reviewer for consensus was an important feature of our design. The use of the QAREL tool for risk of bias and quality assessment within the SROL is also important. Future SROLs should include a similar multi-layer methodology to ensure quality of conclusions.

The restricted inclusion of articles to only those in the English language might be thought to increase the risk of bias in systematic reviews. However, Dobrescu and colleagues [133] identified that language restriction to English articles had no effect on the outcomes of systematic reviews, and given our inclusion of 51 unique articles on this topic, the influence of language restriction is likely diminished. That said, there are several biomechanical mensuration methods included, which diminishes the number of investigations per method. Many studies did not document or calculate SEM and MAD for their methods of measurement, and the included studies used different statistical analyses to establish reliability. Also, given the absence of meta-analyses on the topic of reliability of radiographic mensuration, the investigators could not perform an analysis of possible crossover between upright standing lateral cervical radiographs and other cervical spine imaging. That said, reliability was nonetheless evaluated in the non-crossover studies [134]. No formal meta-analysis was performed in this study either, which would be a good follow-up to perform from the included studies of this SROL.

Other limitations to be considered are those in the studies that meet the inclusion criteria. Reliability studies need to make sure they have calculated a power analysis to ensure sufficient power of their results and that they are answering all questions of the QAREL checklist (or any other reliability study checklist) to ensure the highest quality and lowest risk of bias (Figure 1, Table 2). Not all studies included met these criteria. Three out of 11 questions (questions 5–7) on the QAREL instrument had less than a 30% yes response. It is not known whether this finding represents a systemic problem with reliability studies in general or if this is just something reports generally do not find relevant. Since the QAREL instrument uses the total score as the standard for rating studies, it is not known how weighting differences in these three questions might influence the specific overall study rankings. Still, our analysis and the QAREL instrument provide a clearly delineated guide to rate the quality of an article and should be applied to future research.

Future reliability studies could incorporate AI as a tool to improve conclusions. An automated AI-based algorithm could advance the field addressed in future SROLs by detecting key cervical landmarks (C2, C7, T1), automate drawing mensuration lines, and calculate multiple parameters (the Harrison posterior tangent method, Cobb angles, sagittal vertical axis, T1 slope, and thoracic inlet angle) [89]. This would enhance objectivity by eliminating inter- and intra-examiner variability inherent in manual mensuration, and would dramatically reduce manual analysis time from several minutes per radiograph to a few seconds. Such automation could facilitate large-scale radiographic screening, simplify longitudinal analysis of sagittal alignment changes, and, ultimately, improve the efficiency and consistency in the triage, diagnosis, and management of cervical spine disorders.

It is recommended that future reliability studies on biomechanical mensuration methods need to include an evaluation of both inter- and intra-examiner reliability, using a minimum of two examiners for each and a minimum of 30 unique patient radiographs. The use of ICCs, SEMs, and MADs at a minimum is recommended to obtain proper study results and to facilitate further meta-analysis. Lastly, researchers should ensure that each item on the 11-item QAREL instrument is adequately reported in their manuscript. Researchers should continue to perform SROLs and meta-analyses on the reliability of biomechanical mensuration methods in all global and regional sagittal and anteroposterior spinal imaging. An essential element of the validity of measurements is that the measurement methods are reliable.

## 5. Conclusions

This SROL discovered good-to-excellent intra-examiner reliability for the following biomechanical mensuration methods: intersegmental rotation angles, the thoracic inlet angle, the T1 slope, the C2–C7 posterior tangent method for total cervical lordosis, and the Cobb C2–C7 method for total cervical lordosis. Likewise, using these criteria, we identified good-to-excellent inter-examiner reliability for the following biomechanical mensuration methods: intersegmental rotation angles, the thoracic inlet angle, the T1 slope, translation using the sagittal vertical axis of C–C7, the C2–C7 posterior tangent method for total cervical lordosis, and the Cobb C2–C7 method for total cervical lordosis. This SROL demonstrated that the use of sagittal cervical spine radiography is a reliable tool for the assessment of the sagittal cervical spine.

## Figures and Tables

**Table 1:** QAREL instrument results for sagittal cervical radiographic biomechanical mensuration intra- and inter-examiner reliability studies [26,27,28,29,30,31,32,33,34,35,36,37,38,39,40,41,42,43,44,45,46,47,48,49,50,51,52,53,54,55,56,57,58,59,60,61,62,63,64,65,66,67,68,69,70,71,72,73,74,75,76].

Study	QAREL Instrument Questions											Sum	Bias Risk	Quality	Reliability
	1	2	3	4	5	6	7	8	9	10	11				
*Alhilali 2013 [26]*	Y	Y	Y	Y	Y	Y	Y	Y	U	U	Y	9	L	H	IR
*Andriola 2018 [27]*	Y	Y	Y	U	U	Y	Y	Y	U	Y	Y	8	L	H	IA
*Balouch 2020 [28]*	Y	Y	U	U	U	U	U	U	Y	Y	Y	5	M	M	B
*Barth 2017 [29]*	Y	Y	U	U	U	U	U	U	Y	Y	Y	5	M	M	IA
*Bellabarba 2021 [30]*	Y	Y	U	U	U	U	U	U	NA	Y	Y	4	H	L	IR
*Bono 2011 [31]*	Y	Y	U	U	U	U	U	Y	U	Y	Y	5	M	M	IR
*Cannada 2003 [32]*	Y	Y	U	NA	U	Y	Y	U	NA	Y	Y	6	M	M	IR
*Chamnan 2020 [33]*	Y	Y	U	U	U	U	U	U	Y	Y	Y	5	M	M	B
*Champain 2006 [34]*	Y	Y	U	U	U	U	U	U	Y	Y	Y	5	M	M	B
*Chen 2012 [35]*	Y	Y	U	U	U	U	U	Y	Y	Y	Y	6	M	M	B
*Cote 1997 [36]*	Y	Y	Y	U	U	Y	Y	U	U	Y	Y	7	M	M	IR
*Daffin 2019 [37]*	Y	Y	U	U	U	U	U	U	Y	Y	Y	5	M	M	IA
*Donk 2017 [38]*	Y	Y	U	U	U	U	U	U	U	U	U	2	H	L	IR
*Douglas 2007 [39]*	Y	Y	U	U	U	U	U	U	Y	Y	Y	5	M	M	B
*Dvorak 1988 [40]*	Y	Y	Y	U	U	U	U	U	NA	Y	Y	5	M	M	IR
*Frobin 2002 [41]*	Y	Y	U	U	U	U	U	U	Y	Y	Y	5	M	M	B
*Gadotti 2013a [42]*	Y	Y	U	U	U	Y	Y	U	Y	Y	Y	7	M	M	IA
*Gadotti 2013b [43]*	Y	Y	U	U	U	Y	Y	U	Y	Y	Y	7	M	M	B
*Guyard 2021 [44]*	Y	Y	U	U	U	U	U	U	Y	Y	Y	5	M	M	B
*Gwinn 2009 [45]*	Y	Y	U	U	U	U	U	U	Y	Y	Y	5	M	M	B
*Hardacker 1997 [46]*	Y	U	U	U	U	U	U	U	NA	Y	Y	3	H	L	B
*Harrison 2000 [47]*	Y	Y	Y	Y	U	U	U	Y	Y	Y	Y	8	L	H	B
*Herman 2004 [48]*	Y	Y	Y	Y	U	U	U	Y	Y	Y	Y	8	L	H	B
*Jackson 1993 [49]*	Y	Y	Y	Y	U	Y	Y	Y	Y	Y	Y	10	L	H	B
*Janusz 2016 [50]*	Y	Y	Y	Y	U	U	U	Y	Y	Y	Y	8	L	H	B
*Jiang 2014 [51]*	Y	Y	Y	Y	U	U	U	Y	Y	Y	Y	8	L	H	B
*Johnson 1998 [52]*	Y	Y	NA	U	U	U	U	Y	Y	Y	Y	6	M	M	IA
*Kuhr 1996 [53]*	Y	Y	U	U	U	N	N	U	Y	Y	Y	5	M	M	B
*Kunakornsawat 2017 [54]*	Y	Y	U	U	U	U	U	U	U	Y	Y	4	H	L	B
*Lafage 2015 [55]*	Y	Y	U	U	U	U	U	U	Y	Y	Y	5	M	M	B
*Lee 2017 [56]*	Y	Y	Y	Y	U	U	U	Y	Y	Y	Y	8	L	H	B
*Li 2009 [57]*	Y	Y	Y	Y	U	Y	Y	Y	Y	Y	Y	10	L	H	B
*Lopez 2021 [58]*	Y	Y	U	NA	U	U	U	U	NA	Y	Y	4	H	L	IR
*Manabe 2022 [59]*	Y	Y	U	U	U	U	U	U	Y	Y	Y	5	M	M	B
*Marshall 1996 [60]*	Y	Y	N	NA	U	U	U	Y	Y	Y	Y	6	M	M	IR
*Nakamura 2014 [61]*	Y	Y	U	U	U	U	U	U	Y	Y	Y	5	M	M	B
*Paholpak 2018 [62]*	Y	Y	U	U	U	U	U	U	U	Y	Y	4	H	L	B
*Phillips 1999 [63]*	Y	Y	Y	Y	Y	Y	Y	U	U	Y	Y	9	L	H	IR
*Plaugher 1990 [64]*	Y	Y	U	U	U	N	U	U	U	Y	Y	4	H	L	B
*Rochester 1994 [65]*	Y	Y	Y	Y	Y	Y	U	Y	Y	Y	Y	10	L	H	B
*Salameh 2021 [66]*	Y	Y	U	U	U	U	U	U	U	Y	Y	4	H	L	B
*Shin 2016 [67]*	Y	Y	U	U	U	U	U	U	Y	Y	Y	5	M	M	B
*Shoda 2004 [68]*	Y	Y	Y	Y	U	U	U	U	Y	Y	Y	7	M	M	B
*Sigler 1985 [69]*	Y	Y	Y	Y	U	Y	U	Y	Y	Y	Y	9	L	H	B
*Silber 2004 [70]*	Y	Y	Y	Y	U	U	U	Y	Y	Y	Y	8	L	H	B
*Svedmark 2011 [71]*	Y	Y	U	U	U	U	U	U	U	Y	Y	4	H	L	IA
*Tang 2020 [72]*	Y	Y	Y	Y	U	U	U	U	Y	Y	Y	7	M	M	B
*Vidal 2013 [73]*	Y	Y	U	U	U	U	U	U	Y	Y	Y	5	M	M	B
*Wellborn 2000 [74]*	Y	Y	Y	Y	U	U	U	Y	Y	Y	Y	8	L	H	B
*Wu 2015 [75]*	Y	Y	Y	Y	U	U	U	U	Y	Y	Y	7	M	M	B
*Zhang 2022 [76]*	Y	Y	U	U	U	Y	Y	Y	Y	Y	Y	8	L	H	B

**Index:** Y = yes; N = no; U = unclear; NA = not applicable; L = low; M = moderate; H = high; IA = intra-examiner; IR = inter-examiner; B = both (intra- and inter-examiner).

**Table 2:** Answers to QAREL instrument questions per each question for intra- and inter-examiner reliability studies for sagittal cervical radiographic biomechanical mensuration methods, as well as study quality and reliability type.

QAREL Answers	QAREL Instrument Questions											Study Quality		Study Reliability	
	1	2	3	4	5	6	7	8	9	10	11				
*Yes*	51	50	19	16	3	12	10	18	35	49	50	*L*	9	*IA*	6
*No*	0	0	1	0	0	2	1	0	0	0	0	*M*	27	*IR*	10
*Unknown*	0	1	30	32	48	37	40	33	11	2	1	*H*	15	*B*	35
*Not Applicable*	0	0	1	3	0	0	0	0	5	0	0				

**Index**: L = low; M = moderate; H = high; IA = intra-examiner; IR = inter-examiner; B = both (intra- and inter-examiner).

## Data Availability

All data are available in the manuscript and Supplementary Materials.

## Supplementary Materials

The following supporting information can be downloaded at: https://www.mdpi.com/article/10.3390/jcm15166146/s1, Table S1. Prisma 2020 checklist; Table S2. Study characteristics and findings of the reliability studies on biomechanical mensuration methods of sagittal cervical radiography used in clinical practice; Table S3. Sub-group analysis of intra-examiner reliability studies on biomechanical mensuration methods of sagittal cervical radiography by intra-examiner reliability statistics, study quality, SEM, and MAD; Table S4. Sub-group analysis of inter-examiner reliability studies on biomechanical mensuration methods of sagittal cervical radiography, by inter-examiner reliability statistics, study quality, SEM, and MAD.

## Abbreviations

AI	Artificial Intelligence
ARA	Absolute Rotation Angle (e.g., ARA C2–C7)
CBP	Chiropractic BioPhysics
CINAHL	Cumulative Index to Nursing and Allied Health Literature
CRD	Centre for Reviews and Dissemination (part of PROSPERO ID: CRD42023402990)
EOS	EOS Imaging System (low-dose biplanar stereo-radiography)
EQ-5D	EuroQoL 5-Dimension (health-related quality-of-life questionnaire)
FHP	Forward Head Posture
GBD	Global Burden of Disease
ICC	Intraclass Correlation Coefficient
MAD	Mean Absolute Deviation (or Distance)
MeSH	Medical Subject Headings
NDI	Neck Disability Index
NP	Neck Pain
PICO	Patient/Population, Intervention, Comparison, and Outcomes
PRISMA	Preferred Reporting Items for Systematic Reviews and Meta-Analyses
PROSPERO	International Prospective Register of Systematic Reviews
PRESS	Peer Review of Electronic Search Strategies
QAREL	Quality Appraisal Tool for Studies of Diagnostic Reliability
QOL	Quality of Life
RRA	Relative Rotational Angle (intersegmental)
SEM	Standard Error of Measurement
SROL	Systematic Review of the Literature
SRS-22	Scoliosis Research Society-22 (questionnaire)
SVA	Sagittal Vertical Axis (e.g., SVA C2–C7)
SWiM	Synthesis Without Meta-Analysis
YLD	Years Lived with Disability
