# Supplementary material for: The Reliability of Biomechanical Mensuration Methods of the Sagittal Cervical Spine in Radiography Used in Clinical Practice: A Systematic Review of the Literature
Source: J Clin Med. 2026 Aug 7;15(16):6146. doi: 10.3390/jcm15166146 (PMC13513229; doi:10.3390/jcm15166146)
Supplement: Supplementary file 1 [file jcm-15-06146-s001.zip › jcm-4265226-Tables S2-S4.pdf]

Table S2. Study Characteristics and Findings of the Reliability Studies on Biomechanical Mensuration Methods of Sagittal Cervical Radiography Used in Clinical Practice

Author and Year	Sample #, Age, Sex	# Examiners	Repeat Analysis Details	Type of Imaging	Method of Analysis	Inter-Examiner Reliability	Intra-Examiner Reliability	SEM MAD
Alhilali 2013	Trauma Patients: 103 (77 male, 26 female), mean age 56y, age range (17-93); Control Patients: 104 (73 male, 31 female), mean age 52y, age range (17-93); Total Patients: 207 (150 male, 57 female), mean age 54y, age range (17-93)	2	Reproducibility of the IDA was assessed by having 2 neuroradiologists independently measure the IDA for all disk spaces from C2–3 to C7–T1 in 17 randomly selected patients from both control and trauma groups (102 total disk spaces), with 10 of the tested disk spaces demonstrating ADL disruption on MR imaging. Radiologists were blinded to both each other's	CT examinations were performed on 16- or 64-MDCT scanners (LightSpeed VCT; GE Healthcare, Milwaukee, Wisconsin). CT acquisitions were obtained from the infraorbital rim to the level of T1–T2 by use of the axial technique, 0.5 pitch, 1.2-mm collimation, 350 maximal mA, 120 kept, and 18-cm FOV, in bone and standard algorithms, with 2.5-mm sagittal and coronal	For measurement of the IDA, 3 points are first identified: 1) the first point at the midpoint of the disk space at its most posterior margin, 2) a second point at the anterior aspect of the endplate of the upper vertebral body, and 3) a third point at the anterior aspect of the endplate of the lower vertebral body. A line is drawn between the first and second points and the first and	The Lin correlation coefficient (c) for IDA measurements on test disk spaces, used to assess IDA reproducibility, was 0.9576 (95% CI, 0.9417–0.9692), indicating substantial agreement among observers.		

			measurements and MR imaging findings. IDAs were then measured in all patients and control participants, in random order, at all levels from C2–3 to C7–T1 by 1 of the 2 neuroradiologists blinded to the clinical history and MR imaging findings. At our institution, cervical spine CT examinations are interpreted by fellowship-trained neuroradiologists and musculoskeletal radiologists.	reconstructions. Patients were immobilized in a cervical collar during CT image acquisition.	third points. The IDA is defined as the angle formed between these 2 lines. IDA was measured on midline sagittal CT images by use of the angle measurement tool on our PACS. If there were anterior osteophytes, the angle excluded the osteophytes. IDA was measured for all disk spaces from C2–3 to C7–T1 in both patients with ADL disruption and in control participants. Evaluation of subjective disk widening was performed with use of the			

					prospective interpretation of the CT study by the initial interpreting radiologist. A true positive finding for subjective disk widening was considered an initial interpretation of the CT scan, suggesting disk space widening at a level of injury seen on MR imaging.			

Andriola 2018	Twenty-five patients with indication for bimaxillary orthognathic surgery to correct skeletal class III dentofacial deformities (10 men, 15 women; mean age, 29.28 8.23 years; range 18–48 years).	1	The reproducibility of the radiographer's technique of taking radiographs in NHP was investigated using a photographic method previously reported by Bister et al and used by Saujani et al, and it was found to be acceptable.<br>The patients' data were blinded before the cephalometric analysis was carried out. The lateral cephalographs were traced and landmarks were identified by 1 operator (FOA) in SCANORA Software	In order to capture all structures from the Nasion-Sella line (NSL) to the seventh cervical vertebra (CV7) and minimize external influence, the lateral cephalograph acquisition technique was slightly modified: no ear rods were used so the field of view (FOV) could be moved down. Instead, the ears rods were adjusted to patients' necks to help them keeping immobile.<br>Modified lateral cephalographs taken in NHP, with the subject standing barefoot	Changes in Cervical Lordosis, Cranio-Cervical Angle, Cranio-Vertical Angle, Mandibular Angle and Occlusal Plane Before (T1) and 6 Months (T2) After Orthognathic Surgery<br>CV1/CV2, angle between CV1 and CV2.<br>CV1/CV7, angle between CV1 and CV7.<br>CV3/CV7, angle between CV3 and CV7.<br>NLS/Go-Gn, angle between Nasion Sella Line and mandibular plane.<br>NLS/VER, angle between Nasion		The repeatability coefficients for the V-Line (facial plane, from soft tissue Nasion to soft tissue Sub-nasale) and the E-plane (from soft tissue Tip of Nose to soft tissue Menton) were 4.02 and 4.12, respectively.<br>The ICCs for all variables ranged between 0.996 and 1.000, demonstrating a high reliability of the measuring method.<br>Intraclass correlation coefficients (ICCs) were calculated to estimate the repeatability of	

			(SOREDEX, Tuusula, Finland). All measurements for both T1 and T2 were repeated 3 times by the same investigator at 2-week intervals and the average values of the 3 measurements were calculated to be used in the statistical analysis.	in orthoposition (considered the most reproducible natural standing body position and defined as the intentional position from standing to walking) which was obtained by letting the patient walk slightly on the spot. Then, patients were asked to stand at rest in a relaxed manner. To improve reproducibility the subjects were asked to tilt the head forward and backward with decreasing amplitude until it was that the natural head balance was reached (self-	Sella Line and true vertical line. NSL/OCL, angle between Nasion Sella Line and occlusal plane. NSL/OPT, angle between Nasion Sella Line and odontoid process of CV2 tangent.		the measures performed by the investigator in the 3 different intervals. Descriptive statistics were obtained, including the means and standard deviations at T1 and T2.	

				balance position). After self-balance position was obtained, patients should look straight into their own eyes in a mirror, which was mounted on a wall in front of the X-Ray Unit. All subjects were asked not to swallow or move their heads and tongues, keeping their jaws at maximum intercuspation with lips and tongue in a resting position while the radiograph was being acquired. Special care was taken to ensure that the head was not moved during the examination.				

				The above procedure was repeated if the patient's position changed during the image acquisition. Patients higher than 1.90 m were excluded once they were too tall to stand in orthoposition under the Cephalometric X-ray Unit.				

Balouch 2020	A total of 143 patients met inclusion criteria for the study. The mean age of patients was 52 ± 11.5 years and 42% (60/143) of the group were female. Seventy-three patients had one-level fusions, 41 patients had two-level fusions, and 29 patients had three-level fusions for a collective total of 242 fused levels. Interbody types included 81 structural allograft spacers with	Five observers (three spine surgeons and two clinical researchers)	Inter-reliability and intraobserver reliability measurements of both angular and linear methods were performed by five observers (three spine surgeons and two clinical researchers) on patients with postoperative CTs. Each observer measured each patient twice with a minimum of 7 days between measurements of the same patient. Interobserver and intraobserver reliability measurements were assessed for both angular and linear methods using interclass	Standardized, calibrated lateral, and flexion-extension radiographic imaging with clearly visible vertebral bodies and fusion structures. Patients with cervical computed tomography (CT) imaging at least 1-year after surgery were marked separately to confirm pseudarthrosis.	Using flexion-extension dynamic radiographs, interspinous motion (ISM) at each fused level was measured with the novel angular method between spinous processes using three distinct landmarks: the dorsal points of consecutive spinous processes and the midpoint of the screw head between the spinous processes. The angles were defined by lines from screw heads to dorsal points of spinous processes at 150% magnification.	Intraobserver and interobserver reliability measurements were performed by each observer on standardized flexion-extension radiographs of each patient with CT using the angular and linear methods. Both methods had high intra-observer and interobserver reliability, with interclass correlation coefficients of 0.974 for the linear method and 0.986 for the angular method (both P <0.001). The intrarater reliability for	Intraobserver and interobserver reliability measurements were performed by each observer on standardized flexion-extension radiographs of each patient with CT using the angular and linear methods. Both methods had high intra-observer and interobserver reliability, with interclass correlation coefficients of 0.974 for the linear method and 0.986 for the angular method (both P <0.001). The intrarater reliability for	

	plate, 84 PEEK cages with plate, 40 titanium cages with plate. In this study, all patients with standalone cages had only one-level fusions.		correlation coefficient (ICC) and intra-rater reliability, ranging from a value of 0 to 1, with 1 representing complete reliability of measurements, and 0 representing no reliability.		The landmarks used in this method were clearly visible on both flexion and extension radiographs, where the angles drawn between them were replicable at least two consecutive times by each observer. The procedure for determining cutoff for possible pseudarthrosis is described in the following section. The previously published linear method of detecting pseudarthrosis described by Riew et al was also used on the same dynamic	angular measures were 0.975, 0.974, 0.980, 0.986, and 0.957 (mean = 0.974) and for linear measures were 0.973, 0.971, 0.967, 0.953, and 0.904 (mean =0.954) ($P < 0.001$ for all).	angular measures were 0.975, 0.974, 0.980, 0.986, and 0.957 (mean = 0.974) and for linear measures were 0.973, 0.971, 0.967, 0.953, and 0.904 (mean =0.954) ($P < 0.001$ for all).	

					radiographs at 150% magnification to measure the distance between spinous processes to the closest tenth of a millimeter. ISM <1 mm between flexion and extension radiographs was defined as possible pseudarthrosis.			

Barth 2017	282 radiographs of 35 patients (16 male, 19 female) with a mean age of 53.1 ± 12.0 years.	1		neutral lateral, flexion and extension lateral radiographs, and a cervical computed tomography (CT) scan.	Based on manual point definitions in lateral radiographs, the following values were calculated by the software: vertebral wedging angles C3–C7; segmental height by measuring the distance between the center points of C3/4, C4/5, C5/6, and C6/7; angle between the caudal and cranial endplates of C2 and C7 in neutral position, and range of motion (ROM) C2/C7 from flexion/extension lateral radiographs. ROM of C3/4, C4/5, C5/6, and C6/7 was		For analysis of measurement precision, intraclass correlation coefficients (ICC) were calculated (1) between the wedging angles of identical vertebrae and (2) between differences in height measurements of identical segments. All timepoints were included, and operated segments were excluded from analysis. ICC values close to 1 represent perfect agreement between measurements; values close to 0	

					determined using the angles between cranial and caudal endplates in flexion/extension lateral radiographs.		indicate corrupt measurements. The precision of the software's prognosticated values with regard to the de facto measured postoperative segmental height and ROM was investigated using the ICC. For analysis of the measurement reliability, the ICC between the wedging angles and segmental height of identical vertebrae in all available lateral radiographs was calculated. As measurements in postoperative segments may hamper accuracy, these	

							segments were excluded from analysis. The ICC for wedging angles throughout indicated low reliability of measurements. The highest ICC (0.578) could be achieved in C4. In the segments C5–C7, the ICC was even lower (0.201 in C7). In contrast, the ICC for segmental height showed good reliability and ranged from 0.78 in C4/C5 to 0.89 in C3/4.	

Bellebarba 2021	100 individuals without identifiable pathology (57 male, 43 female) with a mean age of 39 years (range 19-64 years)	3 Examiners. A board-certified orthopedic spine surgeon, an orthopedic spine surgery fellow, and a second year orthopedic surgery resident (R2), attempted to measure five specific angles from each radiograph.	The three reviewers recorded their results on datasheets. Extrapolated values from these data included the average of the anterior/posterior mandible (MA) relative to the C2 anterior/posterior body (AB/MA and PB/MA).	Neutral, lateral, cervical spine upright radiographs.	The five angles measured consisted of: 1) anterior C2 body/anterior mandible angle (AB/AM), 2) anterior C2 body/posterior mandible angle (AB/PM), 3) posterior C2 body/anterior mandible angle (PB/AM), 4) posterior C2 body/posterior mandible angle (PB/PM), and 5) Occiput-C2 Angle (O-C2A). The three reviewers recorded their results on datasheets. Extrapolated values from these data included the average of the	Intraclass correlation coefficients (ICC) were calculated for each of the angular measurements. ICC data were used to assess the consistency of measurements made by multiple reviewers, measuring the same quantity. In our study, ICC data were taken on both, single measurements as well as average measurements. Additionally, Cronbach's alpha score was also evaluated to provide an understanding of		

					anterior/posterior mandible (MA) relative to the C2 anterior/posterior body (AB/MA and PB/MA).	internal consistency. Intraclass correlation coefficient data were calculated for both, single and average measures, and indicated adequate reliability across the board. The ICC score for single angle measures of O-C2A, AB/AM, AB/PM, PB/AM, and PB/PM were 0.728, 0.564, 0.499, 0.670, and 0.702 respectively. Average measures were more reliable than single measures. For average		

						measures, calculated ICC data was rated as excellent as it was between the 0.75 and 1.00 range in terms of agreement. The ICC for average measures of O-C2A, AB/AM, AB/PM, PB/AM, and PB/PM were 0.889, 0.795, 0.750, 0.859, and 0.876 respectively. Additionally, Cronbach's alpha, another statistical reliability score, demonstrated good results for O-C2A, AB/AM, PB/AM, and PB/PM and acceptable		

						results for AB/PM.		

Bono 2011	30 patients	4	Images were evaluated by four spine surgeons experienced in the care of cervical trauma. The sequence of the cases was random in regards to injury type (e.g., facet dislocation was not grouped consecutively), however, each rater reviewed the cases in the same order. De-identified plain radiographic and CT images of 30 patients who had sustained a broad spectrum of subaxial cervical spine injuries were obtained. Plain radiographs included a lateral cervical view. CT images included	Plain radiographs included a lateral cervical view. CT images included two-dimensional sagittal and paramedian reformations.	Raters were asked to measure kyphosis, translation, vertebral body height loss, and facet joint apposition (expressed as a percentage of the intact articular surface apposed) when appropriate. Kyphosis was assessed using the posterior vertebral body tangent method (Figure 7) and the endplate method. All measurements were made using plain films and CT scans with the exception of facet joint apposition, which was examined using CT images only.	Interobserver reliability was assessed using intra-class correlation coefficients (ICC) and Pearson correlation coefficient (r). ICC scores were interpreted based on the work of Shrout and Fleiss 7 with a score of 0 to 0.4 indicating poor reliability, 0.4 to 0.75 indicating moderate reliability, and greater than 0.75 indicating excellent reliability. Pearson correlation coefficient was used to		

			two-dimensional sagittal and paramedian reformations. Each image included a uniform calibration scale.		To enable linear measurement comparison between CT images and plain radiographs, the anteroposterior diameter of the uninjured superior vertebral endplate of C3 was measured as an internal calibration control.	determine how strong a correlation exists between raters with values greater than 0.50 representing substantial reliability and values less than 0.50 representing poor reliability. Substantiating the reliability of the imaging software and the raters' ability to make measurements, the C3 superior endplate diameter (control) measurement had near perfect reproducibility and correlation (ICC = 0.91, r =		

						0.92). The most reproducible injury parameter was vertebral body translation, which demonstrated excellent reliability on both CT and plain film (ICC = 0.78 and 0.87, respectively). Similarly, the r for translation measurements was substantial at 0.87 for CT images and 0.8 for plain radiographs. Measurement of kyphosis was less reproducible. The endplate method of measuring kyphosis was more		

						reproducible than the posterior tangent method. The posterior tangent method using plain radiographs (ICC = 0.2) was the least reproducible kyphosis measurement. Furthermore, it was inferior to the same method using CT. Notably, there was minimal difference between endplate and posterior tangent methods using CT (ICC = 0.55 and 0.50, respectively). Vertebral body height measurement		

						was performed separately for the anterior and posterior aspects. Plain radiographic measurement of anterior vertebral body height loss had moderate reliability (ICC = 0.47). All other vertebral body height loss measurements showed poor reliability. Measurement of facet joint apposition had poor reliability (ICC = 0.33, r = 0.39).		

Cannada 2003	27 patients (17 women and 10 men) with 29 operative levels in this study of 48 years (range, 31–69 years). Of these 29 operative levels, 18 were considered to have a solid fusion and 11 a pseudarthrosis.	3	Measurements were obtained independently by three physicians: a full-time spine attending physician, a spine fellow and a fourth-year orthopedic resident. A number 1 fine-tip lead pencil and the same goniometer were used for all the patients. Before the measurements were taken, the operative disc spaces were covered with duct tape to prevent visualization of the disc space or spaces, which would obviously bias the measurement.	Lateral flexion and extension radiographs.	Lateral flexion and extension radiographs were obtained from all the patients. Two measurements were obtained from these radiographs: Cobb angle and distance between the tips of the spinous processes of the surgically managed levels.	Statistical analysis compared interobserver reliability and the Pearson correlations between each of the two measures and the diagnosis of pseudarthrosis. The reliability among the observers for each of the two methods was measured using Cronbach's alpha test. The interobserver reliability as measured by Cronbach's alpha was 0.95 for the spinous process measurement method and 0.74		

						for the Cobb angle method. The Pearson correlation between pseudarthrosis and 2 mm or more of motion using the spinous process method was 0.77 (P < 0.001). The Pearson correlation between pseudarthrosis and the Cobb angle measurement was 0.28 (P > 0.10).		
Chamnan 2020	70 patients (37 men and 33 women) with an average age of 45.4 years (range 23–67 years) were included in the study.	2	Inter-observer measurement was done by two spine surgeons who measured independently dynamic plain X-ray. Intra-observer	The lateral dynamic plain radiographs were taken in neutral, flexion and extension positions.	Twelve occipitocervical parameters were measured on dynamic radiograph in all positions (neutral, flexion and extension):	The intra-class correlation (ICC) of intra- and inter-observer between two spine surgeons showed good to excellent	The intra-class correlation (ICC) of intra- and inter-observer between two spine surgeons showed good to excellent	

			measurement was performed for dynamic radiograph with 1 month interval between two measurements.		Posterior Atlanto-Dental Interval (PADI), Anterior Atlanto-Dental Interval (AADI), Dens-to-McRae distance, Dens-to-McGregor distance, Occipito-atlantal Cobb angle (C01 Cobb angle), Occipito-axis Cobb angle (C02 Cobb angle), Atlas-axis Cobb angle (C12 Cobb angle), Redlund-Johnell distance, Modified Ranawat distance, Clivus canal angle, Occiput inclination and Occiput cervical distance.	reliability in almost all measured parameters; the ICCs were 0.67–0.98.	reliability in almost all measured parameters; the ICCs were 0.67–0.98.	

Champain 2006	60 healthy subjects, available from previous retrospective studies, 42% men and 58% women, mean age 43 years (range 22–68 years).	2	Two observers analyzed 60 plain radiographs (30 sagittal and 30 frontal). A Wilcoxon test was performed to determine the existence of a statistically significant difference between the values obtained by the two observers. Then we calculated the inter-observer reproducibility coefficient (1.96· SD of the inter-observer errors).	Plain radiographs (sagittal and frontal).	Angular parameters: intervertebral angles, global spine inclination, sagittal curvatures, sagittal tilt, frontal curvatures (Cobb angles), vertebral wedging, pelvic postural and morphologic parameters. Linear parameters: vertebral and discal heights, sagittal vertebral axis (SVA), plumblines, pelvic postural and morphologic parameters. Indexes: overhang, listhesis, disc/vertebra height ratio.	Cervical dynamic module: we found no statistically significant differences between the ROM calculated at different vertebral levels (variance ranging from C3C4 to C6C7 between 0.6 and 1.1), except for C7T1 level. Therefore, the global reproducibility error (for a 95% confidence interval) was estimated to ±2.2 degrees. For the C6–C7 to C3–C4 levels and to ±3.4 degrees for the C7T1 level.	Cervical dynamic module: we found no statistically significant differences between the ROM calculated at different vertebral levels (variance ranging from C3C4 to C6C7 between 0.6 and 1.1), except for C7T1 level. Therefore, the global reproducibility error (for a 95% confidence interval) was estimated to ±2.2 degrees. For the C6–C7 to C3–C4 levels and to ±3.4 degrees for the C7T1 level.	

					Parameters related to vertebral kinematics on dynamic X-ray films: range of motion (ROM), vertebral translations, mean centres of rotation (MCR). The MCR is calculated only for ROM values superior to 3 degrees. Evaluation of the reliability: Precision tests were performed for dynamic modules using reference measurements on in vitro specimens. Reproducibility tests using in vivo radiological data were done	Mean reproducibility errors for the length parameters are ±1.4 mm for the vertebral translations and ±1 mm for the vertebral and discal height. As a complementary test, the intra-class correlation coefficient CCI for the calculated parameters were evaluated. A good correlation was found in within subject repetitive measures (CCI= 0.99–1.00) and good agreement between observers (CCI= 0.986–0.999).	Mean reproducibility errors for the length parameters are ±1.4 mm for the vertebral translations and ±1 mm for the vertebral and discal height. As a complementary test, the intra-class correlation coefficient CCI for the calculated parameters were evaluated. A good correlation was found in within subject repetitive measures (CCI= 0.99–1.00) and good agreement between observers (CCI= 0.986–0.999).	

					both for dynamic and postural modules.			

Chen 2012	120 adolescents. The average age was 13 years 6 months (range, 9.1-17.2 years). Males and females were equally distributed in the sample.	The OE group consisted of three orthodontic experts with more than 10 years orthodontic specialty practice. The ON group consisted of three orthodontic graduate students. The CACVL group consisted of three orthodontic graduate students who mastered the use of CACVL system.	Twenty-seven landmarks from the second to fifth cervical vertebrae on the lateral cephalogram. These landmarks were identified by three groups for three times on different occasions, respectively. Three groups were set up, including the CACVL group, the orthodontic experts (OE) group and the orthodontic novices (ON) group. The OE and ON groups did the work with complete manual identification method on the computer screen of digital	Lateral cephalogram	Twenty-seven landmarks from the second to fifth cervical vertebrae on the lateral cephalogram. Twenty-seven landmarks from the second to fifth cervical vertebrae were defined below: 1: C2p, the most posterior-lower point of the body of C2; 2: C2a, the most anterior-lower point of the body of C2; 3: C2d, the most deepest point of the lower border of the body of C2; 4: C3up, the most posterior-upper point of the body of C3; 5: C3ua, the most anterior-upper	The repeatability of OE group wasn’t reliable for 5 of 27 landmarks: C2d, C3up, C4up, C4d, C5up ($P<0.05$). The repeatability of ON group wasn’t reliable for 10 of 27 landmarks: C2p, C2a, C3up, C4up, C4la, C4lp, C4d, C5la, C5lp, C5d ($P<0.05$). The repeatability of OE group wasn’t reliable for 7 of 27 landmarks. For ON group, it wasn’t reliable for 11 of 27 landmarks	The repeatability of OE group wasn’t reliable for 5 of 27 landmarks: C2d, C3up, C4up, C4d, C5up ($P<0.05$). The repeatability of ON group wasn’t reliable for 10 of 27 landmarks: C2p, C2a, C3up, C4up, C4la, C4lp, C4d, C5la, C5lp, C5d ($P<0.05$). The repeatability of OE group wasn’t reliable for 7 of 27 landmarks. For ON group, it wasn’t reliable for 11 of 27 landmarks	

			cephalogram. The CACVL group got the initial rough outline by the user, then cervical vertebra landmarks were identified and calculated by fast marching method and parabolic curve fitting. As for the accuracy (the degree of consistency between the measurement value and the true value), three times' mean value of three operators was compared between different groups. As for the repeatability (the result consistency of continuous repeated measurements of the same sample		point of the body of C3; 6: C3la, the most anterior-lower point of the body of C3; 7: C3lp, the most posterior-lower point of the body of C3; 8: C3pm, the middle point of posterior border of C3; 9: C3um, the middle point of upper border of C3; 10: C3am, the middle point of anterior border of C3; 11: C3d, the most deepest point of the lower border of C3; 12-19: C4up-C4d, the same with C3up-C3d in the body of C4; 20-27: C5up-C5d, the same with C3up-C3d in the body of C5.			

			under the same measurement conditions), three times' measurement values coming from a randomly selected operator each group were compared within one group.					

Cote 1997	30	3 examiners, a rheumatologist, an orthopedic spine surgeon, and a radiologist, assessed each radiograph. They all had 10 or more years of experience in their respective specialty.	Lateral cervical spine radiographs were selected from the imagine files at Royal University Hospital in Saskatoon. The selection of the radiographs was performed by the principal investigator from all the films obtained in March 1993. Efforts were made to include a representative number of radiographs with a range of degenerative changes and cervical curvature. The name plate of each radiograph was covered to ensure blind	Lateral cervical radiographs.	A standard procedure was used to measure the sagittal curve. The magnitude of the cervical curve was measured from C1 to C7 and from C2 to C7. The C1-C7 measurement was obtained by drawing a line bisecting the atlas and a line parallel to the inferior end plate of C7. Perpendicular to these two lines were drawn and the angle between them was measured with a standard protractor. The C2-C7 measurement was obtained by joining perpendiculars to	The two-way ANOVA ICC using the examiners as a random sample and its 95% confidence interval (CI) were used to calculate the interexaminer agreement for measurement of the sagittal curves. The precision of the sagittal curve measurements was computed by determining the interexaminer measurement error. the interexaminer measurement error provides an estimate of the 95th percentile of the		

			evaluation. The clinical condition of the patients was unknown to the examiners. Training sessions were arranged with each examiner before the beginning of the study. Each examiner was instructed in the methods of measuring the cervical curvature by the principal investigator. They were selected to minimize any measurement evaluation biases that may be associated with their specialties. To minimize error due to the instruments, the same ruler and protractor were		the lines drawn parallel to the inferior end plates of C2 and C7. If the C7 vertebra was not well visualized on the lateral film, the examiners were instructed to use the inferior end plate of C6.	interexaminer disagreement. To be considered adequate for outcomes research, we considered a variable reliable if its reliability coefficient was greater than or equal to 0.6. The ICC for the C1-C7 measurement is 0.94 (95% CI = 0.90-0.97; F29,58 = 27.5, P < 0.001), which corresponds to excellent agreement. The interexaminer measurement error of the C1-C7 method is 9.1 degrees, indicating that 95% of the measurements		

			used to perform all the measurements by all examiners. The examiners were blind to each other's measurements.			between examiners did not vary by more than 9.1 degrees. The C2-C7 measurement also yields excellent agreement beyond chance, with an ICC of 0.96 (95% CI = 0.88-0.98; $F_{29,58} = 31.8$, $P < 0.001$) and an interexaminer measurement error of 8.3 degrees. The correlation between the C1-C7 and C2-C7 methods is high ($r = 0.84$; $P < 0.0005$). This demonstrates a strong association		

						between the two methods.		

Daffin 2019	The sample consisted of 150 participants aged between 18 and 30 years: 61 males (age - 22.7 ± 3.6; height - 177.5 cm ± 7.1; mass - 79.1 kg ± 14.0) and 89 females (age - 22.5 ± 3.6; height - 166.1 cm ± 6.4; mass - 63.9 kg ± 11.1). All participants were asymptomatic for neck related problems at the time of testing.	All aspects of this study were performed by the same researcher, a board-certified Chiropractor experienced in surface palpation and assessment of postural alignment.	The images of 50 randomly selected participants from the total cohort were assessed two weeks apart to determine the intra-rater reliability of the principal researcher when measuring each of the three postural alignment variables on both photographic and radiological images.	lateral and anterior-posterior cervical radiographs, taken by the same radiographer and digital capturing unit, using established radiological procedures. Participants were instructed to adopt a relaxed neutral erect stance position with their head looking toward the horizon. The participant's trunk was stabilized against the bucky (wall mounted x-ray/digital cassette holder) while the shoulder girdles and arms hung relaxed	Three angles were generated using the landmarks described previously, two sagittal and one coronal. The UCGA is the acute angle formed at the intersection between a horizontal reference line and a line connecting the marker on the left tragus of the ear and the marker on the left lateral canthus of the eye. The UCGA describes the horizontal inclination of the head in the sagittal plane and is related to the relative position of the upper		A two-way mixed effects model (ICC) was used for the measures on the radiological images. ICC of Photogrammetric Measures for UGGA = 0.99, CVA = 1.0, and LHTA = 0.99 showing excellent reliability.	SEM of Photogrammetric Measures for UGGA = 0.4deg, CVA = 0.1deg, and LHTA = 0.1deg.

				by their sides. The body weight was distributed evenly over both feet. The assumed position was not guided by the radiographer, and post-positioning movements were kept to a minimum.	cervical spine. The CVA is the acute angle formed at the intersection between a horizontal reference line and a line connecting the marker on the left tragus of the ear and a point at the skin-marker interface at the spinous process of the seventh cervical vertebra. The CVA describes the inclination of the head with respect to the lower cervical spine. The LHTA is the acute angle formed at the intersection between a horizontal reference line and			

					a line connecting the inferior margins of both ear lobes at the skin-marker interface. The LHTA describes the lateral flexion of the head in the coronal plane.			

Donk 2017	The radiographs from a prospective cohort of 138 patients who participated in a randomized controlled trial were used. A total of 276 lateral radiographs (preoperative and post-operative follow-up) of the cervical spine were retrieved and used. The mean age of the entire group at surgery was 44.3 ± 7 years (range 18.3–59.8 years), and the female	Two investigators who were experienced in measuring angles (H.A. and R.H.M.A.B.) independently calculated the angle between C-2 and C-7.		Upright lateral cervical radiographs.	The Harrison posterior tangent method was used to calculate the angle between C-2 and C-7. The mean angle derived from Harrison's method was then calculated for lordotic, straight, and kyphotic cervical spines (as assessed by the modified Toyama method). Using the modified Toyoma method, lordosis was found on 128 radiographs, kyphosis on 27, and a straight spine on 121.	For both methods the interrater reliability was high: for the posterior tangent method it was 0.907 and for the modified Toyama technique it was 0.984.		

	to male ratio was 1:1.							

Douglas 2007	101 children (32 girls and 69 boys) aged 1 to 12 years.	2 observers (2 sets each).	2 observers (2 sets each).	Lateral digital radiographs obtained with subjects in a supine position and with the head in a neutral position, as recommended by Locke et al.	The ADI was obtained as follows: a digital image showing the cervical spine was displayed; a line was drawn from the inferior margin of the anterior arch of the atlas to the inferior margin of the posterior arch; the distance along this line between the posterior inferior margin of the anterior arch of the atlas and the anterior surface of the dens represented the ADI. In this manner, the points for measurement, particularly at the anterior surface of the dens, could	We assessed intraobserver and interobserver reliability of the ADI measurements with the intraclass correlation coefficient of reliability (R) and its 1-sided 95% confidence interval (CI). R reflects the proportion of the variance of an observation attributable to subject-to-subject variability in the absence of measurement error and increases with increasing reliability. Interobserver	We assessed intraobserver and interobserver reliability of the ADI measurements with the intraclass correlation coefficient of reliability (R) and its 1-sided 95% confidence interval (CI). R reflects the proportion of the variance of an observation attributable to subject-to-subject variability in the absence of measurement error and increases with increasing reliability. Intraobserver	

					be consistently placed.	reliability was assessed twice, namely, on the first and second sets of measurements obtained by the observers. The lowest estimate of interobserver reliability was 0.50 (R Q 0.38).	reliability was 0.72 (R Q 0.64) and 0.85 (R Q 0.80) for 2 observers.	
Dvorak 1988	The healthy population consisting of 28 adults (20 women and 8 men, aged 22–47 years; mean age, 30 years) was available for examination. 31 patients who had suffered soft tissue injury of the cervical	3 independent observers were used in this study. For any particular method of measurement, only 2 independent observers were used.	All functional roentgenograms of the healthy volunteers and patients were measured using both methods. To ensure the independence of the measurements, a set of copies was provided to each observer.	Roentgenogram Examination. Lateral cervical, lateral cervical flexion, lateral cervical extension.	The subjects were standing in an upright position. The left side of the body was closer to the film. The distance between the film and x-ray tube was 150cm. The head was in neutral position, the shoulder hanging down as low as possible in order to show the	For the Penning's method, no statistically significant difference at any level was found when comparing the results of Examiner 1 with those of Examiner 2. Conversely, when comparing the Buetti-Bauml method		

	spine were also examined. The groups consisted of 20 women, aged 23–66 and 11 men aged 21–54; mean age 43.5 years.				cervical-thoracic junction. With two fixing pellets, the mid-thoracic spine and the sternum were fixed in order to avoid flexion-extension of the thoracic spine. The subjects were asked to bend the head forward as much as possible and a film was then taken. In the next step, an active extension was performed and another film was taken in this position. All passive examinations were performed by the first author. The examiner was wearing a whole	of measurement between Examiner 1 and Examiner 3, using the same patient population and the same active and passive roentgenograms, there was a significant inter-observer difference in some of the measured values, at two levels during active and a highly significant difference at one level during passive examination. At an additional four levels there appeared to be a difference between the		

					lead coat with long sleeves, and lead gloves as well as lead glasses. During passive examination, the examiner placed his left hand on the back of the patient's head, the right hand first holding the patient's chin, and inclination at C1-occiput was induced, followed by maximal flexion. Extension was then induced, followed by maximal flexion. Extension was then induced by holding the head with the right and the chin with the left hand. All active and	active and passive measurements that was not statistically validated at the 95% level of confidence.		

					passive functional roentgenograms were measured by using the original Buetti-Bauml method as described previously. The original Penning method was modified by using reverse films for the extension movement. This allowed a more precise superposition of each vertebra during flexion and extension.			

Frobin 2002	From the total number of 137 sets, 101 stemmed from female and 36 from male, skeletally mature subjects in the age range between 16 and 58 yr (mean age 33 yr for both genders). The majority of radiographs stemmed from healthy volunteers who had participated in previous studies conducted by other authors. The remaining radiographs had been taken to rule out a	For determination of segmental motion, the 137 sets of flexion–extension views were processed in approximately equal parts by two observers.	To assess reproducibility in the measurement of rotational and translational motion, seven sets were evaluated by both observers and seven sets were evaluated twice by one observer, but at a 6-month interval. In both cases, evaluation comprised identification and mapping of contours on transparent foils (in no case had marks been placed on the radiographs), digitization and computation of the motion parameters. The two-sided t-test for paired	Lateral radiographic views taken in flexion and extension.	Rotational and Translational motion of C0/C1 (not translational motion), C1/C2, C2/C3, C3/C4, C4/C5, C5/C6, C6/C7 and for all segments, and dens-atlas gap.	Measurement precision of the segmental angle determined from the sets of specimen radiographs ranged for segments C1/C2–C6/C7 between 1.1 and 1.9 with negligible differences between the two observers. For rotational motion determined from in vivo flexion–extension views, inter- and intra-observer tests resulted in SDs of 1.98 and 1.91, respectively. Comparison shows the error in rotational motion (i.e. for	Measurement precision of the segmental angle determined from the sets of specimen radiographs ranged for segments C1/C2–C6/C7 between 1.1 and 1.9 with negligible differences between the two observers. For rotational motion determined from in vivo flexion–extension views, inter- and intra-observer tests resulted in SDs of 1.98 and 1.91, respectively. Comparison shows the error in rotational motion (i.e. for	

	variety of diseases. These sets had all been inspected by radiologists and classified as exhibiting no pathological findings.		measurements, level of significance 0.05, was employed to evaluate the inter- and intra-observer tests.			angle differences) to be slightly inferior to the error of an angle measurement multiplied by sqrt2. The error of displacement determined from specimen radiographs ranged among segments between 0.0181 and 0.0312. Inter- and intra-observer SDs for translational motion determined from in vivo flexion–extension views amounted to 0.0473 and 0.0330, respectively, equivalent to approx. 4.7% or 3.3% of	angle differences) to be slightly inferior to the error of an angle measurement multiplied by sqrt2. The error of displacement determined from specimen radiographs ranged among segments between 0.0181 and 0.0312. Inter- and intra-observer SDs for translational motion determined from in vivo flexion–extension views amounted to 0.0473 and 0.0330, respectively, equivalent to approx. 4.7% or 3.3% of	

						vertebral depth. The magnitude of the dens–atlas gap was determined from specimen radiographs by the two observers with errors of 0.0149 and 0.0135, respectively and from single in vivo extension- or flexion views with inter- or intra-observer SDs of 0.0235 and 0.0257, respectively. The change in the dens–atlas gap was determined from in vivo flexion–extension views with inter-observer or intra-observer SDs of 0.0262	vertebral depth. The magnitude of the dens–atlas gap was determined from specimen radiographs by the two observers with errors of 0.0149 and 0.0135, respectively and from single in vivo extension- or flexion views with inter- or intra-observer SDs of 0.0235 and 0.0257, respectively. The change in the dens–atlas gap was determined from in vivo flexion–extension views with inter-observer or intra-observer SDs of 0.0262	

						and 0.0121, respectively, equivalent to approximately 2.6% and 1.2% of the depth of C2.	and 0.0121, respectively, equivalent to approximately 2.6% and 1.2% of the depth of C2.	

Gadotti 2013a	A total of 22 healthy female subjects participated (mean ± SD: age, 28 ± 4.37 years; body mass index, 22.3 ± 3.24 kg/m2).	1	Codes were placed on top of the subject identification on the radiographs by an independent person using a random digits table, 9 so the second investigator was blind to the subject and sessions (radiographs 1 and 2). Each radiograph was saved with a sequential identification number by an independent person. The digitalized radiographs were analyzed by one investigator. During the measurements, the investigator	Two radiographs of the craniocervical in the sagittal view were taken 1 week apart and at the same time of day.	1. Angles that represent the position of the head in relation to the cervical spine: NSL/odontoid process tangent (OPT) and NSL/cervical vertebra tangent (CVT) 2. Angles that represent the inclination of the cervical spine: OPT/horizontal (OPT/hor.), CVT/horizontal (CVT/hor.), and the cervical inclination angle measured using the centers of the body of the vertebrae C2 and C7 with horizontal (CIAr) 3. Angles that represent the		The order of the radiographs used to do the measurements was randomized using the random digits table to ensure that order was not a factor. Two measurements were performed on each radiograph, and the mean was calculated for each angle. The 2 repeated measures were performed a week apart. The mean for each angle in each radiograph was used to calculate the intrasubject reliability. The mean was then compared between	Small SEM (SEM, 0.25-0.70) were found.

			was blind to the angle values measured because they were calculated automatically by the software in a different window in the computer screen, which was not seen by the investigator.		lordosis of the cervical spine: ARA (absolute rotation angle) and the cervical angle measure using the centers of the body of the vertebrae C2, C4, and C7 (CAr)		radiographs. The intrarater reliability was also calculated between the 2 repeated measurements in the same radiograph. Intraclass correlation coefficient (ICC) and SEM were used to calculate the reliability (intrasubject or reproducibility). The 2-way mixed-average measures were used (ICC 3,k). There is no consensus for cut-point values, but it is usually agreed that the values should be greater than 0.75 to indicate good reliability and	

							less than 0.75 to indicate poor to moderate reliability. Good-to-excellent ICC values and small SEM were found for all the angles measured. The NSL/OPT angle presented a higher standard error of measurement (0.80), but the error is considered small (not clinically significant), and therefore, a good ICC was found (0.97). The intrarater reliability for the same radiographic measurements was also good to excellent (ICC,	

							between 0.98 and 0.99) and SEM between 0.01 and 0.99. Good-to-excellent ICC (ICC, 0.90-0.98).	

Gadotti 2013b	A total of 39 healthy female subjects (age mean, 33 ± 8.03 years old; body mass index, 22.7 ± 2.6) participated.	2	Codes were placed on top of the subject identification on the radiographs by an independent person so the investigator was blinded to each subject. Ten angles were measured on the radiographs.	Lateral craniocervical radiograph.	The following angles were measure on the radiographs:<br>1. The position of the head in relation to the cervical spine: nasion-sella line/odontoid process tangent (NSL/OPT), nasion-sella line/cervical vertebra tangent (NSL/CVT), and Am/spC7/hor. (angle formed by a line connecting the auditory meatus and the spinous process of C7 with a horizontal line);<br>2. The inclination of the cervical spine: odontoid process tangent/horizonta l (OPT/hor),	To test the intrarater and interrater reliability of the measurements on radiographs, the first 22 subjects were included (age mean, 28 ± 4.37 years old; body mass index, 22.3 ± 3.24). The sample size was calculated using a sample size estimation table for reliability analysis. 17 Based on 2 repetitions, 0.05 α and 0.80 power, a minimal sample size of 21 subjects was needed. Intraclass correlation coefficient and	To test the intrarater and interrater reliability of the measurements on radiographs, the first 22 subjects were included (age mean, 28 ± 4.37 years old; body mass index, 22.3 ± 3.24). The sample size was calculated using a sample size estimation table for reliability analysis. 17 Based on 2 repetitions, 0.05 α and 0.80 power, a minimal sample size of 21 subjects was needed. Intraclass correlation coefficient and	Interrater SEM was large and clinically significant for cervical lordosis photogrammetry and for 1 angle measuring cervical lordosis on radiographs.

					cervical vertebra tangent/horizontal (CVT/hor), cervical inclination angle measured using the centers of vertebral bodies of C2 and C7 with the horizontal (CIAr) and using the spinous process of C2 and C7 with horizontal (spCIA);<br>3. The lordosis of the cervical spine: Absolute rotation angle (ARA); cervical angle measure using the centers of vertebral bodies of C2, C4, and C7 (CAr); and using the spinous processes of C2, C4, and C7 (spCA).	SEM were used to calculate reliability.26 A 2-way mixed average measures were used (ICC 3,k). Intraclass correlation coefficient values greater than 0.75 indicate good reliability, and those below 0.75 indicate poor to moderate reliability. Intraclass correlation coefficient greater than 0.90 is considered ideal.20 A 95% confidence interval (CI) was also calculated, and P = 0.05 was used. κ Statistic was	SEM were used to calculate reliability.26 A 2-way mixed average measures were used (ICC 3,k). Intraclass correlation coefficient values greater than 0.75 indicate good reliability, and those below 0.75 indicate poor to moderate reliability. Intraclass correlation coefficient greater than 0.90 is considered ideal.20 A 95% confidence interval (CI) was also calculated, and P = 0.05 was used. κ Statistic was used to	

						used to calculate the interrater reliability of the visual assessment of posture. κ Values above 80% represent excellent agreement; above 60%, substantial agreement; from 40% to 60%, moderate agreement; and below 40%, poor to fair agreement. Interrater reliability was also excellent (ICC, 0.91-0.99). However, CA presented with an SEM of 7.06°, which is considered a clinical significant error	calculate the interrater reliability of the visual assessment of posture. κ Values above 80% represent excellent agreement; above 60%, substantial agreement; from 40% to 60%, moderate agreement; and below 40%, poor to fair agreement. Photogrammetry intrarater reliability was excellent for both raters: ICC of 0.98 to 0.99 and SEM of 0.01° to 0.37°. The intrarater ICC values for radiographic	

						associated with the measurements. Interrater reliability ranged between 0.79 and 0.99. However, spCA presented with a larger SEM (6.89°). Although the ICC values for spCA measurements were considered good (≥0.75), they were the lowest values for both raters (0.96 for rater 1 and 0.94 for rater 2).	measurements were excellent (ICC, 0.95-0.99 for rater 1 and 0.94-0.99 for rater 2) with SEM of 0.01° to 0.99° (rater 1) and 0.06° to 4.03° (rater 2).	

Guyard 2021	119 patients (female:male ratio = 1.5:1; age range: 15–92 years, mean: 43.7 ± 18.5 years; height range: 145–192 cm, mean: 171 ± 8.4 cm)	2	Lateral cervical spine radiographs obtained in the full flexion position were retrospectively and independently analyzed twice by two observers, a junior radiologist (TG) and a junior rheumatologist (ALQ).	Lateral cervical spine radiographs obtained in the full flexion position	For each radiograph, the odontoid sagittal diameter (O), axis body base sagittal diameter (C2), AADI, PADI, Clark station and Ranawat criterion were measured:<br><br>• the odontoid sagittal diameter (O) is the distance from the anterior to the posterior border of the dens;<br>• the axis body base sagittal diameter (C2) is the distance from the anterior to the posterior border of the base of the vertebral body of the axis (excluding osteophytes, if	The ICCs for inter- and intra-observer agreement were excellent (> 0.80) concerning the AADI/O, AADI/C2, PADI/O and PADI/C2 ratios. ICC (95% CI) of: Reader 1 (ALQ); Reader 2 (TG); Reader 1 vs. reader 2 (inter-observer agreement):<br>AADI: 0.96 (0.94–0.97); 0.98 (0.97–0.99); 0.96 (0.95–0.98)<br>PADI: 0.94 (0.92–0.96); 0.96 (0.95–0.97); 0.91 (0.88–0.94)<br>AADI/O: 0.96 (0.95–0.97); 0.98 (0.97–	The ICCs for inter- and intra-observer agreement were excellent (> 0.80) concerning the AADI/O, AADI/C2, PADI/O and PADI/C2 ratios. ICC (95% CI) of: Reader 1 (ALQ); Reader 2 (TG); Reader 1 vs. reader 2 (inter-observer agreement):<br>AADI: 0.96 (0.94–0.97); 0.98 (0.97–0.99); 0.96 (0.95–0.98)<br>PADI: 0.94 (0.92–0.96); 0.96 (0.95–0.97); 0.91 (0.88–0.94)<br>AADI/O: 0.96 (0.95–0.97); 0.98 (0.97–	

					present);<br>• the AADI is the distance from the posterior margin of the anterior C1 arch to the anterior border of the dens;<br>• the PADI is the distance from the posterior margin of the odontoid to the anterior border of the posterior ring of C1;<br>• for the Clark station, the odontoid process is divided into 3 equal parts (“stations”) from cranial to caudal in the sagittal plane. The results are positive if the anterior arch of the atlas is in the second or third station. If the	0.98); 0.80 (0.73–0.86)<br>AADI/C2: 0.96 (0.94–0.97); 0.98 (0.97–0.99); 0.96 (0.94–0.97)<br>PADI/O: 0.87 (0.82–0.91); 0.94 (0.92–0.96); 0.92 (0.89–0.95)<br>PADI/C2: 0.90 (0.86–0.93); 0.94 (0.92–0.96); 0.88 (0.84–0.92)<br>Clarks station: 1; 1; 1<br>Ranawat criterion: 0.85 (0.79–0.89); 0.87 (0.82–0.91); 0.83 (0.77–0.88)	0.98); 0.80 (0.73–0.86)<br>AADI/C2: 0.96 (0.94–0.97); 0.98 (0.97–0.99); 0.96 (0.94–0.97)<br>PADI/O: 0.87 (0.82–0.91); 0.94 (0.92–0.96); 0.92 (0.89–0.95)<br>PADI/C2: 0.90 (0.86–0.93); 0.94 (0.92–0.96); 0.88 (0.84–0.92)<br>Clarks station: 1; 1; 1<br>Ranawat criterion: 0.85 (0.79–0.89); 0.87 (0.82–0.91); 0.83 (0.77–0.88)	

					atlanto-axial facet is level with the middle third (station II) or the caudal third (station III) of the odontoid process, basilar invagination (or vAAS) is diagnosed;<br>• for the Ranawat criterion, the distance between the centre of the second cervical pedicle and the transverse axis of the atlas is measured along the axis of the odontoid process. A distance of less than 13 mm is indicative of vAAS.			

Gwinn 2009	Twenty preoperative, neutral, lateral digital radiographs of the cervical spine in patients undergoing surgical intervention for CSM	3	All radiographs were analyzed by 3 independent observers on 3 separate occasions over the course of 1 week, utilizing 3 separate methods. The 3 observers included a spine fellowship-trained neurosurgeon (M.P.S.), an orthopedic surgeon currently in spine fellowship training (D.E.G.), and a neurosurgical resident (C.A.I).	Neutral, lateral digital radiographs of the cervical spine.	3 separate methods of cervical lordosis (C2-C7 Cobb method, C2-C7 posterior tangent method, and the angle between dorsal-caudal aspect of C2 C7 vertebral bodies).	Intra- and interobserver reliability was excellent (intraclass coefficient > 0.75, kappa > 0.90) utilizing all 3 techniques. Four discrepancies between angular and effective lordotic measurements occurred in which images with a lordotic angular measurement did not have lordosis within the ventral spinal canal. These discrepancies were caused by either spondylolisthesis or dorsally	Intra- and interobserver reliability was excellent (intraclass coefficient > 0.75, kappa > 0.90) utilizing all 3 techniques. Four discrepancies between angular and effective lordotic measurements occurred in which images with a lordotic angular measurement did not have lordosis within the ventral spinal canal. These discrepancies were caused by either spondylolisthesis or dorsally projecting	

						projecting osteophytes in all cases.	osteophytes in all cases.	
Hardacker 1997	100 adult volunteers; Group 1 (no cervical or lumbar symptoms) 25 males, 25 females, mean age 38.4 years (range 23-65 years); Group 2 (lumbar symptoms but no cervical symptoms) 25 males, 25 females, mean age 38.6 years (range 20-67 years)	2	Sagittal plane alignment was measured by a single observer from the foramen magnum to inferior cervical endplates by the Cobb method.	Lateral radiographs	Sagittal plane alignment was measured by a single observer from the foramen magnum to inferior cervical endplates by the Cobb method. Total cervical lordosis (Occiput-C7) and segmental cervical lordosis (O-C1, C1-C2, C2-C3, C3-C4, C4-C5, C5-C6, C6-C7) values were recorded.	Intraobserver angular measurement reliability of total and segmental cervical lordosis values was evaluated by random sampling of 30 radiographs, 15 from each group. Pearson correlations for intraobserver measures revealed r values between 0.67 and 0.95 for each respective	Intraobserver angular measurement reliability of total and segmental cervical lordosis values was evaluated by random sampling of 30 radiographs, 15 from each group. Pearson correlations for intraobserver measures revealed r values between 0.67 and 0.95 for each respective	

						angular value. Similar findings were noted with interobserver reliability measures of total and segmental cervical lordosis values. The primary observer's first and second observations for the total and segmental cervical lordosis shown on the same 30 radiographs were averaged and compared with an independent orthopedist's observation. Interobserver angular measurement r values were between 0.64	angular value. Similar findings were noted with interobserver reliability measures of total and segmental cervical lordosis values. The primary observer's first and second observations for the total and segmental cervical lordosis shown on the same 30 radiographs were averaged and compared with an independent orthopedist's observation. Interobserver angular measurement r values were between 0.64	

						and 0.92, respectively.	and 0.92, respectively.	

Harrison 2000	30 lateral cervical radiographs.	3 Examiners	A delayed repeated-measures design was used to evaluate the reliability of three examiners who digitized the 30 lateral cervical radiographs twice. Thirty lateral cervical radiographs were selected retrospectively. The radiographs were chosen from clinic files with the sole inclusion criterion being that all bony landmarks were clearly visualized without identifying artifacts. Patient identification information was covered on the radiographs, and	Lateral cervical radiographs	The points on the lateral cervical radiograph to be digitized were all anterior–inferior, posterior–superior, and posterior–inferior vertebral body corners of C2–T1, mid-C1 anterior arch, mid-C1 posterior tubercle, posterior lateral mass of C1, angle of the jaw, and symphysis menti. Two distances and fifteen angles were determined. The two translation distances (TzC1–T1, TzC2–C7) were from posterosuperior C1 lateral mass and posterosuperior	Estimated Interclass CC = 7 ICCS > 0.90, 4 ICCS 0.75–0.90, 6 ICCS 0.50–0.75<br>Two horizontal distances and 15 angles were generated from 27 digitized points. The 17 estimated intraclass coefficients were all in the high range (ICCs >0.70) and they were similar to the interclass coefficients, with 6 values in the good range (0.62–0.70) and the other 11 values higher than 0.70.	Estimated Intraclass CC = 8 ICCS > 0.90, 8 ICCS 0.75–0.90, 1 ICCS 0.50–0.75<br>Two horizontal distances and 15 angles were generated from 27 digitized points. The 17 estimated intraclass coefficients were all in the high range (ICCs >0.70) and they were similar to the interclass coefficients, with 6 values in the good range (0.62–0.70) and the other 11 values higher than 0.70.	

			no pencil marks were present on any of the radiographs. Radiographs were submitted in random order to each examiner with a 1-week delay between readings. All examiners were clinicians with2 years, 15 years, and 20 years of experience, and one examiner was also an engineer.		C2 to vertical lines passing through the posteroinferior body corners of T1 and C7, respectively.			

Herrmann 2004	9 patients, 27 lateral cervical radiographs.	4	Three of the examiners were available to repeat these measurements after a minimum of 1 week and a maximum of 7.5 months delay. There were three radiographs from each of nine patients, arbitrarily selected from clinical files, each of whom had undergone anterior cervical discectomy and fusion with anterior cervical plate stabilization at one to four cervical levels. The radiographs were obtained before surgery, within 2 days of surgery, and at 1-	Digitized lateral cervical radiographs	Each radiograph was digitized on a flatbed scanner with a backlit transparency adapter and imported into a graphics software package. Image intensity was adjusted in areas of low contrast on each image to clarify the bony geometry before measurements were made. To do this, the range of intensity was expanded in each area selected to enhance the contrast between light and dark objects. On the clearest image for each patient, the bony contours were traced using a graphics tablet	High intraclass and interclass correlations and low measurement errors were calculated for both distance and angle measurements (Table 2). The distribution of measurement differences (between repeated measures and among examiners) approximated a normal distribution in all instances. The intraexaminer measures for two repeated measurements by three examiners	High intraclass and interclass correlations and low measurement errors were calculated for both distance and angle measurements (Table 2). The distribution of measurement differences (between repeated measures and among examiners) approximated a normal distribution in all instances. The intraexaminer measures for two repeated measurements by three examiners confirmed	Standard errors of measurement were correspondingly low, with mean intraobserver SEMs of 3.26% of interbody distance and 1.20°ofsagittal-plane rotation. Mean interobserver SEMs were3.58% of interbody distance and 1.77°of sagittal-plane rotation. In an average cervical spine,38the mean SEMs of interbody distance would correspond to approximately 0.65 mm (intraobserver) and 0.72 mm.

			to 12-month follow up. The follow-up radiograph selected was obtained before fusion healing had obliterated cortical boundaries. Intervertebral distance and segmental angle were calculated at all surgically involved levels for each patient and at levels immediately adjacent to the surgical construct where visible on the radiograph films. For the reliability analysis, correlated measurements were excluded by randomly		and saved as spline paths within the graphics program. This single set of outlines was used as a template for measuring all other radiographs from the same patient. Before proceeding, the remaining two radiographs had been contrast-enhanced as necessary. Then, the template, consisting of the set of outlines, was copied onto each remaining image and transformed to match the geometry visualized on each image. This transformation	confirmed consistency between repeated measurements for all examiners, with mean ICCs of 0.92 for interbody distance and 0.93 for segmental angle. Interexaminer reliability was also high, with interclass correlations of 0.91 and 0.86 for the first measurement of four examiners for distance and angle, respectively. High intraclass and interclass correlations (ICCs) and low measurement	consistency between repeated measurements for all examiners, with mean ICCs of 0.92 for interbody distance and 0.93 for segmental angle. Interexaminer reliability was also high, with interclass correlations of 0.91 and 0.86 for the first measurement of four examiners for distance and angle, respectively. High intraclass and interclass correlations (ICCs) and low measurement errors were	High intraclass and interclass correlations (ICCs) and low measurement errors were calculated for both distance and angle measurements. Intraexaminer mean ICCs were 0.92 for interbody distance and 0.93 for segmental angle, with standard errors of measurement (SEMs) of 3.26% interbody distance (approximately 0.65mm) and 1.20° sagittal-plane rotation. Mean interexaminer ICCs were 0.91

			discarding data from one measured segment within any three- or four-level construct, leaving no more than two adjacent segments in the data.		consisted of two steps: for the entire template, an overall scale adjustment wherever magnification differences between radiographs occurred; and for each vertebra, a translation and/or rotation of the outline to best fit the radiographic image, along with a scale adjustment when needed to compensate for differences in the axial position of the radiograph source between radiographs. The entire copying and transformation process was	errors were calculated for both distance and angle measurements. Intraexaminer mean ICCs were 0.92 for interbody distance and 0.93 for segmental angle, with standard errors of measurement (SEMs) of 3.26% interbody distance (approximately 0.65mm) and 1.20° sagittal-plane rotation. Mean interexaminer ICCs were 0.91 for interbody distance and 0.86 for segmental angle, with SEMs of	calculated for both distance and angle measurements. Intraexaminer mean ICCs were 0.92 for interbody distance and 0.93 for segmental angle, with standard errors of measurement (SEMs) of 3.26% interbody distance (approximately 0.65mm) and 1.20° sagittal-plane rotation. Mean interexaminer ICCs were 0.91 for interbody distance and 0.86 for segmental angle, with SEMs of 3.58% interbody	for interbody distance and 0.86 for segmental angle, with SEMs of 3.58% interbody distance (approximately 0.72 mm) and 1.77° sagittal-plane rotation.

					accomplished using the graphics program. After all outlines from the index film had been transformed to match the vertebral images on the remaining two films from a given patient, all three sets of outlines were copied onto a single new image. Each element of these outlines was subsequently filled with a uniform black fill against a white back-ground, and the resulting image file was saved. An image-analysis software package was used	3.58% interbody distance (approximately 0.72 mm) and 1.77° sagittal-plane rotation.	distance (approximately 0.72 mm) and 1.77° sagittal-plane rotation.	

					to measure the location of the geometric center of mass (CM) and the area of each outlined element (vertebral body or spinous process). These values were imported into a spreadsheet program to calculate two spinal alignment parameters. Intervertebral distance was computed as the distance between the centers of mass of adjacent vertebral bodies, scaled to account for any differences in magnification between films. The line segment connecting the			

					CM of the vertebral body and the CM of the spinous process of each vertebra was established from the CM coordinates, and its angle relative to the image frame was calculated. Intervertebral angle was computed as the difference between the values of this angle for adjacent vertebrae.			

Jackson 1993	65 subject films were provided from patient records of a primary care private chiropractic clinic.	3	A blind, delayed repeated measures design was used. Three examiners were presented radiographs in random order. All identifying marks were removed prior to each examiner's individual marking and measurement. Each examiner was blinded as to how the previous examiners marked and measured the radiographs.	lateral cervical radiographs	Anterior head translation in millimeters, atlas plane to horizontal, Ruth Jackson's cervical stress lines, and five relative rotation angles for C2-C3, C3-C4, C4-C5, C5-C6, C6-C7. Inter- and intrareliability of the three examiners were statistically analyzed.	Intraexaminer for a) C1 to horizontal reliability was .98-.99 with confidence intervals of .96-.99, b) absolute rotation angle from C2 to C7 reliability was .82-.95 with confidence intervals of .80-.99, c) anterior head translation [+Sz] reliability was .86-.99, with confidence intervals of .74-.99, d) relative rotation angle reliability ranges were (C2-C3) .99, and (C3-C4) .98-.99, (C4-C5) .88-.99, (C5-C6) .80-.99, and (C6-C7) .94-.98. Interexaminer	Intraexaminer for a) C1 to horizontal reliability was .98-.99 with confidence intervals of .96-.99, b) absolute rotation angle from C2 to C7 reliability was .82-.95 with confidence intervals of .80-.99, c) anterior head translation [+Sz] reliability was .86-.99, with confidence intervals of .74-.99, d) relative rotation angle reliability ranges were (C2-C3) .99, and (C3-C4) .98-.99, (C4-C5) .88-.99, (C5-C6) .80-.99, and (C6-C7) .94-.98. Interexaminer	

						reliabilities across examiners ranged from a) Winer:.89-.99 and b) Bartko: .72-.96.	reliabilities across examiners ranged from a) Winer:.89-.99 and b) Bartko: .72-.96.	
Janusz 2016	44 participants, 13 males and 31 females, with a mean age of 15.8 ± 3.7 years.	Three independent orthopaedic surgeons	The measurements were performed on 44 radiographs by one researcher (orthopedic spine surgeon with 5 years of experience) 3 times at 4-week intervals. The order of the radiographs in the second and third series of measurements was different and random. The intraobserver	Standing Lateral Radiographs	On each of the lateral radiographs the angle of CL was measured using the following methods:<br>1. Cobb method C2–C7 (CM)—the angle between the inferior end plate of C2 and the inferior end plate of C7.<br>2. C2–C7 posterior tangent method (PTM)—described as	All methods revealed excellent intra- and inter-observer reliability. Agreement (ICC, SEM) between three methods was (0.89, 3.44), between CM and SPTM was (0.82, 4.42), between CM and PTM was (0.80, 4.80) and between PTM and SPTM was	All methods revealed excellent intra- and inter-observer reliability. Agreement (ICC, SEM) between three methods was (0.89, 3.44), between CM and SPTM was (0.82, 4.42), between CM and PTM was (0.80, 4.80) and between PTM and SPTM was	

			reproducibility was tested and quantified by intraclass correlation coefficient (ICC) and median error for a single measurement (SEM).		the Jackson method or the Gore angle—the angle between the line sustained by posterior margin of C2 vertebral body and the posterior margin of C7 vertebral body. 3. Sum of posterior tangents method (SPTM) for segments C2–C3, C3–C4, C5–C6, C6–C7—described as the Harrison method [4, 7]—sum of the angles measured with the sagittal tangent method at five levels: C2–C3, C3–C4, C4–C5, C5–C6, C6–C7.	(0.99, 1.10). Mean values CL for a CM, PTM, SPTM were 10.5 ± 13.9, 17.5 ± 15.6 and 17.7 ± 15.9 (p\0.0001), respectively. The significant difference was between CM vs PTM (p\0.0001) and CM vs SPTM (p\0.0001), but not between PTM vs SPTM (p [0.05).	(0.99, 1.10). Mean values CL for a CM, PTM, SPTM were 10.5 ± 13.9, 17.5 ± 15.6 and 17.7 ± 15.9 (p\0.0001), respectively. The significant difference was between CM vs PTM (p\0.0001) and CM vs SPTM (p\0.0001), but not between PTM vs SPTM (p [0.05).	

Jiang 2014	Fifty lateral radiographs of patients with single-level cervical degenerative disc	3	Three spine surgeons who each had more than 10 years experience in spine surgery performed the measurements on computers using necessary software. Each spine surgeon was assigned a set of radiographs and allowed to complete the measurements at his own pace over the course of 4 weeks. After three weeks, three spine surgeons rated the same set of images again. The image and the order were blinded and randomized on the two occasions.	lateral plain radiographs of cervical spine in flexion and extension	For each image, three spine surgeons measured the angle on the flexion and extension films according to three methods: Method 1 (Measurement of the angle from the inferior endplate of the vertebra above the degenerative disc to the superior endplate of the vertebra below on the flexion and extension films); Method 2 (Measurement of the angle from the inferior endplate of the vertebra above the degenerative disc to that below the degenerative	Paired comparisons between observers varied considerably with interobserver reliability correlation coefficients ranging from 0.54 to 0.89. Method 2 showed the highest interobserver reliability coefficient (0.82, range 0.73-0.88). Method 2 was also more reliable for the classification of “instability”. interobserver percent agreements ranged from 90% to 98% for	Intraclass correlation coefficients were most consistent for Method 2 (ICC 0.93-0.96) followed by Method 1 (ICC 0.88-0.91) and Method 3 (ICC 0.81-0.87). Intraobserver agreement (% of repeated measures within 0.5° of the original measurement) ranged between 76% and 96% for all techniques, with Method 2 showing the best agreement (92%-96%). Intraobserver percent agreements ranged from 94	

					disc on the flexion and extension films); and Method 3 (Measurement of the angle from the posterior edge of vertebra above the degenerative disc to that below the degenerative disc on the flexion and extension films). Cervical sagittal rotation was defined as the change in the angulation from extension to flexion.	Method 2 versus 86% to 94% for Method 1 and 74% to 84% for Method 3.	to 98% for Method 2 versus 84% to 90% for Method 1 and 78% to 86% for Method 3.	

Johnson 1998	34 young adult women aged between 17.2 and 30.5 years, mean age, 24.5 years	1	To test for intraobserver error, a random selection of 15 unmarked photographs and lateral cephalometric radiographs was measured twice and intraclass correlation coefficients (ICC) were derived for all angles from a repeated measures analysis of variance.	lateral cephalometric radiographs	1. Inclination of the atlas: (At/vert) The angle formed between line AT and true vertical.<br>2. Inclination of the odontoid: (OPT/hor) The angle between the line OPT and true horizontal.<br>3. Cervical inclination: (CVT/OPT) The angle describing the line CVT and true horizontal.<br>4. Cervical lordosis: (CVT/OPT) the angle between the lines CVT and OPT.		The ICCs calculated to examine reliability of repeated measurements of the cervical and craniovertebral angles taken from 10 randomly selected subjects ranged from 0.96-0.99. An ICC of 0.88 was obtained for the 2 complete sets of craniovertebral angles, recorder at least 7 days apart, indicating the consistency of positional tendencies in these subjects. The coefficients for all repeated measurements were in a	

							category considered to be very reliable.	
Kuhr 1996	150 patients (16 male, 134 female) with classic or definite rheumatoid arthritis (mean age 56.5 years, age range 21 to 80 years)	3	To estimate the reliability of the different vertical measurement values, 18 roentgenograms of the upper cervical spine, randomly selected from the ones that could be properly evaluated, were presented to three different observers and then presented again to one of them after 4 weeks. The interobserver	lateral roentgenograms of the cervical spine	Horizontal parameters<br>l. The atlantodental and retrodental distance.<br>2. Distance between the anterior and posterior arches of the atlas.<br>Vertical parameters<br>1. The distance described by McGregor (between the palato-occipital line, or McGregor's line,	The estimation of reliability showed that intraobserver variability was always lower than interobserver variability. The C1/C2 values had higher reproducibility than the values of Ranawat. This shows that the exact definition of the measuring points leads to higher	The estimation of reliability showed that intraobserver variability was always lower than interobserver variability. The C1/C2 values had higher reproducibility than the values of Ranawat. This shows that the exact definition of the measuring points leads to higher reproducibility	

			variability of the three observers and the intraobserver variability of the single observer were estimated.		and the tip of the odontoid).<br>2. The distance described by Redlund-Johnell (between the lower endplate of C2 and the palato-occipital line). In 18.3% of the roentgenograms the Redlund-Johnell method could not be employed because the hard palate was not visible on the roentgenogram.<br>3. The distance described by Ranawat (between the centre of the sclerotic ring and the coronal axis of C1).	reproducibility and greater precision.	and greater precision.	

Kunakornsawat 2017	326 participants with a mean age (SD) of 25.4 years (+/- 3.5) ranged from 20 to 35, and 51.8% males.	3 One orthopedic resident and two fellowship trained spine surgeons evaluated the radiographs.		Standard neutral lateral cervical radiographs	Analysis consisted of measurement of the occiput-C3 angle and posterior occipitocervical angle. Occiput-C3 angle is defined as the angle formed by the line drawn from external occipital protuberance to the tip of superior aspect of posterior vertebral body of C3 intersection to the line drawn from the posterior aspect of the C3 vertebral body. Compare to the posterior occipitocervical angle (POCA) method, which is defined as the	Data were described using frequency and mean (SD) for categorical and continuous data, respectively. Inter- and intra-observer reliabilities were assessed using limit agreement test. In POCA method, the mean difference with 95% limits of agreement of intra- and inter-observer was -0.4 (-13.3, 12.6) and -2.4 (-18.2, 13.4) degrees, respectively, whereas, the mean difference with 95% limits of agreement of intra- and inter-observer of OC3	Data were described using frequency and mean (SD) for categorical and continuous data, respectively. Inter- and intra-observer reliabilities were assessed using limit agreement test. In POCA method, the mean difference with 95% limits of agreement of intra- and inter-observer was -0.4 (-13.3, 12.6) and -2.4 (-18.2, 13.4) degrees, respectively, whereas, the mean difference with 95% limits of agreement of intra- and inter-observer of OC3	

					angle was formed by the intersection of a line drawn tangential to the flat posterior aspect between the occipital protuberance and the foramen magnum and the line determined by the posterior aspect of the third and fourth cervical facets.	method was -0.3 (-5.7, 5.1) and -0.8 (-7.5, 5.9) degrees, respectively.	method was -0.3 (-5.7, 5.1) and -0.8 (-7.5, 5.9) degrees, respectively.	

Lafage 2015	Fifty ASD patients.	Five users (two experienced spine surgeons, three novice spine research fellows).	Five users independently performed each part of the study in two rounds with 1 week between measurements. For the reliability and reproducibility evaluation, users measured 50 unmarked ASD radiographs in two rounds. Intra-class correlation (ICC) and International Organization for Standardization (ISO) reproducibility values were calculated.	Anteroposterior and lateral spinal radiographs (low-dose full-spine, or full-body [head to feet] biplanar stereoradiographic images [EOS imaging, Paris, France] or full-spine 36-inch films).	Cervical parameters included cervical lordosis (CL), T1 slope (T1S), T1 slope-CL mismatch (TS-CL), and C2-C7 SVA (cSVA).	Inter- and intra-observer reliability analyses revealed good (ICC>0.7) or excellent agreement (ICC>0.9) for all parameters. All intra-observer ICC values were >0.9 (excellent), except T1 slope and the related parameter T1S-CL. Intra-observer agreement was highest for the global alignment parameters (ICC>0.970) and the three pelvic CL; ICC>0.840) than parameters based on endpoints and angles (PT, T1SPi, T9SPi,	Inter- and intra-observer reliability analyses revealed good (ICC>0.7) or excellent agreement (ICC>0.9) for all parameters. All intra-observer ICC values were >0.9 (excellent), except T1 slope and the related parameter T1S-CL. Intra-observer agreement was highest for the global alignment parameters (ICC>0.970) and the three pelvic CL; ICC>0.840) than parameters based on endpoints and angles (PT, T1SPi, T9SPi,	

						TPA; ICC>0.970). Inter-observer reproducibility showed angular global alignment parameters had excellent reproducibility, as shown by values of less than 2° (TPA, T1SPI, T9SPI). Pelvic parameter reproducibility values (all <4°) were lower than those for thoracolumbar and cervical parameters (4–6.5°). Reproducibility for both cSVA and SVA was <5 mm.	TPA; ICC>0.970). Inter-observer reproducibility showed angular global alignment parameters had excellent reproducibility, as shown by values of less than 2° (TPA, T1SPI, T9SPI). Pelvic parameter reproducibility values (all <4°) were lower than those for thoracolumbar and cervical parameters (4–6.5°). Reproducibility for both cSVA and SVA was <5 mm.	

Lee 2017	50 subjects, 23 were men (46%) and 27 were women (54%). The mean patient age was 56.6 years (range: 26–77 years).	Three experienced spinal surgeons.	The surgeons were blinded to the clinical information of the patients. The examinations were arranged in a different order before each round of measurement. Three rounds of examinations were performed on three different days with an interval of 1 month to prevent memory bias. Each examination round consisted of each examination analysis with an interval of at least 2 days.	Standing lateral cervical radiography (XR), reconstructed midsagittal images of supine cervical computerized tomography (CT) scans.	C2–C7 distance (C27 SVA), C2–C7 Cobb angle (CL), T1 slope (T1S), thoracic inlet angle (TIA), and neck tilt (NT).	The interobserver ICCs were excellent (ICC range: 0.770–0.996). Interobserver ICC was lowest in XR (0.770–0.983).	The imaging modalities had excellent intraobserver reliability (ICC range: 0.788–0.999). Intraobserver ICC was lowest with XR (0.788–0.999).	

Li 2009	32 cases including 22 females and 10 males (age 20–90 years; mean 55 years).	5 spine surgeons	Each surgeon performed three readings, 7 months apart. In the first round of measurements, the observers used only the Powers' method of instruction. The second and third measurement sets were obtained after an interactive teaching session on the methodology. The order of the images was altered for the second and third set of measurements.	Plain radiographs and spiral CT images of the cervical spine	Powers ratio is the ratio of the length of a line extending from the basion to the posterior arch of the atlas divided by that of a line extending from the opisthion to the anterior arch of the atlas.	When CT scans were used to determine the Powers ratio, the mean Cv value for interobserver reliability was 4.31% for the three readings, representing ‘‘acceptable’’ reliability. Using plain radiographs, a Cv mean value of 9.09% was obtained, signifying that there was ‘‘not acceptable’’ agreement. The limits of agreement for plain radiographs and CT scans were -0.8 and 0.89%, respectively. The two methods	With respect to intraobserver reproducibility of the five observers' assessment of Powers ratio, the Cv values ranged from 1.39 to 9.08% using CT scans, with a mean of 4.95%. This corresponds to a level of ‘‘acceptable’’ reproducibility (Table 1). With the use of plain radiographs, the Cv value for intraobserver reproducibility ranged from 7.54 to 34.30%, with a mean of 14.17%; representing ‘‘not acceptable’’	

						produced results that where within the limits of agreement, suggesting that given these limits, the CT and plain radiographic measurements can be used interchangeably.	reproducibility. Bland-Altman plot analysis showed that given the -0.8 and 0.89% limits of agreement, the two methods may be used interchangeably in clinical practice.	
Lopez 2021	250 patients total, average age 65.6 years (SD 10.3), 143 males, 107 females; 108 LF patients, average age 66.4 years (SD 10.8), 58 males, 50 females; 142 LP patients, average age 64.9 years (SD 10.0), 85	2	Angular measurements were independently performed by 2 orthopedic surgeon reviewers not involved with the care of the patients.	Lateral cervical radiographs	Cervical alignment was measured using the C2−C7 Cobb angle.	Interobserver reliability for cervical alignment measurements was demonstrated with strong correlation (ICC=0.90).		

	males, 57 females.							
Manabe 2022	400 patients of Japanese descent, age range 6-20 years	2	One investigator performed the measurement three times in 3-week intervals, and the average value was used as the measured value; however, the evaluation was verified by another investigator.	Lateral cephalometric radiographs	Anterior nasal spine (ANS) to posterior nasal spine (PNS); Distance between ANS and PNS, Ar-Go; Distance between Articulare and Gonion, Go-Pog; Distance between Gonion and Pogonion. Angular parameters: FMA; Mandibular plane to Frankfort-Horizontal plane,	The κ coefficient for the degree of intra-evaluator agreement was 0.89, and the κ coefficient for the degree of inter-evaluator agreement was 0.91, both showing almost perfect agreement. The measurement error for mandibular height (Ar-Go) was 0.63 mm, as calculated using	The κ coefficient for the degree of intra-evaluator agreement was 0.89, and the κ coefficient for the degree of inter-evaluator agreement was 0.91, both showing almost perfect agreement. The measurement error for mandibular height (Ar-Go) was 0.63 mm, as calculated using	

					SNA; Sella-Nasion to Point A, SNB; Sella-Nasion to Point B. ANS-PNS was measured as an evaluation of the horizontal diameter of the maxilla, Ar-Go was measured as an evaluation of the vertical diameter of the mandible, and Go-Pog was measured as an evaluation of the horizontal diameter of the mandible.	Dahlberg's formula, and that for mandibular length (Go-Pog) was 0.67 mm, which was a sufficiently small error.	Dahlberg's formula, and that for mandibular length (Go-Pog) was 0.67 mm, which was a sufficiently small error.	

Marshall 1996	500 (237 males, 263 females), age range 25-50 y (127 25-29 y; 108 30-34 y; 84 35-39 y; 81 40-44 y; 100 45-50 y)	2	All radiographs were arranged numerically. A large sample increased the power of the study and the statistical relevance of the results. A randomization of subject study was made by both the examiners and the analysis was done independently. One examiner marked the lines and recorded cervical lordotic angle. The lines were left on the films and the second examiner checked and corrected the lines as deemed necessary and then re-measured	The neutral lateral cervical radiographs were obtained using standardized positioning according to the following protocols: 1.The patient is positioned in the upright lateral standing position; 2.In patients with scoliosis, the patient was positioned such that the convex side of the scoliosis was placed adjacent to the film; 3.The shoulder is in contact with the cassette holder; 4.The head and neck are positioned in the true neutral	The C1-7 angle of the cervical curve. This procedure involves constructing a line intersecting the anterior and posterior tubercles of C1 (atlas plane line). A second line is then constructed through the inferior aspect of the C7 body. Two more lines are then drawn perpendicular to the first two lines. The angle of the intersecting lines formed is expected to fall within an arbitrary range of 30°-45° to be considered within normal limits. Less than 30° is	The normal range for lordosis is 30°-45° inclusive. For the 500 patients, measurement of lordosis showed anormal (i.e. classically bell-shaped) distribution. The mean magnitude of absolute differences in rating between examiner 1 and examiner 2 was 0.9°. No difference for any patient was greater than 3°, which is less than 10% of the mean rating of either examiner. The differences of larger magnitude (2° or 3°) occurred in		

			the angles and recorded them.	position with the shoulders depressed as much as possible;<br>5.Full expiration at the time of exposure;<br>6.The central ray positioned at C4(6);<br>7.The source-imaging distance (SID) is 1.83 meters (72 inches).	considered hypolordosis, while more than 45° is considered hyperlordosis.	patients with mid-range lordosis. The differences were symmetrically distributed about zero which indicates that neither rate is systematically rating higher or lower than the other. This conclusion is supported by the mean signed difference being effectively zero at 0.04 with a 95% CI (-0.07, 0.14). The mean signed difference allows for + and - signs and is calculated by subtracting examiner 2 ratings from examiner 1. The		

						confidence interval indicates that there is a 95% probability that the true mean difference between the two raters (the mean amount by which rater number 1’s ratings are greater than rater number 2’s) is between -0.07 and 0.14. Observing the standard deviation of differences between examiners for individual patients, 95% of the difference will lie within two standard deviations of the mean difference.		

						As mentioned above, the mean signed difference is 0.04, i.e. effectively zero. The standard deviation of differences is 1.17 so that 95% of differences in ratings between individual patients in the population from which this sample is drawn are predicted to lie in the range -2.31 to 2.38. This is a small range considering the range of lordosis measurements recorded in the study.		

Nakamura 2014	50 patients (24 boys and 26 girls), median age of the subjects at the time of the initial cervical spine assessment was 3.1 years (range: 0.4–15.0 yr).	3	Naoyuki Nakamura (a pediatric spinal surgeon) and Yogen Morikawa and Masatoshi Oba (with 5 and 2 yr of experience in orthopedic surgery) measured the novel indices in 15 randomly selected lateral radiographs of the cervical spine in the neutral position, and the inter/intraobserver reliability was assessed using the intraclass correlation coefficient for the measurements. Moreover, the lead author and the residents measured the indices again after	Neutral lateral cervical radiographs.	Maximum ADI, the minimum space available for the spinal cord (C1 SAC), and the instability index using the lateral dynamic radiographs of the cervical spine (in the maximal flexed, neutral, and maximal extended positions) taken during the final visit (in the surgical group, during the preoperative examination). The C1 inclination angle and the C1/4 SAC ratio of the cervical spine in the neutral position were measured as 2 novel indices.	For the interobserver and intraobserver reliability of the novel indices, the correlation coefficients were in the range from 0.88 to 0.99. Correlation was observed between the ADI and C1/4 SAC ratio ($r = 0.507$, $P < 0.01$) and between the ADI and C1 inclination angle ($r = 0.407$, $P < 0.01$).	For the interobserver and intraobserver reliability of the novel indices, the correlation coefficients were in the range from 0.88 to 0.99. Correlation was observed between the ADI and C1/4 SAC ratio ($r = 0.507$, $P < 0.01$) and between the ADI and C1 inclination angle ($r = 0.407$, $P < 0.01$).	

			an interval of 1 month or longer, and the intraobserver reliability was assessed using the Intraclass correlation coefficient.		The C1 inclination angle was defined as the angle formed between the fore perpendicular to the tangent at the posterior surface of the C2 body and the line connecting the centers of the C1 anterior and posterior arches. The cranial inclination in which the C1 anterior arch was elevated was considered positive, and the caudal inclination in which the anterior arch was lowered was considered negative. The C1/4 SAC ratio was defined as the ratio of the			

					anteroposterior diameter of the spinal canal at the C1 level to the anteroposterior diameter of the spinal canal at the C4 level.			
Paholpak 2018	Forty-six patients (25 male and 21 female, ages 45.41 ± 9.32 years old)	3		Cervical dynamic plain radiograph	The C2–7 angle (cervical lordotic measurement) was measured as the angle between the tangent lines of the lower endplates of the axis and C7 (Fig. 1). The positive value was the kyphotic alignment and the negative value was the lordotic alignment. The	The ICCs of intra- and inter-observer between three spine observers showed excellent reliability in all measured parameters; the ICCs were 0.762–0.968.	The ICCs of intra- and inter-observer between three spine observers showed excellent reliability in all measured parameters; the ICCs were 0.762–0.968.	

					C2–3 angle (C2–3 segmental angle) was defined as the angle between the tangent lines of the lower endplate of the axis and the tangent lines of the lower endplates of C3 (Fig. 2). The subaxial segmental angles (C3–4, C4–5, C5–6, and C6–7) (segmental angle) were measured as the angle between the tangent lines of the upper endplates of the cephalad cervical spine and the tangent lines of the lower endplates of the caudad cervical spine.			

Phillips 1999	30 subjects, 20-60 years (18 men, 12 women, mean age, 42 years)	2	2 investigators calculated and documented both of these measurements on all radiographs in a blinded manner. The 2 parameters were measured by both observers on 3 occasions, and the average was used for comparisons.	Standard lateral cervical radiographs in neutral, flexion, and extension	Occipitocervical angle (OCA) defined by the junction of McRae's line (a line intersecting the basion and the opisthion) and a line drawn parallel to the superior endplate of C3, and occipitocervical distance (OCD) obtained by measuring the shortest distance from the superior most aspect of the C2 spinous process to the occipital protuberance.	The interobserver correlation coefficients for the OCA were 0.83, 0.91, and 0.89 in neutral, flexion, and extension, respectively. The coefficients for the OCD were 0.91, 0.88, and 0.87 in neutral, flexion, and extension respectively.		

Plaugher 1990	49 patients, 31 females, 18 males, 44 years (+/- 20 years)	3	Interobserver reliability for the retrolisthesis measurement was determined with 3 examiners and 20 different cases. 1 examiner marked 20 cases again to determine intraexaminer reliability. Cervical sagittal curve was measured from C2-C7 and from C1-C7. The vertebral body end-plate lines were constructed by drawing a line perpendicular to George's line. The angle was rounded to the nearest 0.5 degrees. Both films were placed on the radiographic	Lateral full spine radiograph	Cervical retrolisthesis. This procedure consisted of making a dot at the AS and one at the PS corner of subjacent vertebral bodies of a cervical retrolisthesis. The superior segment then had a dot placed at the PI corner of the it's vertebral body. The subjacent dots were then connected with a line and another line was descended at the PS point perpendicular to the horizontal line. A parallel line was then descended at the PS point perpendicular to	The retrolisthesis measurement was determined to have excellent interexaminer reliability (0.74-0.83). The cervical lordosis measurement was determined to have excellent interexaminer reliability (0.89-0.94).	The retrolisthesis measurement was determined to have excellent intraexaminer reliability (0.90). The cervical lordosis measurement was determined to have excellent interexaminer reliability (0.97).	

			viewer at the same time to make it easier to determine similar anatomic points. Inter- and intraexaminer reliability with standard errors of estimate and 95% confidence intervals was calculated for the measured cervical lordosis (C1-C7).		the horizontal line. A parallel line was then descended from the superior segment and the distance separating the 2 lines is measured. Cervical sagittal curve was measured from C2-C7 and from C1-C7. The vertebral body end-plate lines were constructed by drawing a line perpendicular to George's line.			

Rochester 1994	10 sets of upper cervical films.	4	Films were randomly selected from the patient files of a doctor not participating in the study. The films were checked for identifying artifacts and labeled A–J covering the names of the patients. The doctors then read the films with approximately 1 week between readings. Doctors 1 and 3 read the films twice manually and twice with the computer. Doctors 2 and 4 read the films once manually and once with the computer. All marks were	Lateral cervical, Nasium, and Vertex view.	The study was to research the measurement of atlas laterality and rotation, odontoid, C2 spinous and lower angle literalities. The height factor calculation with components atlas plane line, condylar surface and axial surface circles were also examined.	The reliability for each of the four experts is at an acceptable level for all aspects of the upper cervical marking procedure. The reliability estimates ranged from 0.83-0.94 for atlas laterality, 0.84-0.96 for odontoid, 0.89-0.96 for C2 spinous and 0.86-0.96 for lower angle. Rotation estimates are lower at 0.54–0.68.<br>The mean rotation estimate for only manual readings is 0.87 and for the computer	The reliability for each of the four experts is at an acceptable level for all aspects of the upper cervical marking procedure. The reliability estimates ranged from 0.83-0.94 for atlas laterality, 0.84-0.96 for odontoid, 0.89-0.96 for C2 spinous and 0.86-0.96 for lower angle. Rotation estimates are lower at 0.54–0.68.<br>The mean rotation estimate for only manual readings is 0.87 and for the computer	

			thoroughly cleaned, the films reordered and relabeled A–J between each reading.			readings is 0.82. When you compare the mean measurement for rotation of the manual readings at 1.47 degrees to 1.86 degrees for the computer mean, it is demonstrated that the two systems are slightly different but within an acceptable tolerance. The height factor reliability estimates range from 0.81 to 0.97. The plane line estimates range from 0.96 to 0.98. The reliability for the condylar and axial surface measurements	readings is 0.82. When you compare the mean measurement for rotation of the manual readings at 1.47 degrees to 1.86 degrees for the computer mean, it is demonstrated that the two systems are slightly different but within an acceptable tolerance. The height factor reliability estimates range from 0.81 to 0.97. The plane line estimates range from 0.96 to 0.98. The reliability for the condylar and axial surface measurements	

						are lower overall ranging from 0.29 to 0.88. The manual intraexaminer readings for condylar and axial surface measurements range from 0.29 to 0.88 compared to 0.81 to 0.98 for the computer readings. Examining the standard deviation of the condylar surface at 0.18 to 0.46 inches compared to 0.56 to 1.74 inches for the axial surface reveals that the axial surface measurement is the weakest component of the height factor	are lower overall ranging from 0.29 to 0.88. The manual intraexaminer readings for condylar and axial surface measurements range from 0.29 to 0.88 compared to 0.81 to 0.98 for the computer readings. Examining the standard deviation of the condylar surface at 0.18 to 0.46 inches compared to 0.56 to 1.74 inches for the axial surface reveals that the axial surface measurement is the weakest component of the height factor	

						calculation causing about 1 inch of fluctuation in the height factor. Even with this weakness, the height factor reliability is still very good overall. Reliability for the four doctors is very good when evaluating the standard deviation. The standard deviation for atlas laterality ranged from 0.36-0.55 degrees with a mean of 0.45 degrees (approximately 0.30mm). The lower angle ranged from 0.50-0.74	calculation causing about 1 inch of fluctuation in the height factor. Even with this weakness, the height factor reliability is still very good overall. Reliability for the four doctors is very good when evaluating the standard deviation. The standard deviation for atlas laterality ranged from 0.36-0.55 degrees with a mean of 0.45 degrees (approximately 0.30mm). The lower angle ranged from 0.50-0.74	

						degrees with a mean of 0.59 degrees. The atlas rotation ranged from 0.44-0.70 degrees with a mean of 0.59 degrees. The height factor standard deviation ranged from 0.35 inches to 0.93 inches with a mean of 0.50 inches. The estimates for the inter-examiner reliability via the One Way ANOVA is 0.83 for atlas laterality, 0.88 for lower angle, 0.46 for rotation and 0.83 for the height factor.	degrees with a mean of 0.59 degrees. The atlas rotation ranged from 0.44-0.70 degrees with a mean of 0.59 degrees. The height factor standard deviation ranged from 0.35 inches to 0.93 inches with a mean of 0.50 inches. The estimates for the inter-examiner reliability via the One Way ANOVA is 0.83 for atlas laterality, 0.88 for lower angle, 0.46 for rotation and 0.83 for the height factor.	

Salameh 2021	Demographics were as follow: ASD: 41 ± 13 years, 65 ± 13 kg, 166 ± 8 cm, 8 M/21 F; AIS: 14 ± 2 years, 54 ± 8 kg, 163 ± 7 cm, 3 M/17 F; control group: 31 ± 13 years, 70 ± 18 kg, 165 ± 9 cm, 9 M/12 F.	4	Several cervical parameters were measured by 4 operators, three times each.	Full body lateral radiographs	C0-C2 angle: between lower endplate of C2 and McRae line (line drawn from anterior to posterior edges of foramen magnum). C1-C2 angle: between lower endplate of C2 and axis of C1 (line drawn from most anterior to posterior aspects of C1). C2 slope (C2s) [13]: between lower endplate of C2 and a horizontal line. Cervical sagittal vertical axis (cSVA): distance between C2 plumb line and postero-superior edge of C7. Cranial tilt (Crant): between line from center	ICC and the 95% CI were similar between the 3 groups. The measured parameters showed a very high repeatability (ICC > 0.8) except for C0-C2, which presented an average repeatability (ICC = 0.57). Cranial tilt had a 95% CI < 2 (◦ or cm).	ICC and the 95% CI were similar between the 3 groups. The measured parameters showed a very high repeatability (ICC > 0.8) except for C0-C2, which presented an average repeatability (ICC = 0.57). Cranial tilt had a 95% CI < 2 (◦ or cm).	

					of superior end-plate of T1 and dens and a vertical line. Cervical tilt (Cervt): between perpendicular to center of superior endplate of T1 and a line from this center to tip of dens. Cranial offset (Cranoff): distance between a vertical line passing by center of the body of C7, and a vertical line passing through external auditory meatuses.			

Shin 2016	80 consecutive AS patients (of 40 non-ankylosis and 40 ankylosis patients), 10 women, 70 men of average age 43.4 ± 12.3 years	3	A delayed and repeated measurement design was used to evaluate the reliability of the three examiners. All 80 radiographs were measured twice by three examiners with 2 weeks delay between first and second measurements. A total of 400 measurements were taken for each of the five different radiographic methods.	Plain cervical lateral radiography	The five methods included the Cobb C1-C7, Cobb C2-C7, centroid, posterior tangent C2-C7, and Ishihara index methods.	The intra- and interclass correlation coefficients (ICCs) of all five methods were generally high. For the 80 study subjects, ICCs were ≥0.84 (excellent) for all five radiographic methods. However, comparison of the ICCs, 95% confidence intervals (CI) and mean absolute differences (MAD) between groups with varying degrees of ankylosis showed that the reliability of lordosis measurements	The intra- and interclass correlation coefficients (ICCs) of all five methods were generally high. For the 80 study subjects, ICCs were ≥0.84 (excellent) for all five radiographic methods. However, comparison of the ICCs, 95% confidence intervals (CI) and mean absolute differences (MAD) between groups with varying degrees of ankylosis showed that the reliability of lordosis measurements	

						decreased as the severity of ankylosis increased. The five methods consistently demonstrated higher ICCs for both inter- and intra-observer comparisons in the non-ankylosis group. However, in the ankylosis group, the Cobb C1-C7 method demonstrated high ICCs for both inter- and intra-observer comparisons whereas the other 4 methods had high ICCs only for intra-observer comparisons. The intraobserver	decreased as the severity of ankylosis increased. The five methods consistently demonstrated higher ICCs for both inter- and intra-observer comparisons in the non-ankylosis group. However, in the ankylosis group, the Cobb C1-C7 method demonstrated high ICCs for both inter- and intra-observer comparisons whereas the other 4 methods had high ICCs only for intra-observer comparisons. The intraobserver	

						MADs were similar for the five methods (2.4-3.9), but the interobserver MADs of measurement methods in both groups showed low measurement reliability except for the Cobb C1-C7 methods.	MADs were similar for the five methods (2.4-3.9), but the interobserver MADs of measurement methods in both groups showed low measurement reliability except for the Cobb C1-C7 methods.	

Shoda 2004	30 healthy volunteers	Five spinal surgeons with more than 10 years of practice evaluated the radiographs.	The 30 radiographs were measured by each surgeon in separate rooms, all using the same type of ruler and a soft-lead marking pencil. Additionally, on the first assessment, doctors recorded the most difficult landmark to identify for each of the 30 radiographs. Three of the doctors measured each film twice with at least a 2-week interval. All film tags were concealed, and all drawings on the films were erased after each measurement by the controller	Lateral cervical radiographs	The Chamberlain line, McRae line and McGregor were drawn, and the angle between the first and each occipital line was measured.	Mean interobserver variances of the angles using Chamberlain line, McRae line, and McGregor line were 2.3° (from 0.4°–6.4°), 5.0° (from 1.8°–11.9°), and 1.4° (from 0°–4.5°), respectively; interobserver intraclass correlation coefficients were 0.939, 0.802, and 0.972. The highest reliability indexes were obtained for McGregor line.	Intraobserver intraclass correlation coefficients of the angles were 0.956, 0.835, and 0.975. The highest reliability indexes were obtained for McGregor line.	

			(N.S.). To determine intraobserver variance, we examined the differences between the 2 measurements of the same curve by the same observer. The interobserver variance was estimated by first calculating the standard deviation for each of the 30 groups of 5 measurements and then averaging these values.					

Sigler 1985	20	3 experienced upper cervical practitioners.	Intra-examiner reliability was tested by recording the selected films original measurements. Since the films were taken directly from patient files, they still have the original pencil measurements that were used to determine how to adjust that particular patient. These markings were recorded, then erased, and the film ID tag was covered as to block out any patient information. None of the films selected at random had any identifying	Not identified.	The measurement used in this study was the atlas laterality measurement. This measurement is made by drawing a line through the inferior attachment points to the atlas posterior arch with the lateral margins of the lateral masses. Another line representing the major axis of the skull is drawn with the aid of a transparent skull divider template. The angle formed by these two intersecting lines is used to determine the lateral displacement of	ICCs:<br>Observers: 1v2 = 0.70; 1v3 = 0.78; 2v3 = 0.84<br>Pearson's r:<br>Observers: 1v2 = 0.75; 1v3 = 0.91; 2v3 = 0.89	ICCs:<br>Observers: 1v1 = 0.77; 2v2 = 0.86<br>Pearson's r:<br>Observers: 1v1 = 0.82; 2v2 = 0.89	Doctor 1:<br>Absolute Mean = 1.55; MAD = 1.10<br>Doctor 2:<br>Absolute Mean = 2.01; MAD = 0.82<br>Doctor 1-3:<br>Absolute Mean = 1.83; MAD = 1.05

			artifacts.<br><br>The films were numbered from one to 20, then divided and given to the three participating doctors. When the doctors completed their markings, the experimenter collected the films, recorded the markings, erased the markings, then exchanged films until each doctor had marked all the films. Since two doctors had originally marked 10 of the films, comparison to findings the second time they marked them (during the		the atlas with respect to the condyles of the occiput.			

			experiment) was used for the intra-examiner comparison.<br><br>Examiner reliability was able to be tested since three doctors measured 20 films each for a total of 60 comparisons.					

Silber 2004	Twenty lateral cervical radiographs of nonspondylotic spines (group 1) and 20 lateral cervical radiographs of spondylotic spines (group 2)	3 Orthopedic surgeons	Three examiners who were of varying levels of experience performed measurement of sagittal alignment on each radiograph. The participants included an attending spine surgeon (examiner 1), a chief orthopaedic resident (examiner 2), and a junior orthopaedic resident (examiner 3). The examiners measured each radiograph three times, with at least 1 week separating each session. Bias was eliminated by blinding each	Lateral cervical radiographs	Gore and Cobb techniques for measuring sagittal alignment of the cervical spine was performed.	For group 1, there was less intra- and interobserver variability for the Gore method than for the Cobb method ($P < 0.05$). Group 2 measurements were also less variable for the Gore method, although this was not statistically significant. Pooling all three observers, 95% confidence limits for intra- and inter-observer variability for the Gore method were 3° and 6° for group 1 and 4° and 7° for group 2, respectively. For	For group 1, there was less intra- and interobserver variability for the Gore method than for the Cobb method ($P < 0.05$). Group 2 measurements were also less variable for the Gore method, although this was not statistically significant. Pooling all three observers, 95% confidence limits for intra- and inter-observer variability for the Gore method were 3° and 6° for group 1 and 4° and 7° for group 2, respectively. For	

			observer to his previous measurements and to that of the other observers. The end vertebrae for each measurement were predetermined. The C3 and C7 vertebral bodies were chosen. All measurements were made using the same soft-lead, fine-tipped pencil and protractor. On each radiograph, the examiners performed both Gore and Cobb angle measurements. For the Gore angle measurements, the examiners used the posterior			the Cobb method, corresponding values were 4° and 9° for group 1 and 5° and 9° for group 2. Overall, intraobserver measurements were less variable than interobserver measurements ($P < 0.01$). There were no significant differences in variability based on experience level.	the Cobb method, corresponding values were 4° and 9° for group 1 and 5° and 9° for group 2. Overall, intraobserver measurements were less variable than interobserver measurements ($P < 0.01$). There were no significant differences in variability based on experience level.	

			vertebral body lines of C3 and C7 as reference points, while for the Cobb angle measurements, the examiners used the superior endplate of C3 and the inferior endplate of C7 as reference points. After each radiograph was measured and the results were recorded, the examiner used an alcohol prep pad to erase any markings made on the radiographs. All measurements were rounded to the nearest degree.					

Svedmark 2011	Nine patients (five men, four women) with a median age of 42 years (range 38 – 56 years)	1	To establish repeatability, the same observer in a second trial 24 hours later repeated the above landmark selection procedure for each patient case and each model pseudo case.	2 CT scans of each patient were obtained, one scan with the cervical spine in flexion and one in extension.	The following is our procedure for measuring the relative kinematics of two cervical spine prosthetic components (an 'upper component' and a 'lower component') between two CT scans of the same region. Using the image analysis tool, the user (a) visualizes the upper and lower components as 3D isosurfaces in both CT volumes simultaneously, and then (b) manually places landmarks on these isosurfaces at corresponding points in both CT volumes. This		Analysis of both the model and patients showed good repeatability, i.e. within 2 standard deviations of the mean using the 95% limits of agreement with no overlapping confidence intervals. The accuracy analysis showed that the median error was close to zero.	

					completes the manual process. The image analysis tool automatically performed all remaining steps. It (c) spatially aligns the two CT volumes in 3D such that the upper components coincide, (d) defines a new coordinate system based on the upper components and aligned with the coordinate system of the CT scanner, (e) calculates the orientation of the lower components in each volume in this new coordinate			

					system, and (f) calculates any change in orientation of the lower components (movement) between the two volumes.			
Tang 2020	150 asymptomatic subjects (73 males and 77 females), mean age 48.0 ± 8.4 years old (range 20–69 years old; 48.4 ± 10.2 years old for males and 47.6 ± 6.4 years old for females).	2 spine surgeons	Two spine surgeons calculated and documented the OC4D and OCD in each of the tested cervical radiographical positions in a blinded manner. These parameters were measured by both observers on three occasions over an interval of at least 2 weeks.	Neutral, flexion, and extension lateral cervical spine radiographs	Analysis of lateral neutral, flexion, and extension radiographs was performed using our novel OC4D measurement method. The OC4D was defined as the shortest distance from the center of the C4 vertebral body to the McGregor's line. In contrast, the occipitocervical distance (OCD)	The inter-observer reliabilities of OC4Ds were found to have ICCs of 0.945, 0.953, and 0.961 in neutral, flexion, and extension positions, respectively. Additionally, the ICC values in terms of intra-observer reliabilities for OC4Ds were 0.981, 0.972,	The inter-observer reliabilities of OC4Ds were found to have ICCs of 0.945, 0.953, and 0.961 in neutral, flexion, and extension positions, respectively. Additionally, the ICC values in terms of intra-observer reliabilities for OC4Ds were 0.981, 0.972,	

					was defined as the shortest distance between the most superior aspect of the C2 spinous process and the occipital protuberance.	and 0.968 in neutral, flexion, and extension positions, respectively. Hence, all ICC values for both inter-and intra-observer reliabilities of OC4Ds in neutral, flexion, and extension positions were excellent, as they were all above 0.93. No significant difference was found between the measurements made by a single observer and those made by different observers. Finally, ICCs of inter- and intra-observer	and 0.968 in neutral, flexion, and extension positions, respectively. Hence, all ICC values for both inter-and intra-observer reliabilities of OC4Ds in neutral, flexion, and extension positions were excellent, as they were all above 0.93. No significant difference was found between the measurements made by a single observer and those made by different observers. Finally, ICCs of inter- and intra-observer	

						reliabilities for OC4D were significantly higher than those for OCD in each tested position ($P < 0.05$).	reliabilities for OC4D were significantly higher than those for OCD in each tested position ($P < 0.05$).	

Vidal 2013	75 patients divided in three groups (control subjects, AIS, operated AIS). Mean age in the control group was 12.24 ± 3.21 years. Sex ratio was 17 girls for 8 boys. Age in the non-operated AIS group averaged 11.7 ± 1.1 years. Sex ratio was 19 girls for 6 boys. Mean age in the operated group was 12 ± 2.5 years. Sex ratio was 18 girls for 7 boys.	3	Full-spine radiographs were analyzed twice in a randomized order at 1-week interval by three independent observers: a senior pediatric spine surgeon, a senior pediatric radiologist and an orthopedic resident (total: 4,950 measures). Five full-spine lateral radiographs were used by the three observers as a common training and then excluded from the study. Each observer collected eleven angles and distances for each patient.<br>Three observers digitally analyzed	Lateral full-spine low-dose EOS radiographs	The following spinal parameters were measured:<br>•C1C3 angle: measured between a line joining the upper extremity of the C1 anterior arch and the upper extremity of the C1 posterior arch and the lower endplate of C3<br>•C3C7 angle: measured between the upper endplate of C3 and the lower endplate of C7<br>•C2C6 angle: measured between the lower endplate of C2 and the lower endplate of C6	Reliability was assessed calculating intraobserver Pearson's r correlation coefficient, interobserver intra-class correlation coefficient (ICC) completed with a two-by-two Bland–Altman plot analysis. There was no significant difference of ICC between the three groups of patients, meaning that inter and intraobserver reliability were not affected by the deformity and/or spinal instrumentation.	Reliability was assessed calculating intraobserver Pearson's r correlation coefficient, interobserver intra-class correlation coefficient (ICC) completed with a two-by-two Bland–Altman plot analysis. There was no significant difference of ICC between the three groups of patients, meaning that inter and intraobserver reliability were not affected by the deformity and/or spinal instrumentation. Overall values	

			twice each radiograph and 11 sagittal measures were collected for each image.			Overall values (Table 3): for examiner 1, r values ranged from 0.889 (±2 standard deviation (SD): 0.8616–0.9768) to 0.999 (0.9992–0.9999), best r value was obtained for CAEPL and worst was obtained for C2C6.<br>For examiner 2, r ranged from 0.840 (0.8027–0.9661) to 0.953 (0.9407–0.9904), best value was CAEC7 and worst was T1T12. For examiner 3, r values ranged from 0.871	(Table 3): for examiner 1, r values ranged from 0.889 (±2 standard deviation (SD): 0.8616–0.9768) to 0.999 (0.9992–0.9999), best r value was obtained for CAEPL and worst was obtained for C2C6.<br>For examiner 2, r ranged from 0.840 (0.8027–0.9661) to 0.953 (0.9407–0.9904), best value was CAEC7 and worst was T1T12. For examiner 3, r values ranged from 0.871 (0.8534–0.9222)	

						(0.8534–0.9222) to 0.965 (0.9192–0.9866). Highest value was obtained for CAEH distance and worst for C2C6 angle. Interobserver ICC ranged from 0.803 (T1T12 angle) to 0.991 (C7PL distance), showing excellent interobserver reliability. Bland–Altman plot analysis depicted the difference between two observers against the average angle (or distance) from two observers on the same	to 0.965 (0.9192–0.9866). Highest value was obtained for CAEH distance and worst for C2C6 angle. Interobserver ICC ranged from 0.803 (T1T12 angle) to 0.991 (C7PL distance), showing excellent interobserver reliability. Bland–Altman plot analysis depicted the difference between two observers against the average angle (or distance) from two observers on the same radiograph. Dots included	

						radiograph. Dots included between the +2 SD and -2SD lines are considered as excellent interobserver agreement marks. This analysis showed no systemic error between examiners when reviewed by pairs.	between the +2 SD and -2SD lines are considered as excellent interobserver agreement marks. This analysis showed no systemic error between examiners when reviewed by pairs.	

Wellborn 2000	72 patients with Down syndrome; average age 4.7 years (range, 2-15 years)	3	Three blinded observers independently measured each radiograph. The senior author (P.F.S.) instructed the two junior authors in measurement techniques. Each rater interpreted the radiographs independently and did not know how they had been interpreted by the other raters. The raters were allowed unrestricted time on each radiograph. All marks were erased after each measurement. The radiographs were measured by each reader on three separate	Lateral flexion-extension radiographs	ADI, Power's ratio, Wiesel Rothman, and the occipitoatlantal angle.	The interobserver correlation coefficient also was computed across the three observers for each of the five assessments. This coefficient quantifies whether the overall measurements are reliable on a repeated measure regardless of who is doing the scoring. The best agreement is on the ADI (again with only fair agreement), and the worst is among the Power's ratio measurements.	Among two of three raters (two and three), the ADI had the highest reproducibility with coefficients of 0.51 and 0.55, respectively. The coefficient for observer one was only 0.19, raising the possibility of experience as a major variable. For all other measurements, the reproducibility was poor. For Wiesel-Rothman, the correlation coefficient ranged from a low of 0.17 for observer two to a high of 0.45 for observer one.	

			occasions with at least a 1-month interval between measurements. At subsequent evaluations, the radiographs were presented in a different numeric order to guard against any recall bias from the first interpretation. The readers consisted of an attending pediatric orthopaedic surgeon, a pediatric orthopaedic fellow, and a PGY-4 orthopaedic resident.				The correlation for Kuhn's angle ranged from 0.15 for observer three to 0.4 for observer one. For Power's ratio, the range was from 0.05 for observer two to 0.33 for observer one. The ADI and Wiesel-Rothman measurement had the highest agreement among raters at 0.5 and 0.42, respectively. This indicates a fair level of agreement. The correlation coefficient for Kuhn's angle was 0.29, indicating poor reliability. Power's ratio	

							showed the poorest level of agreement, with a correlation coefficient of 0.03 in flexion and 0.06 in extension.	

Wu 2015	38 asymptomatic participants and 38 disc-herniated patients with average ages of 28.3 +/- 3.9 and 31.2 +/- 4.6 years. The body heights and weights were 167.6 +/- 6.5 cm and 62.8 +/- 6.2.3 kg for asymptomatic subjects, and 169.8 +/- 7.4 cm and 64.6 +/- 7.2 kg for male subjects.	2	The intra-observer errors for digitizing procedures were examined at a 2-week interval and the interobserver errors were calculated from the measurements made by two observers.<br>In all, two sets of 608 image sequences were digitized by two experienced research members of spine laboratory and the activities for identifying vertebral landmarks were blinded. These vertebral landmarks were digitized three times by each observer, and the	Videofluoroscopic image sequences from the different motion ranges of lateral cervical flexion and extension	Geometric midplanes method and rotation matrix method for measuring intervertebral translation.	The intra- and interobserver reproducibility on measuring the sequential translations were in the acceptable range for geometric midplanes method (average intraclass correlation coefficients [ICCs], 0.860 and 0.806; mean absolute difference [MAD] 0.19 and 0.33 mm) and rotation matrix method (average ICCs, 0.807 and 0.735; MAD, 0.35 and 0.42 mm). There was significantly better reproducibility on the	The intra- and interobserver reproducibility on measuring the sequential translations were in the acceptable range for geometric midplanes method (average intraclass correlation coefficients [ICCs], 0.860 and 0.806; mean absolute difference [MAD] 0.19 and 0.33 mm) and rotation matrix method (average ICCs, 0.807 and 0.735; MAD, 0.35 and 0.42 mm). There was significantly better reproducibility on the	

			mean values were used for subsequent analysis.			measurements of intervertebral translation for the geometric midplanes method than those of rotation matrix method (p5.001.040). The absolute mean differences of the translation measurements between two image protocols averaged 11.2% and 10.8% for the asymptomatic subjects and disc-herniated patients, respectively.	measurements of intervertebral translation for the geometric midplanes method than those of rotation matrix method (p5.001.040). The absolute mean differences of the translation measurements between two image protocols averaged 11.2% and 10.8% for the asymptomatic subjects and disc-herniated patients, respectively.	

Zhang 2022	100 patients, 45 males, 55 females, average age 57.14±11.05 years old.	2	One attending spine surgeon as the experienced observer (observer A) and one orthopaedic resident in training as the inexperienced observer (observer B) independently reviewed the radiographs. The observers were blinded to the patient information. Observer A and observer B both took photos from the iPhone and measured the angles independently. To evaluate the interobserver reliability, the cervical sagittal parameters were	Lateral plain film cervical radiographs	Four angles, including the C0-2 Cobb angle, C2-7 Cobb angle, T1S and neck tilt (NT), were assessed by iPhone and PACS.	The ICC of interobserver reliability of the C0-2 Cobb angle was 0.953 (0.930–0.968), and the Bland–Altman plot revealed that the 95% CI was 4.4°, while the mean difference was 0.2°. The ICC of interobserver reliability of the C2-7 Cobb angle was 0.972 (0.959–0.981), and the Bland–Altman plot revealed that the 95% CI was 4.4°, while the mean difference was 0.3° (Fig. 5B). The ICC of interobserver reliability of T1S was 0.957	The ICC of the intraobserver reliability of the C0-2 Cobb angle was 0.966 (0.950–0.977), and the Bland–Altman plot revealed that the 95% CI was 3.8°, while the mean difference was 0.1°. The ICC of the intraobserver reliability of the C2-7 Cobb angle was 0.983 (0.974–0.988), and the Bland–Altman plot revealed that the 95% CI was 3.5°, while the mean difference was 0.0° (Fig. 4B). The ICC of intraobserver reliability of T1S was 0.971	

			measured with the iPhone's intrinsic photo edit function by two of the observers. Observer A measured the angles with the iPhone again to evaluate the intraobserver reliability and with the PACS to assess the validity of the novel iPhone method. The interval between each round of measurements was more than two weeks. The order of the radiographs was reorganized randomly to decrease possible recall. A stopwatch was used to record the			(0.937–0.971), and the Bland–Altman plot revealed that the 95% CI was 4.3°, while the mean difference was 0.3°. The ICC of interobserver reliability of NT was 0.929 (0.897–0.952), and the Bland–Altman plot revealed that the 95% CI was 4.9°, while the mean difference was 0.4°.	(0.958–0.981), and the Bland–Altman plot revealed that the 95% CI was 3.5°, while the mean difference was 0.2°. The ICC of intraobserver reliability of NT was 0.951 (0.928–0.967), and the Bland–Altman plot revealed that the 95% CI was 4.2°, while the mean difference was 0.0°.	

			time of each measurement. To simulate the actual measurement process, the time of measurement by the PACS began with the input of the radiograph number into the PACS and ended with the recording of the angle. The time of measurement of the iPhone began with the turning on of the camera function and ended with the recording of the angle. Excel 2016 was used to record the angle data.					

Table S3. Sub-Group Analysis of Intra-examiner Reliability Studies on Biomechanical Mensuration Methods of Sagittal Cervical Radiography by Intra-examiner Reliability Statistics, Study Quality, SEM, and MAD

Mensuration Method	Low Quality	Intra-Examiner Reliability Statistics	Moderate Quality	Intra-Examiner Reliability Statistics	High Quality	Intra-Examiner Reliability Statistics	Intra-Examiner Reliability Quality	SEM, MAD
**Cobb Method (C2–C7) (°)**	n = 3 (Paholpak 2018, Plaugher 1990, Salameh 2021)	ICC = 0.762–0.968 (Paholpak 2018), > 0.80 (Salameh 2021)	n = 4 (Gwinn 2009, Barth 2017, Lafage 2015, Shin 2016)	ICC = 0.838 (Gwinn 2009), 0.929 (Lafage 2015), 0.95 (Shin 2016)	n = 4 (Harrison 2000, Janusz 2016, Lee 2017, Zhang 2022)	ICC = 0.95 (Harrison 2000), 0.96 (Janusz 2016), 0.876, 0.921, 0.999 (Lee 2017), 0.983 (Zhang 2022)	Good (Salemeh 2021, Lee 2017, Paholpak 2018); Excellent (Gwinn 2009, Plaugher 1990, Janusz 2016, Lee 2017, Shin 2016, Zhang 2022)	MAD (°) = 0.8 (Lafage 2015), 3.8 (Shin 2016); SEM (°) = 5.47 (Gwinn 2009), 2.06 (Janusz 2016)
		Pearson's r = 0.97 (Plaugher 1990)		kappa = > 0.90 (Gwinn 2009)				
**Cobb Method (C1–C7) (°)**	n = 0	X	n = 2 (Champain 2006, Shin 2016)	ICC = 0.99 - 1.00 (Champain 2006), 0.96 (Shin 2016)	n = 1 (Harrison 2000)	ICC = 0.91 (Harrison 2000)	Excellent (Champain 2006, Harrison 2000, Shin 2016)	MAD (°) = 2.9 (Shin 2016)
**Posterior Tangent (C2–C7) (°)**	n = 0	X	n = 4 (Gadotti 2013a, Gadotti 2013b, Gwinn 2009, Shin 2016)	ICC = 0.99 (Gandotti 2013a), 0.99, 0.94 (Gandotti 2013b), 0.927 (Gwinn 2009), 0.96 (Shin 2016)	n = 2 (Harrison 2000, Jackson 1993, Janusz 2016)	ICC = 0.95 (Harrison 2000), 0.82–0.99 (Jackson 1993), 0.96 (Janusz 2016)	Excellent (Gandotti 2013a, Gandottib, Gwinn 2009, Harrison 2000, Jackson 1993, Janusz	SEM (°) = 2.65 (Gwinn 2009), 0.99 (Gandotti 2013a), 0.50, 0.99 (Gandotti 2013b), 1.99 (Janusz 2016); MAD (°) = 3.1 (Shin 2016),

							2016, Shin 2016)	
**C0–C2 (°)**	n = 1 (Salameh 2021)	ICC = 0.57 (Salameh 2021)	n = 1 (Chamnan 2020)	ICC = 0.87–0.95 (Chamnan 2020)	n = 1 (Zhang 2022)	ICC = 0.966 (Zhang 2022)	Moderate (Salameh 2021); Good to Excellent (Chamnan 2020, Zhang 2022)	Not Reported
**Sagittal Vertical Axis (C2–C7) (mm)**	n = 1 (Salameh 2021)	ICC = 0.95 (Salameh 2021)	n = 2 (Champain 2006, Lafage 2015)	ICC = 0.967 (Lafage 2015)	n = 1 (Lee 2017)	ICC = 0.973, 0.981, 0.999 (Lee 2017)	Excellent (Lee 2017, Lafage 2015, Salameh 2021)	MAD (mm) = 0.3 (Lafage 2015)
				Pearson = (Champain 2006)		Bland-Altman LOA		
**T1 Slope (°)**	n = 1 (Salameh 2021)	ICC = 0.92 (Salameh 2021)	n = 1 (Lafage 2015)	ICC = 0.717 (Lafage 2015)	n = 2 (Lee 2017, Zhang 2022)	ICC = 0.890, 0.960, 0.987 (Lee 2017), 0.971 (Zhang 2022)	Good (Lafage 2015); Excellent (Lee 2017, Salameh 2021, Zhang 2022)	MAD (°) = 1.1 (Lafage 2015)
**Thoracic Inlet Angle (°)**	n = 1 (Salameh 2021)	ICC = 0.86 (Salameh 2021)	n = 0	X	n = 1 (Lee 2017)	ICC = 0.788, 0.907, 0.990 (Lee 2017)	Good (Salameh 2021); Excellent (lee 2017)	Not Reported
**Intersegmental Translation (mm)**	n = 1 (Svedmark 2011)	Mean diff (95% CI) = 0.36 (1.24)	n = 2 (Frobin 2002, Wu 2015)	ICC = 0.778–0.934 (Wu 2015)	n = 0	X	Good (Wu 2015)	MAD (mm) = 0.14–0.21 (Wu 2015)

**Intersegmental Rotation (°)**	n = 3 (Hardacker 1997, Paholpak 2018, Svedmark 2011)	ICC = 0.762–0.968 (Paholpak 2018)<br>Pearson r = 0.67–0.96 (Hardacker 1997)<br>Mean diff (95% CI) = 0.06 (1.03)	n = 2 (Barth 2017, Frobin 2002, Wu 2015)	ICC = 0.715–0.833 (Wu 2015)<br>Mean deviation (SD) = 0.429 (0.8588)–1.5717 (0.3078) (Frobin 2002)	n = 5 (Harrison 2000, Herrmann 2004, Jackson 1993, Janusz 2016, Jiang 2014)	ICC = 0.71–0.97 (Harrison 2000), 0.93 (Hermann 2004), 0.774–0.86 (Jackson 1993), 0.83–0.89 (Janusz 2016), 0.61–0.89 (Jiang 2014)	Good (Paholpak 2018, Wu 2015, Harrison 2000, Jackson 1993, Janusz 2016, Jiang 2014); Excellent (Hermann 2004)	SEM (°) = 1.20° (Hermann 2004), 1.48–1.79 (Janusz 2016) 0.54–1.04 (Jackson 1993); MAD (°) = 0.26–0.44 (Wu 2015)

° = degree; mm = millimeter; ICC = intra-class correlation coefficient; n = number; X = no study; SEM = standard error of measurement; MAD = mean absolute deviation; CI = confidence interval; SD = standard deviation

Table S4. Sub-Group Analysis of Inter-examiner Reliability Studies on Biomechanical Mensuration Methods of Sagittal Cervical Radiography by Inter-examiner Reliability Statistics, Study Quality, SEM, and MAD

Mensuration Method	Low Quality	Inter-Examiner Reliability Statistics	Moderate Quality	Inter-Examiner Reliability Statistics	High Quality	Inter-Examiner Reliability Statistics	Inter-Examiner Reliability Quality	SEM, MAD
**Cobb Method (C2–C7) (°)**	n = 4 (Lopez 2021, Paholpak 2018, Plaugher 1990, Salameh 2021)	ICC = 0.90 (Lopez 2021), 0.762–0.968 (Paholpak 2018), >0.80 (Salameh 2021)<br>Pearson's r = 0.89–0.94 (Plaugher 1990)	n = 5 (Cannada 2003, Gwinn 2009, Cote 1997, Lafage 2015, Shin 2016)	ICC = 0.77 (Gwinn 2009), 0.96 (Cote 1997), 0.844 (Lafage 2015), 0.88 (Shin 2016)<br>kappa = 0.933 (Gwinn 2009)<br>Chronbach's a = 0.74 (Cannada 2003)	n = 4 (Harrison 2000, Janusz 2016, Lee 2017, Zhang 2022)	ICC = 0.94 (Harrison 2000), 0.92 (Janusz 2016), 0.770, 0.887, 0.934 (Lee 2017), 0.972 (Zhang 2022)	Good (Gwinn 2009, Shin 2016, Salameh 2021, Paholpak 2018); Excellent (Lee 2017, Cote 1997, Lopez 2021, Plaugher 1990, Janusz 2016, Zhang 2022)	SEM (°) = 7 (Gwinn 2009), 2.71 (Janusz 2016); MAD (°) = 5.0 (Shin 2016)
**Cobb Method (C1–C7) (°)**	n = 0	X	n = 3 (Champain 2006, Cote 1997, Marshall 1996, Shin 2016)	ICC = 0.986–0.999 (Champain 2006), 0.94 (Cote 1997), 0.92 (Shin 2016)<br>Mean signed diff = 0.04 ° (95% CI, -0.07–0.14)	n = 1 (Harrison 2000)	ICC = 0.94 (Harrison 2000)	Excellent (Champain 2006, Cote 1997, Harrison 2000, Marshall 1996, Shin 2016)	MAD (°) = 3.4 (Shin 2016), 0.9 (Marshall 1996)

**Posterior Tangent (C2–C7) (°)**	n = 1 (Donk 2017)	Chronbach's a = 0.907 (Donk 2017)	n = 3 (Gadotti 2013b, Gwinn 2009, Shin 2016)	ICC = 0.87 (Shin 2016), 0.90 (Gandotti 2013b), 0.867 (Gwinn 2009)<br>kappa = 0.933 (Gwinn 2009)	n = 3 (Harrison 2000, Jackson 1993, Janusz 2016)	ICC = 0.92 (Harrison 2000), 0.96 (Jackson 1993), 0.94 (Janusz)	Good (Shin 2016, Gwinn 2009); Exellent (Donk 2017, Janusz 2016, Gandotti 2013b, Harrison 2000, Jackson 1993)	SEM (°) = 1.20 (Jackson 1993), 2.62 (Janusz), 3.78 (Gandotti 2013b), 4.26 (Gwinn 2009); MAD (°) = 5.2 (Shin),
**C0–C2 (°)**	n = 2 (Bellebarba 2021, Salameh 2021)	ICC = 0.889 (Bellbarba 2021), 0.57 (Salameh 2021)	n = 1 (Chamnan 2020)	ICC = 0.73–0.89 (Chamnan 2020)	n = 1 (Zhang 2022)	ICC = 0.953 (Zhang 2022)	Moderate (Salameh 2021); Good (Chamnan 2020, Bellbarba 2021); Excellent (Zhang 2022)	Not Reported
**Sagittal Vertical Axis (C2–C7) (mm)**	n = 1 (Salameh 2021)	ICC = 0.95 (Salameh 2021	n = 2 (Champain 2006, Lafage 2015)	ICC = 0.914 (Lafage 2015)	n = 1 (Lee 2017)	ICC = 0.909, 0.911, 0.983 (Lee 2017)	Excellent (Lee 2017, Salameh 2021, Lafage 2015)	Not Reported
**T1 Slope (°)**	n = 1 (Salameh 2021)	ICC = 0.92 (Salameh 2021)	n = 1 (Lafage 2015)	ICC = 0.895 (Lafage 2015)	n = 2 (Lee 2017, Zhang 2022)	ICC = 0.884, 0.886, 0.979 (Lee 2017), 0.957	Good (Lafage 2015); Excellent (Lee 2017, Salameh	Not Reported

						(Zhang 2022)	2021, Zhang 2022)	
**Thoracic Inlet Angle (°)**	n = 1 (Salameh 2021)	ICC = 0.86 (Salameh 2021)	n = 0	X	n = 1 (Lee 2017)	ICC = 0.795, 0.851, 0.945 (Lee 2017)	Good (Salameh 2021); Good-to-Excellent (Lee 2017)	Not Reported
**Intersegmental Translation (mm)**	n = 0	X	n = 3 (Bono 2011, Frobin 2002, Wu 2015)	ICC = 0.87 (Bono 2011), 0.735 - 0.885 (Wu 2015)	n = 0	X	Good (Bono 2011, Wu 2015)	MAD (mm) = 0.23–0.46 (Wu 2015)
				Mean deviation (SD) = 0.0002 (0.0288)–0.0196 (0.0339) (Frobin 2002)				
**Intersegmental Rotation (°)**	n = 2 (Hardacker 1997, Paholpak 2018)	ICC = 0.762–0.968 (Paholpak 2018)	n = 3 (Dvorak, 1988 Frobin 2002, Wu 2015)	ICC = 0.715–0.892 (Wu 2015)	n = 5 (Alhilali 2013, Harrison 2000, Herrmann 2004, Jackson 1993, Janusz 2016, Jiang 2014)	ICC = 0.66–0.84 (Harrison 2000), 0.86 (Hermann 2004), 0.77–0.92 (Janusz 2016), 0.81–0.96 (Jiang 2014)	Good (Paholpak 2018, Hardacker 1997, Wu 2015, Harrison 2000, Hermann 2004, Janusz 2016, Jiang 2014)	SEM (°) = 1.77 (Hermann 2004), 1.52–2.09 (Janusz), 0.54–1.04 (Jackson 1993); MAD (°) = 0.39–0.52 (Wu 2015)
		Pearson's r = 0.64–0.92		Student t test = 0.0001–0.0005				

		(Hardacker 1997)		(Dvorak 1988)				
				Mean deviation (SD) = 0.301 (1.7104)–0.8437 (1.1048) (Frobin 2002)		Lin Correlation Coefficient = 0.9576 (95% CI, 0.9417–0.9692)		

° = degree; mm = millimeter; ICC = intra-class correlation coefficient; n = number; X = no study; SEM = standard error of measurement; MAD = mean absolute deviation; CI = confidence interval; SD = standard deviation
